# Associations of the C‐Reactive Protein−Albumin−Lymphocyte Index, Red Cell Distribution Width−Albumin Ratio, and Blood Urea Nitrogen−Albumin Ratio With All‐Cause and Cardiovascular Mortality Among Adults With Preclinical Heart Failure

**DOI:** 10.1002/clc.70401

**Published:** 2026-07-06

**Authors:** Huifang Su, Xiandong Liu

**Affiliations:** ^1^ Ganzhou People's Hospital Ganzhou China

**Keywords:** blood urea nitrogen–albumin ratio, C‐reactive protein–albumin–lymphocyte index, mortality, preclinical heart failure, red cell distribution width–albumin ratio

## Abstract

**Background:**

Preclinical heart failure (PHF) is an early stage in the heart failure continuum, yet practical indices for mortality risk assessment remain understudied.

**Hypothesis:**

The C‐reactive protein–albumin–lymphocyte index (CALLY), red cell distribution width–albumin ratio (RAR), and blood urea nitrogen–albumin ratio (BAR) are associated with mortality and provide additional predictive information among adults with PHF.

**Methods:**

We analyzed adults aged ≥ 20 years with PHF from the National Health and Nutrition Examination Survey (NHANES) 1999–2010. PHF was defined by hypertension, diabetes, obesity, atherosclerotic cardiovascular disease, or prior myocardial infarction, without diagnosed heart failure. The indices were derived from baseline laboratory measurements and log‐transformed. Mortality was ascertained through December 31, 2019. Associations were assessed using survey‐weighted Cox models; nonlinearity, subgroup consistency across different PHF compositions, incremental predictive performance, and sensitivity analyses, including competing‐risk models, were evaluated.

**Results:**

Among 14 717 adults with PHF contributing 186 678.5 person‐years, 3801 all‐cause and 1226 cardiovascular deaths occurred. Per standard deviation increase, ln CALLY was associated with lower all‐cause and cardiovascular mortality (HRs, 0.85 and 0.87), whereas ln RAR (HRs, 1.28 and 1.24) and ln BAR (HRs, 1.09 and 1.18) were associated with higher mortality. ln RAR showed the most consistent improvement in exploratory incremental predictive performance beyond the base model. The directions of association were broadly consistent across different PHF compositions, and the findings remained robust in sensitivity analyses.

**Conclusions:**

CALLY, RAR, and BAR were independently associated with mortality in adults with PHF; RAR may improve mortality risk assessment.

## Graphical abstract

In adults with preclinical heart failure from NHANES, higher ln CALLY was associated with lower all‐cause and cardiovascular mortality, whereas higher ln RAR and ln BAR were associated with higher mortality. Among these indices, ln RAR showed the most consistent exploratory incremental predictive performance beyond the base model.

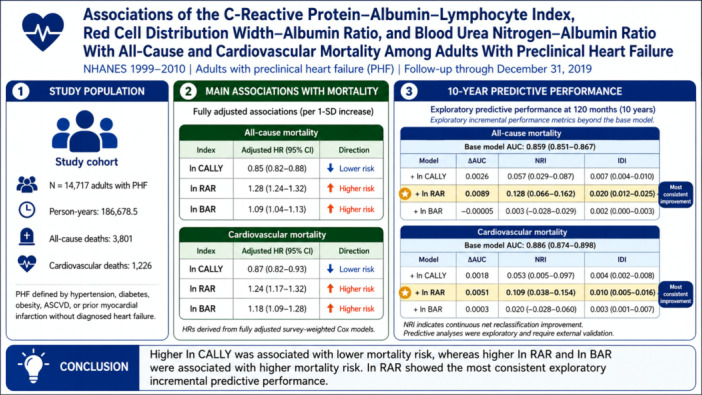

## 1 Introduction

Heart failure (HF) is a complex clinical syndrome resulting from structural or functional cardiac abnormalities and remains a major cause of morbidity and mortality worldwide [1]. Despite improvements in prevention and treatment, the burden of HF continues to increase because of population aging and the rising prevalence of cardiometabolic risk factors [2, 3]. Preclinical heart failure (PHF) represents an early stage in the HF continuum, encompassing individuals with major cardiovascular risk factors or structural cardiac abnormalities in the absence of diagnosed HF [4]. A nationally representative analysis of US adults showed that PHF was highly prevalent and associated with higher risks of all‐cause and cardiovascular mortality [5]. These findings highlight the need for simple and accessible indicators to improve mortality risk assessment in this population.

Systemic inflammation, nutritional impairment, immune dysregulation, hematologic abnormalities, and renal‐metabolic dysfunction may contribute to adverse cardiovascular outcomes and HF progression [6]. Chronic low‐grade inflammation is associated with endothelial dysfunction, myocardial remodeling, fibrosis, and atherosclerotic progression, whereas hypoalbuminemia may reflect both malnutrition and systemic inflammatory burden [7, 8]. In addition, alterations in lymphocyte count, red cell distribution width, and renal function may provide complementary information regarding systemic vulnerability [9]. Composite laboratory indices integrating these biological domains may therefore offer more comprehensive prognostic information than individual laboratory parameters alone.

The C‐reactive protein–albumin–lymphocyte index (CALLY) integrates inflammation, nutritional status, and immune function [10]. The red cell distribution width–albumin ratio (RAR) reflects hematologic variability together with nutritional and inflammatory status [11], whereas the blood urea nitrogen–albumin ratio (BAR) captures renal‐metabolic stress and nutritional status [10, 12, 13, 14]. Previous studies have reported associations of RAR and BAR with mortality among patients with HF [12, 13], whereas the prognostic value of CALLY has been primarily investigated in other clinical populations [15]. However, the associations of these three composite laboratory indices with mortality among adults with PHF remain unclear.

Moreover, the comparative predictive performance of CALLY, RAR, and BAR in PHF has not been systematically evaluated. It remains uncertain whether their associations with mortality are linear, whether threshold effects exist, and whether any of these indices provide additional predictive information beyond conventional risk factors. Therefore, using data from the National Health and Nutrition Examination Survey (NHANES) 1999–2010, we investigated the associations of CALLY, RAR, and BAR with all‐cause and cardiovascular mortality among adults with PHF. We further evaluated potential nonlinear associations, threshold effects, subgroup consistency, and incremental predictive performance beyond conventional risk factors.

## 2 Methods

### 2.1 Study Population and Design

**Figure 1:**
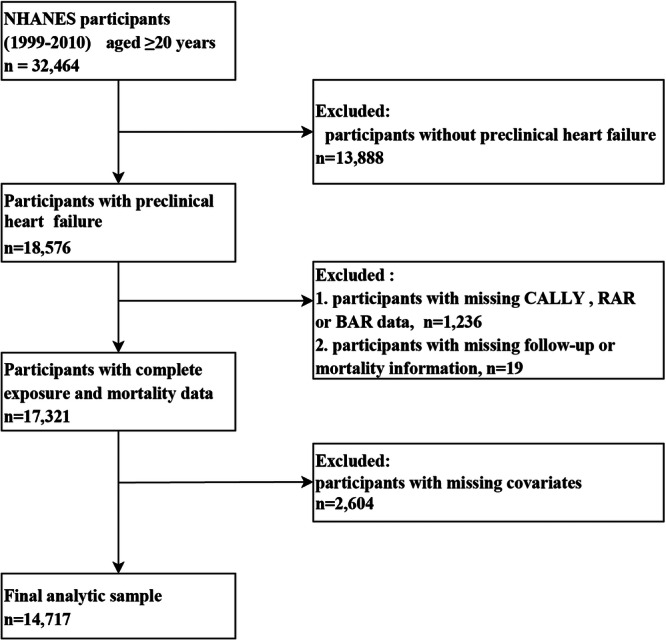
Flowchart of participant selection among US adults aged 20 years or older with preclinical heart failure in the National Health and Nutrition Examination Survey, 1999–2010. NHANES, National Health and Nutrition Examination Survey; PHF, preclinical heart failure.

We conducted a population‐based cohort analysis using data from six cycles of NHANES conducted between 1999 and 2010. The primary analysis included participants from the NHANES 1999 to 2010 cycles because calculation of the CALLY requires serum C‐reactive protein (CRP). Conventional CRP measurements were consistently available across these cycles. However, CRP data required for the calculation of CALLY were unavailable in the NHANES 2011–2014 cycles, whereas high‐sensitivity CRP (hs‐CRP) measurements subsequently became available in the NHANES 2015–2018 cycles. We did not directly pool the NHANES 1999–2010 and 2015–2018 cycles because of discontinuities in CRP availability and potential measurement heterogeneity arising from the use of conventional CRP versus hs‐CRP. In addition, participants enrolled during 2015–2018 had substantially shorter mortality follow‐up. NHANES is a series of cross‐sectional, nationally representative surveys designed by the National Center for Health Statistics (NCHS) to assess the health and nutritional status of the civilian, noninstitutionalized US population. Mortality follow‐up was obtained through linkage to the National Death Index. The NHANES protocols were approved by the NCHS Ethics Review Board, and all participants provided written informed consent. Detailed information on the survey design, sampling procedures, and data collection is available from the NCHS documentation [16]. Mortality follow‐up was obtained from the Continuous NHANES Public‐use Linked Mortality Files, 2019 [17]. Adults aged ≥ 20 years were eligible for the present analysis if they met the criteria for PHF. Participants were excluded if they had missing data required to calculate CALLY, RAR, or BAR; missing mortality follow‐up information; or missing covariate data included in the primary analysis. The participant selection process is presented in Figure 1.

### 2.2 Definition of PHF

PHF was defined according to the operational criteria used in a previous NHANES‐based study based on the American Heart Association guideline [5]. Participants were classified as having PHF if they had at least one of the following components: hypertension, diabetes mellitus, obesity, atherosclerotic cardiovascular disease (ASCVD), or a history of myocardial infarction as an indicator of structural cardiac abnormality. Hypertension was defined as systolic blood pressure ≥ 130 mmHg, diastolic blood pressure ≥ 80 mmHg, or self‐reported use of antihypertensive medication [18]. Obesity was defined as a body mass index (BMI) ≥ 30 kg/m^2^. Diabetes mellitus was defined as fasting plasma glucose ≥ 126 mg/dL, glycated hemoglobin (HbA1c) ≥ 6.5%, or self‐reported use of glucose‐lowering medication [19]. ASCVD was defined as self‐reported coronary heart disease, stroke, or angina. A history of myocardial infarction was identified based on self‐reported physician diagnosis. Participants with self‐reported physician‐diagnosed congestive HF were excluded to restrict the study population to individuals without diagnosed HF.

### 2.3 Assessment of Composite Laboratory Indices

CALLY, RAR, and BAR were calculated using baseline laboratory measurements obtained during the NHANES examination [10, 12, 13]. CALLY was calculated as serum albumin (g/dL) × lymphocyte count (10^9^/L)/CRP (mg/L); RAR was calculated as red cell distribution width (%)/serum albumin (g/dL); and BAR was calculated as blood urea nitrogen (mg/dL)/serum albumin (g/dL). Because of skewed distributions, CALLY, RAR, and BAR were natural log‐transformed as ln CALLY, ln RAR, and ln BAR, respectively. For continuous, restricted cubic spline, and threshold analyses, the log‐transformed indices were standardized as *z* scores, with hazard ratios (HRs) and inflection points expressed per SD and in SD units, respectively. For categorical analyses, the log‐transformed indices were categorized into quartiles, with the lowest quartile as the reference. Detailed laboratory methods and quality‐control procedures are available in the NHANES documentation.

### 2.4 Mortality Outcomes

Mortality status was ascertained through linkage to the National Death Index, with follow‐up through December 31, 2019. The outcomes of interest were all‐cause mortality and cardiovascular mortality. Follow‐up duration was based on the number of months from the baseline Mobile Examination Center (MEC) examination to death or December 31, 2019, whichever occurred first, as provided in the linked mortality file, and was converted to years for analysis. Cardiovascular mortality was defined as death from heart disease or cerebrovascular disease, identified according to the underlying cause of death using International Classification of Diseases, 10th Revision (ICD‐10) codes I00–I09, I11, I13, I20–I51, and I60–I69.

### 2.5 Covariates

Covariates were selected a priori based on previous literature and clinical relevance. They included age, sex, race/ethnicity, education level, marital status, poverty income ratio (PIR), smoking status, alcohol consumption, physical activity, BMI, hypertension, diabetes mellitus, hyperlipidemia, coronary heart disease, angina, stroke, history of myocardial infarction, history of cancer, alanine aminotransferase (ALT), aspartate aminotransferase (AST), total bilirubin, estimated glomerular filtration rate (eGFR), and hemoglobin. Race/ethnicity was categorized as non‐Hispanic White, non‐Hispanic Black, Mexican American, other Hispanic, or other race. Education level was categorized as less than 9th grade, 9th–11th grade, high school graduate or equivalent, some college or associate degree, or college graduate or above. Marital status was categorized as living with a partner or not living with a partner. PIR was categorized as ≤ 1.30, 1.31–3.50, or > 3.50. Smoking status was classified as never, former, or current smoking: never smokers had smoked < 100 cigarettes during their lifetime; former smokers had smoked ≥ 100 cigarettes but did not currently smoke; and current smokers had smoked ≥ 100 cigarettes and currently smoked every day or on some days. Alcohol consumption was classified as never, former, or current drinking: never drinkers had consumed < 12 alcoholic drinks in any 1 year; former drinkers had consumed ≥ 12 drinks in any 1 year but had not consumed alcohol during the previous year; and current drinkers reported alcohol consumption during the previous year. Participants reporting ≥ 150 min of moderate‐intensity equivalent physical activity per week were classified as active; all others were classified as inactive [20]. Hypertension and diabetes mellitus were defined as described above for PHF classification. Hyperlipidemia was defined as lipid‐lowering medication use, triglycerides ≥ 150 mg/dL, total cholesterol ≥ 200 mg/dL, low‐density lipoprotein cholesterol ≥ 130 mg/dL, or low high‐density lipoprotein cholesterol (< 40 mg/dL in men or < 50 mg/dL in women). Coronary heart disease, angina, stroke, history of myocardial infarction, and history of cancer were identified based on self‐reported physician diagnosis. eGFR was calculated from serum creatinine using the Chronic Kidney Disease Epidemiology Collaboration equation [21].

### 2.6 Statistical Analysis

Primary descriptive and regression analyses incorporated the complex survey design of NHANES, including sampling weights, strata, and primary sampling units. Because six 2‐year NHANES cycles from 1999 to 2010 were combined, 12‐year weights were calculated by dividing the corresponding 2‐year weights by six. For analyses restricted to alternative combinations of survey cycles, the combined sampling weights were recalculated according to the number of 2‐year cycles included. Continuous variables were summarized as weighted means with standard deviations (SD) or medians with interquartile ranges, as appropriate, and categorical variables were summarized as weighted percentages. Baseline characteristics were compared across quartiles of ln CALLY using survey‐weighted analysis of variance or Rao–Scott chi‐square tests, as appropriate. The primary analyses were conducted using complete cases.

Survey‐weighted Cox proportional hazards models were used to examine the associations of ln CALLY, ln RAR, and ln BAR with all‐cause and cardiovascular mortality. In the analyses of cardiovascular mortality, noncardiovascular deaths were treated as censoring events. The three indices were evaluated both as continuous variables, expressed per 1‐SD increase, and as categorical variables according to quartiles. The proportional hazards assumption was assessed using Schoenfeld residuals. Four sequential models were constructed: Model 1 was unadjusted; Model 2 was adjusted for age, sex, race/ethnicity, education level, marital status, and poverty‐to‐income ratio; Model 3 was further adjusted for smoking status, alcohol consumption, and physical activity; and Model 4 was additionally adjusted for BMI, hypertension, diabetes mellitus, hyperlipidemia, coronary heart disease, angina, stroke, history of myocardial infarction, history of cancer, ALT, AST, total bilirubin, eGFR, and hemoglobin. Results were reported as HRs with 95% confidence intervals (CIs).

Restricted cubic spline analyses of standardized ln CALLY, ln RAR, and ln BAR were performed with four knots placed at the 5th, 35th, 65th, and 95th percentiles to assess potential nonlinear associations with mortality outcomes. When evidence of nonlinearity was observed, two‐piecewise Cox proportional hazards regression models were used to evaluate threshold effects, with inflection points expressed in SD units. Model fit was compared using likelihood‐ratio tests. Weighted Kaplan–Meier curves were generated according to quartiles of each index, and survival differences were assessed using log‐rank tests.

Prespecified subgroup analyses were performed according to age, sex, diabetes mellitus, and stroke history, with interactions evaluated using likelihood‐ratio tests. To further examine the consistency of the associations across different PHF compositions, additional exploratory subgroup analyses were conducted among participants meeting each PHF‐defining criterion, including obesity, hypertension, diabetes mellitus, and ASCVD, as well as the number of PHF components (1, 2, or ≥ 3). These component‐status subgroups were not mutually exclusive because participants could meet more than one PHF‐defining criterion. Additional phenotype‐specific analyses were conducted among participants with only one PHF‐defining criterion. These analyses used the same fully adjusted model as the primary analyses.

The incremental predictive performance of CALLY, RAR, and BAR was evaluated at 60 and 120 months. The base model was specified a priori with reference to established cardiovascular risk‐prediction frameworks, including the ACC/AHA Pooled Cohort Equations and the American Heart Association PREVENT equations, and was adapted to the PHF setting while maintaining model parsimony and clinical interpretability. It included age, sex, race/ethnicity, BMI, smoking status, hypertension, diabetes mellitus, hyperlipidemia, stroke, and eGFR. Hypertension and hyperlipidemia were included as clinically interpretable indicators of the blood pressure and lipid domains, respectively. BMI and eGFR were retained to reflect cardiovascular‐kidney‐metabolic risk, whereas stroke was included to reflect pre‐existing cerebrovascular disease burden [22, 23, 24]. The same base model was applied consistently to each index to enable a fair comparison of their incremental predictive performance. Direct laboratory components of the composite indices were not included in the base model because their inclusion could obscure the incremental contribution of the composite indices. Because predictive performance was evaluated within the same study cohort without external validation, these analyses were considered exploratory. Time‐dependent areas under the curve (AUCs) at 60 and 120 months were estimated using a Cox‐adjusted approach. Changes in AUC, continuous net reclassification improvement (NRI), and integrated discrimination improvement (IDI) were assessed using 500 bootstrap resamples. Decision curve analysis and calibration curves were used to evaluate clinical net benefit and model calibration, respectively. Because predictive performance was evaluated within the same study cohort without external validation, these analyses were considered exploratory.

Sensitivity analyses were conducted to assess the robustness of the primary findings. For cardiovascular mortality, Fine–Gray competing‐risk models were fitted with noncardiovascular death treated as a competing event. To assess whether baseline medication use influenced the primary findings, we conducted a separate sensitivity analysis in which Model 4 was further adjusted for baseline antihypertensive medication use, glucose‐lowering medication use, and lipid‐lowering medication use. Other sensitivity analyses included further adjustment of Model 4 for natural log‐transformed N‐terminal pro‐B‐type natriuretic peptide (ln NT‐proBNP) among participants from the NHANES 1999 to 2004 cycles with available NT‐proBNP measurements, to further account for baseline cardiac stress given the clinical relevance of NT‐proBNP as a biomarker of myocardial wall stress in PHF assessment [25], exclusion of deaths occurring within the first 2 years of follow‐up; and multiple imputation for missing covariate data. Missing covariates were imputed using multiple imputation by chained equations, generating five imputed data sets, and estimates were pooled using Rubin's rules.

To assess the potential influence of temporal differences across survey cycles, three additional analyses were performed. First, the primary analyses were repeated after restricting the study population to participants enrolled in the more recent NHANES 2005–2010 cycles. Second, Model 4 was further adjusted for NHANES survey cycle as a categorical variable. Third, an exploratory temporal validation analysis of all‐cause mortality was conducted using the NHANES 2015–2018 cycles, in which CALLY was calculated using hs‐CRP rather than conventional CRP. Because participants enrolled during these cycles had substantially shorter mortality follow‐up, this analysis was considered exploratory.

Boruta feature selection and SHAP summary plots were conducted as exploratory supplementary analyses to examine the relative importance of ln CALLY, ln RAR, ln BAR, and the covariates included in Model 4, as well as their contributions to predicted mortality risk. These analyses were not used for primary statistical inference. All analyses were conducted using R software (version 4.3.2) and FreeStatistics (version 2.3). A two‐sided *p* < 0.05 was considered statistically significant.

## 3 Results

### 3.1 Study Population

A total of 32 464 participants aged ≥ 20 years from NHANES 1999 to 2010 were initially screened. After excluding 13 888 participants who did not meet the PHF definition, 18 576 participants with PHF remained eligible. Participants with missing data required to calculate CALLY, RAR, or BAR (*n* = 1236) or missing mortality follow‐up information (*n* = 19) were subsequently excluded, leaving 17 321 participants with complete exposure and mortality follow‐up data. After further excluding 2604 participants with missing covariate data, 14 717 participants were included in the final analytic cohort. The participant selection process is shown in Figure 1.

### 3.2 Participant Characteristics

Weighted baseline characteristics according to quartiles of ln CALLY are presented in Table 1. The final analytic cohort included 14 717 participants and represented an estimated 98.46 million US adults with PHF. Participants in higher ln CALLY quartiles were younger and more likely to be male, to have higher educational attainment and PIR, to report current alcohol consumption, and to be physically active. In contrast, participants in lower ln CALLY quartiles had higher BMI and higher prevalences of hypertension, diabetes mellitus, hyperlipidemia, stroke, and cancer. Baseline characteristics of participants included in the primary analysis and those excluded because of missing covariate data are presented in Supporting Information S1: Table S1.

**Table 1:** Weighted baseline characteristics of US adults with preclinical heart failure according to quartiles of ln CALLY, NHANES 1999–2010.

Variables	Overall	ln CALLY Q1	ln CALLY Q2	ln CALLY Q3	ln CALLY Q4	*p* value
Unweighted *n*	14 717	3673	3684	3680	3680	
Weighted population, thousands	98 458.0	22 671.9	24 167.6	25 100.0	26 518.5	
Age, years, mean (SD)	50.54 (16.21)	51.46 (16.11)	51.34 (16.13)	50.94 (16.17)	48.65 (16.26)	< 0.001
Sex						< 0.001
Male	50 234.5 (51.0)	8339.4 (36.8)	11 053.5 (45.7)	14 189.1 (56.5)	16 652.4 (62.8)	
Female	48 223.5 (49.0)	14 332.6 (63.2)	13 114.1 (54.3)	10 910.9 (43.5)	9866.0 (37.2)	
Race/ethnicity						< 0.001
Non‐Hispanic White	71 974.5 (73.1)	15 892.2 (70.1)	17 750.3 (73.4)	18 784.0 (74.8)	19 548.0 (73.7)	
Non‐Hispanic Black	11 273.7 (11.5)	3331.1 (14.7)	2834.1 (11.7)	2388.0 (9.5)	2720.5 (10.3)	
Mexican American	6692.2 (6.8)	1701.9 (7.5)	1600.0 (6.6)	1670.5 (6.7)	1719.8 (6.5)	
Other Hispanic	4233.3 (4.3)	1003.0 (4.4)	1169.5 (4.8)	1123.0 (4.5)	937.8 (3.5)	
Other race^a^	4284.2 (4.4)	743.7 (3.3)	813.7 (3.4)	1134.5 (4.5)	1592.3 (6.0)	
Marital status						< 0.001
Living with partner	65 449.3 (66.5)	14 008.3 (61.8)	16 322.6 (67.5)	17 277.9 (68.8)	17 840.4 (67.3)	
Not living with a partner^b^	33 008.7 (33.5)	8663.6 (38.2)	7845.0 (32.5)	7822.1 (31.2)	8678.1 (32.7)	
Poverty income ratio^c^						< 0.001
≤ 1.30	19 521.5 (19.8)	5499.5 (24.3)	4885.4 (20.2)	4594.2 (18.3)	4542.3 (17.1)	
1.31−3.50	36 217.6 (36.8)	8517.1 (37.6)	9141.7 (37.8)	9289.9 (37.0)	9268.8 (35.0)	
> 3.50	42 719.0 (43.4)	8655.3 (38.2)	10 140.5 (42.0)	11 215.9 (44.7)	12 707.3 (47.9)	
Education level						< 0.001
Less than 9th grade	6451.5 (6.6)	1527.1 (6.7)	1762.7 (7.3)	1699.1 (6.8)	1462.6 (5.5)	
9th–11th grade	12 776.8 (13.0)	3372.8 (14.9)	3181.2 (13.2)	3279.3 (13.1)	2943.5 (11.1)	
High school grad/GED or Equivalent	26 438.3 (26.9)	6239.2 (27.5)	6744.4 (27.9)	6927.2 (27.6)	6527.5 (24.6)	
Some college or AA degree	29 910.3 (30.4)	7164.3 (31.6)	7409.2 (30.7)	7195.1 (28.7)	8141.7 (30.7)	
College graduate or above	22 881.1 (23.2)	4368.5 (19.3)	5070.2 (21.0)	5999.2 (23.9)	7443.2 (28.1)	
Smoking status						0.816
Never	49 475.8 (50.3)	11 338.6 (50.0)	12 032.6 (49.8)	12 576.2 (50.1)	13 528.4 (51.0)	
Former	27 783.6 (28.2)	6605.3 (29.1)	6713.9 (27.8)	7107.4 (28.3)	7357.0 (27.7)	
Current	21 198.6 (21.5)	4728.0 (20.9)	5421.1 (22.4)	5416.4 (21.6)	5633.1 (21.2)	
Alcohol consumption						< 0.001
Never	11 965.0 (12.2)	3124.6 (13.8)	3474.7 (14.4)	2626.3 (10.5)	2739.4 (10.3)	
Former	19 031.1 (19.3)	5407.9 (23.9)	4577.8 (18.9)	4848.5 (19.3)	4196.9 (15.8)	
Current	67 461.9 (68.5)	14 139.4 (62.4)	16 115.2 (66.7)	17 625.2 (70.2)	19 582.1 (73.8)	
Physical activity						< 0.001
Inactive	58 333.8 (59.2)	14 740.2 (65.0)	15 165.5 (62.8)	13 822.4 (55.1)	14 605.7 (55.1)	
Active	40 124.2 (40.8)	7931.7 (35.0)	9002.1 (37.2)	11 277.6 (44.9)	11 912.7 (44.9)	
Body mass index, kg/m^2^, mean (SD)	30.72 (6.72)	34.20 (8.04)	31.69 (6.28)	30.08 (5.64)	27.47 (4.93)	< 0.001
Stroke						0.011
No	94 911.8 (96.4)	21 667.3 (95.6)	23 241.1 (96.2)	24 315.0 (96.9)	25 688.4 (96.9)	
Yes	3546.2 (3.6)	1004.6 (4.4)	926.5 (3.8)	784.9 (3.1)	830.1 (3.1)	
Angina						0.533
No	95 363.1 (96.9)	21 878.2 (96.5)	23 368.1 (96.7)	24 368.3 (97.1)	25 748.5 (97.1)	
Yes	3094.9 (3.1)	793.8 (3.5)	799.5 (3.3)	731.6 (2.9)	770.0 (2.9)	
Hyperlipidemia						< 0.001
No	19 145.0 (19.4)	4013.6 (17.7)	4050.3 (16.8)	4425.0 (17.6)	6656.0 (25.1)	
Yes	79 313.0 (80.6)	18 658.3 (82.3)	20 117.3 (83.2)	20 674.9 (82.4)	19 862.4 (74.9)	
Hypertension						0.015
No	43 553.3 (44.2)	9495.5 (41.9)	10 482.8 (43.4)	11 329.7 (45.1)	12 245.3 (46.2)	
Yes	54 904.6 (55.8)	13 176.4 (58.1)	13 684.8 (56.6)	13 770.3 (54.9)	14 273.1 (53.8)	
Diabetes mellitus						< 0.001
No	82 405.6 (83.7)	17 769.4 (78.4)	20 137.6 (83.3)	21 606.9 (86.1)	22 891.8 (86.3)	
Yes	16 052.4 (16.3)	4902.6 (21.6)	4030.0 (16.7)	3493.1 (13.9)	3626.7 (13.7)	
Cancer						0.002
No	88 488.6 (89.9)	20 020.9 (88.3)	21 737.9 (89.9)	22 486.9 (89.6)	24 242.8 (91.4)	
Yes	9969.4 (10.1)	2651.0 (11.7)	2429.7 (10.1)	2613.0 (10.4)	2275.6 (8.6)	
Coronary heart disease						0.722
No	94 452.2 (95.9)	21 826.1 (96.3)	23 114.7 (95.6)	24 071.7 (95.9)	25 439.7 (95.9)	
Yes	4005.8 (4.1)	845.8 (3.7)	1052.9 (4.4)	1028.3 (4.1)	1078.7 (4.1)	
History of myocardial infarction						0.314
No	94 687.9 (96.2)	21 737.4 (95.9)	23 161.2 (95.8)	24 259.1 (96.6)	25 530.2 (96.3)	
Yes	3770.1 (3.8)	934.5 (4.1)	1006.4 (4.2)	840.9 (3.4)	988.3 (3.7)	
eGFR, mL/min/1.73 m^2^, mean (SD)	90.30 (21.30)	90.28 (23.30)	90.17 (21.37)	89.22 (20.59)	91.44 (20.02)	0.001
Hemoglobin, g/dL, mean (SD)	14.51 (1.46)	13.99 (1.45)	14.45 (1.46)	14.70 (1.40)	14.83 (1.41)	< 0.001
Total bilirubin, mg/dL, mean (SD)	0.73 (0.29)	0.65 (0.25)	0.70 (0.28)	0.75 (0.29)	0.79 (0.30)	< 0.001
ALT, U/L, mean (SD)	27.81 (27.70)	27.11 (46.97)	28.24 (19.27)	28.26 (18.89)	27.59 (16.94)	0.426
AST, U/L, mean (SD)	26.11 (15.99)	25.64 (18.54)	26.06 (13.32)	26.44 (19.16)	26.26 (12.11)	0.433
ln RAR, mean (SD)	1.10 (0.12)	1.17 (0.14)	1.11 (0.11)	1.07 (0.10)	1.04 (0.10)	< 0.001
ln BAR, mean (SD)	1.10 (0.37)	1.13 (0.39)	1.11 (0.37)	1.10 (0.36)	1.07 (0.37)	< 0.001

*Note:* Sample sizes are unweighted. Values for categorical variables are presented as weighted population estimates in thousands (weighted percentages), and continuous variables are presented as weighted means (standard deviations). Comparisons across quartiles of ln CALLY were performed using survey‐weighted analysis of variance or Rao–Scott chi‐square tests, as appropriate.

Abbreviations: ALT, alanine aminotransferase; AST, aspartate aminotransferase; BAR, blood urea nitrogen–albumin ratio; CALLY, C‐reactive protein–albumin–lymphocyte index; eGFR, estimated glomerular filtration rate; NHANES, National Health and Nutrition Examination Survey; PIR, poverty income ratio; RAR, red cell distribution width–albumin ratio; SD, standard deviation.

^a^ Included multiracial participants; NHANES did not provide detailed racial/ethnic categories for this group.

^b^ Included participants who were widowed, divorced, separated, or never married.

^c^ Poverty income ratio was categorized as low (≤ 1.30), middle (1.31–3.50), or high (> 3.50).

### 3.3 Associations of ln CALLY, ln RAR, and ln BAR With All‐Cause and Cardiovascular Mortality

During follow‐up, 3801 all‐cause deaths and 1226 cardiovascular deaths occurred. In fully adjusted survey‐weighted Cox models, each 1‐SD increase in ln CALLY was associated with lower risks of all‐cause mortality (HR, 0.85; 95% CI, 0.82–0.88) and cardiovascular mortality (HR, 0.87; 95% CI, 0.82–0.93). In contrast, each 1‐SD increase in ln RAR was associated with higher risks of all‐cause mortality (HR, 1.28; 95% CI, 1.24–1.32) and cardiovascular mortality (HR, 1.24; 95% CI, 1.17–1.32). Similar positive associations were observed for ln BAR, with HRs of 1.09 (95% CI, 1.04–1.13) for all‐cause mortality and 1.18 (95% CI, 1.09–1.28) for cardiovascular mortality (Table 2). In quartile analyses, higher ln CALLY quartiles were consistently associated with lower risks of both outcomes. Higher ln RAR quartiles were associated with increased all‐cause mortality, whereas a significantly higher cardiovascular mortality risk was observed only in the highest quartile. For ln BAR, the highest quartile was associated with increased cardiovascular mortality but not with all‐cause mortality after full adjustment.

**Table 2:** Associations of ln CALLY, ln RAR, and ln BAR with all‐cause and cardiovascular mortality among adults with preclinical heart failure.

Characteristics	Participants, *n*	Events, *n* (%)	Person‐years	Model 1		Model 2		Model 3		Model 4	
				HR (95% CI)	*p* value	HR (95% CI)	*p* value	HR (95% CI)	*p* value	HR (95% CI)	*p* value
All‐cause mortality											
ln CALLY (per SD)	14 717	3801 (25.8)	186 678.5	0.78 (0.75–0.81)	< 0.001	0.83 (0.80–0.86)	< 0.001	0.84 (0.81–0.87)	< 0.001	0.85 (0.82–0.88)	< 0.001
Q1	3673	1154 (31.4)	44 833.7	1.00		1.00		1.00		1.00	
Q2	3684	1000 (27.1)	46 993.7	0.75 (0.68–0.82)	< 0.001	0.78 (0.72–0.85)	< 0.001	0.79 (0.72–0.86)	< 0.001	0.81 (0.75–0.89)	< 0.001
Q3	3680	883 (24)	47 098.4	0.68 (0.60–0.76)	< 0.001	0.67 (0.61–0.73)	< 0.001	0.68 (0.62–0.75)	< 0.001	0.71 (0.65–0.78)	< 0.001
Q4	3680	764 (20.8)	47 752.8	0.54 (0.48–0.61)	< 0.001	0.65 (0.59–0.71)	< 0.001	0.68 (0.62–0.74)	< 0.001	0.67 (0.61–0.74)	< 0.001
ln RAR (per SD)	14 717	3801 (25.8)	186 678.5	1.49 (1.44–1.55)	< 0.001	1.34 (1.30–1.38)	< 0.001	1.33 (1.29–1.37)	< 0.001	1.28 (1.24–1.32)	< 0.001
Q1	3662	594 (16.2)	51 663.8	1.00		1.00		1.00		1.00	
Q2	3309	772 (23.3)	43 191.4	1.60 (1.44–1.78)	< 0.001	1.18 (1.06–1.31)	0.003	1.15 (1.03–1.28)	0.01	1.16 (1.04–1.29)	0.007
Q3	4055	1116 (27.5)	50 195.5	2.03 (1.84–2.25)	< 0.001	1.33 (1.20–1.48)	< 0.001	1.28 (1.15–1.42)	< 0.001	1.28 (1.15–1.42)	< 0.001
Q4	3691	1319 (35.7)	41 627.8	2.97 (2.69–3.27)	< 0.001	1.96 (1.77–2.17)	< 0.001	1.87 (1.69–2.07)	< 0.001	1.76 (1.58–1.97)	< 0.001
ln BAR (per SD)	14 717	3801 (25.8)	186 678.5	1.83 (1.78–1.89)	< 0.001	1.15 (1.11–1.19)	< 0.001	1.17 (1.13–1.21)	< 0.001	1.09 (1.04–1.13)	< 0.001
Q1	3649	533 (14.6)	48 010.0	1.00		1.00		1.00		1.00	
Q2	3661	655 (17.9)	48 566.5	1.21 (1.08–1.35)	0.001	0.87 (0.78–0.98)	0.02	0.91 (0.81–1.02)	0.118	0.91 (0.81–1.02)	0.111
Q3	3698	898 (24.3)	48 549.3	1.66 (1.49–1.85)	< 0.001	0.83 (0.74–0.92)	0.001	0.88 (0.79–0.98)	0.025	0.86 (0.77–0.96)	0.01
Q4	3709	1715 (46.2)	41 552.7	3.85 (3.49–4.24)	< 0.001	1.11 (1.01–1.24)	0.04	1.18 (1.06–1.31)	0.002	1.03 (0.92–1.16)	0.599
Cardiovascular mortality											
ln CALLY (per SD)	14 717	1226 (8.3)	186 678.5	0.82 (0.77–0.87)	< 0.001	0.84 (0.79–0.89)	< 0.001	0.85 (0.80–0.90)	< 0.001	0.87 (0.82–0.93)	< 0.001
Q1	3673	366 (10)	44 833.7	1.00		1.00		1.00		1.00	
Q2	3684	323 (8.8)	46 993.7	0.84 (0.72–0.97)	0.018	0.79 (0.68–0.92)	0.002	0.79 (0.68–0.92)	0.002	0.83 (0.71–0.96)	0.014
Q3	3680	295 (8)	47 098.4	0.76 (0.65–0.89)	0.001	0.70 (0.60–0.81)	< 0.001	0.71 (0.61–0.83)	< 0.001	0.76 (0.65–0.89)	0.001
Q4	3680	242 (6.6)	47 752.8	0.62 (0.52–0.72)	< 0.001	0.65 (0.55–0.77)	< 0.001	0.68 (0.57–0.80)	< 0.001	0.70 (0.59–0.83)	< 0.001
ln RAR (per SD)	14 717	1226 (8.3)	186 678.5	1.39 (1.33–1.46)	< 0.001	1.32 (1.25–1.39)	< 0.001	1.30 (1.24–1.38)	< 0.001	1.24 (1.17–1.32)	< 0.001
Q1	3662	187 (5.1)	51 663.8	1.00		1.00		1.00		1.00	
Q2	3309	266 (8)	43 191.4	1.76 (1.46–2.12)	< 0.001	1.24 (1.03–1.50)	0.026	1.22 (1.01–1.48)	0.037	1.21 (1.00–1.46)	0.052
Q3	4055	341 (8.4)	50 195.5	1.99 (1.66–2.38)	< 0.001	1.23 (1.03–1.48)	0.024	1.19 (0.99–1.43)	0.063	1.16 (0.97–1.40)	0.111
Q4	3691	432 (11.7)	41 627.8	3.11 (2.62–3.70)	< 0.001	1.92 (1.60–2.30)	< 0.001	1.84 (1.54–2.21)	< 0.001	1.65 (1.36–2.00)	< 0.001
ln BAR (per SD)	14 717	1226 (8.3)	186 678.5	2.03 (1.93–2.14)	< 0.001	1.24 (1.17–1.32)	< 0.001	1.26 (1.18–1.34)	< 0.001	1.18 (1.09–1.28)	< 0.001
Q1	3649	138 (3.8)	48 010.0	1.00		1.00		1.00		1.00	
Q2	3661	191 (5.2)	48 566.5	1.36 (1.09–1.69)	0.006	0.96 (0.77–1.2)	0.714	1.00 (0.8–1.25)	0.999	1.01 (0.81–1.26)	0.96
Q3	3698	290 (7.8)	48 549.3	2.06 (1.69–2.53)	< 0.001	0.97 (0.79–1.2)	0.803	1.04 (0.84–1.27)	0.745	1.01 (0.82–1.25)	0.892
Q4	3709	607 (16.4)	41 552.7	5.27 (4.38–6.34)	< 0.001	1.38 (1.13–1.67)	0.001	1.45 (1.19–1.76)	< 0.001	1.27 (1.03–1.57)	0.025

*Note:* Participant counts, event counts, and person‐years are unweighted. HRs and 95% CIs were estimated using survey‐weighted Cox proportional hazards models. For continuous analyses, the log‐transformed indices were standardized as *z* scores and analyzed per 1‐SD increase; for categorical analyses, Q1 was used as the reference group. Model 1 was unadjusted. Model 2 was adjusted for age, sex, race/ethnicity, education level, marital status, and PIR. Model 3 was additionally adjusted for smoking status, alcohol consumption, and physical activity. Model 4 was additionally adjusted for BMI, hypertension, diabetes mellitus, hyperlipidemia, coronary heart disease, angina, stroke, history of myocardial infarction, history of cancer, ALT, AST, total bilirubin, eGFR, and hemoglobin.

Abbreviations: ALT, alanine aminotransferase; AST, aspartate aminotransferase; BAR, blood urea nitrogen–albumin ratio; BMI, body mass index; CALLY, C‐reactive protein–albumin–lymphocyte index; CI, confidence interval; eGFR, estimated glomerular filtration rate; HR, hazard ratio; PIR, poverty income ratio; RAR, red cell distribution width–albumin ratio; SD, standard deviation.

### 3.4 Nonlinear and Threshold Analyses

**Figure 2:**
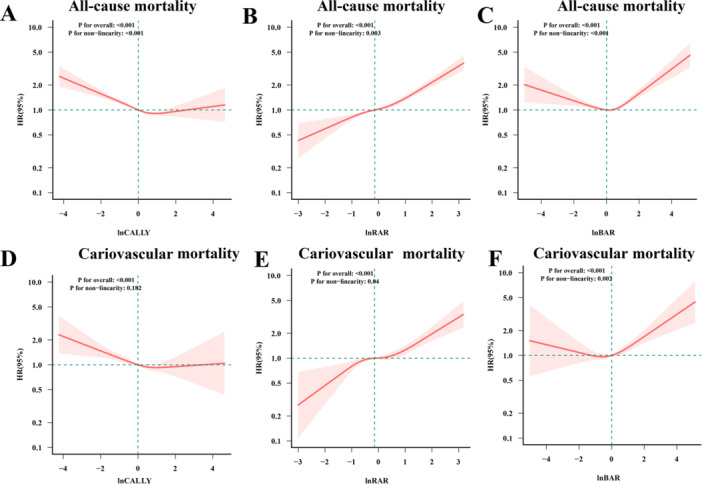
Restricted cubic spline analyses of ln CALLY, ln RAR, and ln BAR in relation to all‐cause and cardiovascular mortality among adults with preclinical heart failure. (A–C) Associations of standardized ln CALLY, ln RAR, and ln BAR with all‐cause mortality, respectively. (D–F) Associations of standardized ln CALLY, ln RAR, and ln BAR with cardiovascular mortality, respectively. Models were adjusted for age, sex, race/ethnicity, education level, marital status, poverty income ratio, smoking status, alcohol consumption, physical activity, body mass index, hypertension, diabetes mellitus, hyperlipidemia, coronary heart disease, angina, stroke, history of myocardial infarction, history of cancer, alanine aminotransferase, aspartate aminotransferase, total bilirubin, estimated glomerular filtration rate, and hemoglobin. BAR, blood urea nitrogen–albumin ratio; CALLY, C‐reactive protein–albumin–lymphocyte index; CI, confidence interval; HR, hazard ratio; RAR, red cell distribution width–albumin ratio; SD, standard deviation.

Restricted cubic spline analyses of standardized ln CALLY, ln RAR, and ln BAR are shown in Figure 2, and the corresponding threshold effect analyses are presented in Table 3. For all‐cause mortality, nonlinear associations were observed for all three indices. Standardized ln CALLY was inversely associated with all‐cause mortality below the inflection point of 0.48 (HR per 1‐SD increase, 0.81; 95% CI, 0.77–0.85), whereas no significant association was observed at or above this threshold. Standardized ln RAR was positively associated with all‐cause mortality on both sides of the inflection point of 0.98, with a stronger association above the threshold. For standardized ln BAR, a significant positive association was observed only at or above the inflection point of 0.37. For cardiovascular mortality, threshold effects were observed for standardized ln RAR and ln BAR. Standardized ln RAR was positively associated with cardiovascular mortality at or above the inflection point of −1.58; however, estimates below this threshold were imprecise and should be interpreted cautiously. Standardized ln BAR was positively associated with cardiovascular mortality at or above the inflection point of −0.13. No threshold effect analysis was reported for standardized ln CALLY because its association with cardiovascular mortality was approximately linear.

**Table 3:** Threshold effect analyses of standardized ln CALLY, ln RAR, and ln BAR in relation to all‐cause and cardiovascular mortality among adults with preclinical heart failure.

Outcome	Variable	Inflection point, SD units	Range, SD units	Adjusted HR per 1‐SD increase (95% CI)	*p* value	*p* for likelihood‐ratio test
All‐cause mortality	ln CALLY	0.48	< 0.48	0.81 (0.77–0.85)	< 0.001	0.001
			≥ 0.48	1.03 (0.89–1.19)	0.71	
	ln RAR	0.98	< 0.98	1.26 (1.18–1.34)	< 0.001	0.002
			≥ 0.98	1.55 (1.34–1.80)	< 0.001	
	ln BAR	0.37	< 0.37	0.93 (0.87–1.00)	0.058	< 0.001
			≥ 0.37	1.31 (1.19–1.44)	< 0.001	
Cardiovascular mortality	ln RAR	−1.58	< −1.58	0.012 (0.00–6.90)	0.96	0.004
			≥ −1.58	1.32 (1.22–1.42)	< 0.001	
	ln BAR	−0.13	< −0.13	1.05 (0.85–1.28)	0.66	0.004
			≥ −0.13	1.28 (1.14–1.45)	< 0.001	

*Note:* Threshold analyses of standardized ln CALLY, ln RAR, and ln BAR were performed using two‐piecewise Cox proportional hazards models. Inflection points and ranges are expressed in SD units, and HRs are reported per 1‐SD increase. Models were fully adjusted for the covariates included in Model 4 of Table 2. Estimates with extremely wide CIs should be interpreted cautiously because of sparse events.

Abbreviations: BAR, blood urea nitrogen–albumin ratio; CALLY, C‐reactive protein–albumin–lymphocyte index; CI, confidence interval; HR, hazard ratio; RAR, red cell distribution width–albumin ratio; SD, standard deviation.

### 3.5 Kaplan–Meier Survival Analyses

**Figure 3:**
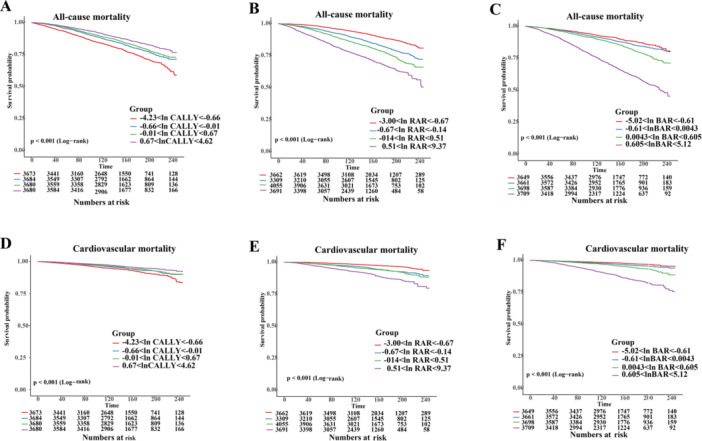
Weighted Kaplan–Meier survival curves for all‐cause and cardiovascular mortality according to quartiles of ln CALLY, ln RAR, and ln BAR. (A–C) Weighted Kaplan–Meier curves for all‐cause mortality according to quartiles of ln CALLY, ln RAR, and ln BAR, respectively. (D–F) Weighted Kaplan–Meier curves for cardiovascular mortality according to quartiles of ln CALLY, ln RAR, and ln BAR, respectively. Survival differences across quartiles were assessed using weighted log‐rank tests. BAR, blood urea nitrogen–albumin ratio; CALLY, C‐reactive protein–albumin–lymphocyte index; KM, Kaplan–Meier; RAR, red cell distribution width–albumin ratio.

Weighted Kaplan–Meier survival curves demonstrated significant differences in survival probability across quartiles of ln CALLY, ln RAR, and ln BAR for both all‐cause and cardiovascular mortality (Figure 3). Higher quartiles of ln CALLY were associated with better survival, whereas higher quartiles of ln RAR and ln BAR were associated with poorer survival. These findings were consistent with the directions of association observed in the survey‐weighted Cox regression analyses.

### 3.6 Exploratory Incremental Predictive Performance

**Figure 4:**
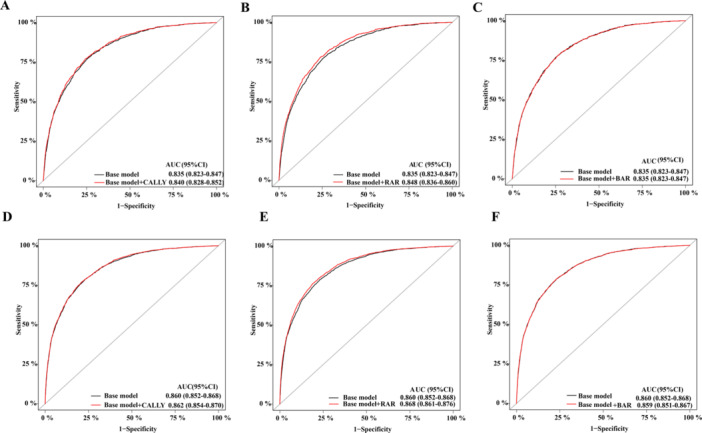
Time‐dependent receiver operating characteristic curves for all‐cause mortality prediction at 60 and 120 months. (A–C) Comparisons of the base model with models additionally including ln CALLY, ln RAR, and ln BAR for 60‐month all‐cause mortality prediction, respectively. (D–F) Corresponding comparisons for 120‐month all‐cause mortality prediction. AUCs with 95% CIs are displayed in each panel. AUC, area under the receiver operating characteristic curve; BAR, blood urea nitrogen–albumin ratio; CALLY, C‐reactive protein–albumin–lymphocyte index; CI, confidence interval; RAR, red cell distribution width–albumin ratio; ROC, receiver operating characteristic.

The exploratory incremental predictive performance of adding ln CALLY, ln RAR, or ln BAR separately to the base model is summarized in Table 4. For all‐cause mortality, the base model yielded AUCs of 0.835 and 0.859 at 60 and 120 months, respectively. The addition of ln RAR resulted in the largest improvement in discrimination, with ΔAUC values of 0.0137 at 60 months and 0.0089 at 120 months, followed by ln CALLY, whereas ln BAR provided little additional improvement. For cardiovascular mortality, the base model yielded AUCs of 0.867 and 0.886 at 60 and 120 months, respectively. Ln RAR again showed the most consistent improvement in discrimination, with ΔAUC values of 0.0064 and 0.0051, respectively, whereas the gains associated with ln CALLY and ln BAR were smaller. Time‐dependent ROC curves for all‐cause and cardiovascular mortality are shown in Figure 4 and Supporting Information S1: Figure S1, respectively.

**Table 4:** Exploratory incremental predictive performance of adding ln CALLY, ln RAR, or ln BAR to the base model for all‐cause and cardiovascular mortality at 60 and 120 months.

Outcome	Prediction horizon	Model	AUC (95% CI)	ΔAUC	*p* for ΔAUC	NRI (95% CI)	*p* for NRI	IDI (95% CI)	*p* for IDI
All‐cause mortality	60 months	Base model	0.835 (0.822−0.847)	Ref	Ref	Ref	Ref	Ref	Ref
		Base model + ln CALLY	0.840 (0.828−0.852)	0.0056	< 0.001	0.103 (0.062−0.141)	< 0.001	0.010 (0.006−0.014)	< 0.001
		Base model + ln RAR	0.848 (0.836−0.860)	0.0137	< 0.001	0.175 (0.108−0.222)	0.004	0.021 (0.012−0.028)	< 0.001
		Base model + ln BAR	0.834 (0.822−0.847)	−0.00002	0.96	0.023 (−0.018−0.054)	0.259	0.002 (0.001−0.004)	0.004
	120 months	Base model	0.859 (0.851−0.867)	Ref	Ref	Ref	Ref	Ref	Ref
		Base model + ln CALLY	0.862 (0.854−0.870)	0.0026	< 0.001	0.057 (0.029−0.087)	< 0.001	0.007 (0.004−0.010)	< 0.001
		Base model + ln RAR	0.868 (0.860−0.876)	0.0089	< 0.001	0.128 (0.066−0.162)	0.004	0.020 (0.012−0.025)	< 0.001
		Base model + ln BAR	0.859 (0.851−0.867)	−0.00005	0.819	0.003 (−0.028−0.029)	0.77	0.002 (0.000−0.003)	0.004
Cardiovascular mortality	60 months	Base model	0.867 (0.849−0.885)	Ref	Ref	Ref	Ref	Ref	Ref
		Base model + ln CALLY	0.870 (0.852−0.888)	0.0024	0.039	0.063 (−0.010−0.120)	0.08	0.002 (0.000−0.005)	0.076
		Base model + ln RAR	0.874 (0.857−0.891)	0.0064	< 0.001	0.142 (0.059−0.210)	0.008	0.006 (0.002−0.012)	< 0.001
		Base model + ln BAR	0.867 (0.849−0.885)	−0.0005	0.575	0.028 (−0.029−0.075)	0.399	0.002 (0.001−0.005)	< 0.001
	120 months	Base model	0.886 (0.874−0.898)	Ref	Ref	Ref	Ref	Ref	Ref
		Base model + ln CALLY	0.888 (0.876−0.900)	0.0018	0.009	0.053 (0.005−0.097)	0.036	0.004 (0.002−0.008)	< 0.001
		Base model + ln RAR	0.891 (0.880−0.903)	0.0051	< 0.001	0.109 (0.038−0.154)	0.020	0.010 (0.005−0.016)	< 0.001
		Base model + ln BAR	0.887 (0.875−0.899)	0.0003	0.580	0.020 (−0.028−0.060)	0.451	0.003 (0.001−0.007)	< 0.001

*Note:* Each log‐transformed index was added separately to the base model, which included age, sex, race/ethnicity, BMI, smoking status, hypertension, diabetes mellitus, hyperlipidemia, stroke, and eGFR. Time‐dependent AUCs were estimated using a Cox‐adjusted approach. ΔAUC, continuous NRI, and IDI were calculated relative to the base model. Confidence intervals for NRI and IDI were estimated using 500 bootstrap resamples. Prediction horizons of 60 and 120 months correspond to 5 and 10 years, respectively. These analyses were exploratory because no external validation was performed.

Abbreviations: AUC, area under the receiver operating characteristic curve; BAR, blood urea nitrogen–albumin ratio; BMI, body mass index; CALLY, C‐reactive protein–albumin–lymphocyte index; CI, confidence interval; eGFR, estimated glomerular filtration rate; IDI, integrated discrimination improvement; NRI, net reclassification improvement; RAR, red cell distribution width–albumin ratio; Ref, reference.

**Figure 5:**
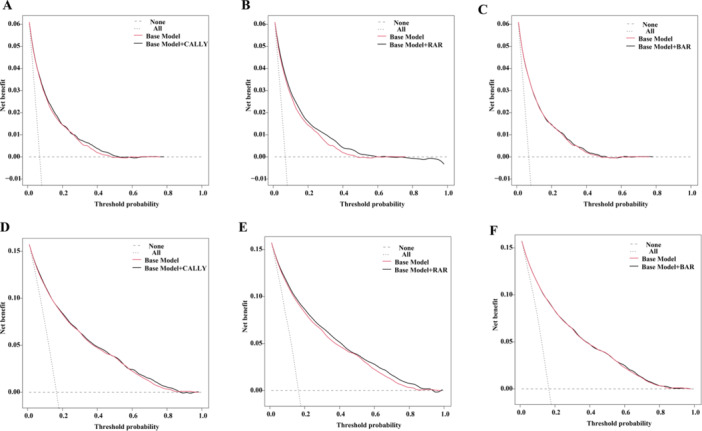
Decision curve analyses for all‐cause mortality prediction at 60 and 120 months. (A–C) Comparisons of the base model with models additionally including ln CALLY, ln RAR, and ln BAR for 60‐month all‐cause mortality prediction, respectively. (D–F) Corresponding comparisons for 120‐month all‐cause mortality prediction. Net benefit is plotted across threshold probabilities. BAR, blood urea nitrogen–albumin ratio; CALLY, C‐reactive protein–albumin–lymphocyte index; DCA, decision curve analysis; RAR, red cell distribution width–albumin ratio.

Decision curve analyses for all‐cause and cardiovascular mortality are presented in Figure 5 and Supporting Information S1: Figure S2, respectively, with quantitative estimates of incremental net benefit summarized in Supporting Information S1: Table S2. For all‐cause mortality prediction, ln RAR provided the greatest and most consistent incremental net benefit among the three indices at both prediction horizons and at both evaluated risk thresholds. For 60‐month all‐cause mortality prediction, the addition of ln RAR yielded ΔNB values of 0.00120 and 0.00143 at risk thresholds of 10% and 20%, respectively, corresponding to net gains equivalent to 0.120 and 0.143 additional true‐positive classifications per 100 individuals after accounting for false‐positive classifications at the respective threshold probabilities. By comparison, the corresponding ΔNB values for ln CALLY were 0.00068 and 0.00102, whereas those for ln BAR were 0.00002 and 0.00003. For cardiovascular mortality prediction, the incremental net benefits of all three indices were generally smaller and varied according to the prediction horizon and risk threshold. Calibration curves demonstrated generally acceptable agreement between predicted and observed 10‐year all‐cause mortality risks (Supporting Information S1: Figure S3).

### 3.7 Subgroup, Sensitivity, and Exploratory Analyses

Prespecified subgroup analyses are presented in Supporting Information S1: Figures S4–S6. Overall, the associations of ln CALLY, ln RAR, and ln BAR with mortality were broadly consistent across most prespecified subgroups. Statistically significant interactions were observed for ln CALLY according to stroke history for both all‐cause and cardiovascular mortality and according to sex for cardiovascular mortality (Supporting Information S1: Figure S4). Significant interactions according to age group were also observed for ln RAR for both mortality outcomes (Supporting Information S1: Figure S5). For ln BAR, an interaction according to stroke history was observed for cardiovascular mortality (Supporting Information S1: Figure S6). Given the exploratory nature of these analyses and the possibility of multiple testing, the subgroup findings should be interpreted cautiously.

Additional exploratory subgroup analyses according to PHF component status and burden are presented in Supporting Information S1: Table S18. For all‐cause mortality, statistically significant interactions were observed between ln CALLY and obesity status and between ln RAR and the number of PHF components (Supporting Information S1: Figures S9 and S10). For cardiovascular mortality, statistically significant interactions were observed between ln CALLY and ASCVD status and between ln RAR and ASCVD status (Supporting Information S1: Figures S11 and S12). No statistically significant interactions were identified for ln BAR in these PHF‐composition analyses. Additional phenotype‐specific analyses among participants with obesity, hypertension, diabetes mellitus, or ASCVD as the only PHF component, as well as analyses stratified by the number of PHF components, are presented in Supporting Information S1: Tables S8–S14. Given the smaller sample sizes and limited numbers of events in some strata, these findings should be interpreted cautiously.

Sensitivity analyses generally supported the robustness of the primary findings. In Fine–Gray competing‐risk models for cardiovascular mortality, with noncardiovascular death treated as a competing event, the directions of association were consistent with those observed in the primary Cox models: ln CALLY remained inversely associated with cardiovascular mortality, whereas ln RAR and ln BAR remained positively associated with cardiovascular mortality (Supporting Information S1: Table S3). Among participants from the NHANES 1999 to 2004 cycles with available NT‐proBNP measurements, additional adjustment for ln NT‐proBNP did not materially alter the observed associations (Supporting Information S1: Table S4). Analyses excluding deaths occurring within the first 2 years of follow‐up also yielded broadly consistent results (Supporting Information S1: Table S5). Similar findings were observed after multiple imputation for missing covariate data (Supporting Information S1: Table S6). To assess whether baseline medication use influenced the primary findings, we conducted a separate sensitivity analysis. Further adjustment for baseline antihypertensive medication use, glucose‐lowering medication use, and lipid‐lowering medication use did not materially alter the observed associations (Supporting Information S1: Table S7).

To assess the potential influence of temporal differences across survey cycles, we conducted three additional analyses. First, analyses restricted to participants enrolled in the more recent NHANES 2005–2010 cycles included 8077 participants, with 1535 all‐cause deaths and 483 cardiovascular deaths. The associations of ln CALLY, ln RAR, and ln BAR with all‐cause and cardiovascular mortality remained broadly consistent with the primary findings (Supporting Information S1: Table S15). Second, further adjustment for NHANES survey cycle did not materially alter the observed associations (Supporting Information S1: Table S16). Third, in an exploratory temporal validation analysis using the NHANES 2015–2018 cycles, in which CALLY was calculated using hs‐CRP rather than conventional CRP, the associations of ln CALLY, ln RAR, and ln BAR with all‐cause mortality were directionally consistent with the primary findings (Supporting Information S1: Table S17). Results for cardiovascular mortality should be interpreted cautiously because of the shorter follow‐up duration and the limited number of cardiovascular deaths in the contemporary cohort.

Exploratory Boruta feature selection and SHAP summary plots are presented in Supporting Information S1: Figures S7 and S8. Boruta analysis retained age, eGFR, and the three composite laboratory indices as relevant features in the models for both all‐cause and cardiovascular mortality, with ln RAR and ln BAR ranking higher than ln CALLY. In SHAP analyses, higher ln RAR and ln BAR generally contributed to higher predicted mortality risk, whereas higher ln CALLY contributed to lower predicted risk. These exploratory analyses were not used for primary statistical inference.

## 4 Discussion

The present study aimed to evaluate the associations of CALLY, RAR, and BAR with all‐cause and cardiovascular mortality among US adults with PHF and to explore whether these routinely available composite laboratory indices provide incremental prognostic information beyond conventional clinical factors. Using a nationally representative NHANES cohort with long‐term mortality follow‐up, survey‐weighted Cox regression models, nonlinear analyses, subgroup analyses, and multiple sensitivity analyses, we found that higher ln CALLY was associated with lower risks of all‐cause and cardiovascular mortality, whereas higher ln RAR and ln BAR were associated with higher risks of both outcomes. The overall directions of these associations remained broadly consistent across sensitivity analyses. Among the three indices, ln RAR showed the most consistent exploratory incremental prognostic value beyond the base model.

Previous studies have reported that inflammation‐ and nutrition‐related biomarkers are associated with adverse outcomes among patients with established HF. Elevated RAR has been associated with increased mortality among patients with HF [11, 12, 26], whereas elevated BAR has been linked to worse outcomes in patients with chronic or congestive HF [13, 14, 27]. In contrast, CALLY has been investigated mainly in oncologic populations [10, 15], and its prognostic relevance in adults with PHF has not been well characterized. The present study, therefore, extends previous evidence by evaluating three complementary composite indices at an earlier stage of the HF continuum in a population‐based setting.

HF is a complex clinical syndrome closely associated with chronic low‐grade systemic inflammation, which contributes to disease progression and adverse long‐term outcomes [28]. Inflammatory and nutrition‐related biomarkers, including CRP, serum albumin, lymphocyte count, and red cell distribution width, have increasingly been recognized as prognostically relevant markers in patients with HF [28, 29, 30]. Pay et al. reported that lower serum albumin levels were associated with increased long‐term all‐cause mortality among patients with HF receiving cardiac resynchronization therapy defibrillators [30]. Similarly, among patients with HFrEF receiving implantable cardioverter‐defibrillators, Çinier et al. identified blood urea nitrogen, pro‐BNP, and albumin, together with several clinical factors, as independent predictors of 1‐year mortality [31]. Hayıroğlu et al. further compared established mortality risk scores in elderly patients with HFrEF receiving implantable cardioverter‐defibrillators, highlighting the value of integrated multidimensional risk assessment for prognostic stratification [32].

Although these studies were conducted in patients with established HF rather than PHF, they provide a supportive biological context for evaluating composite indices at an earlier stage of the HF continuum. In individuals with PHF, inflammatory activation, nutritional impairment, immune dysfunction, hematologic abnormalities, and renal‐metabolic stress may already be present before clinically overt HF develops. These systemic processes may coexist with cardiometabolic and vascular risk factors and may identify individuals with reduced physiological reserve. Composite indices integrating these interrelated domains may therefore provide complementary prognostic information beyond isolated laboratory parameters.

CALLY integrates CRP, serum albumin, and lymphocyte count and may therefore reflect inflammatory burden, nutritional reserve, and immune function [10, 15]. CRP is an established marker of systemic inflammation [7, 33, 34], whereas lower albumin levels may reflect both inflammatory activation and impaired nutritional status [8, 30]. Reduced lymphocyte counts may indicate impaired immune homeostasis and have been associated with adverse outcomes among patients hospitalized with HF [29]. In the present study, higher ln CALLY was associated with lower mortality risk. The nonlinear findings further suggest that low CALLY levels may be particularly informative for identifying individuals with an unfavorable inflammatory–nutritional–immune profile, whereas the additional information provided by higher values may be more limited.

RAR may capture a different dimension of systemic vulnerability. Red cell distribution width reflects heterogeneity in erythrocyte size and may be influenced by chronic inflammation, oxidative stress, impaired erythropoiesis, abnormalities in iron metabolism, and comorbidity burden. When combined with albumin, RAR may integrate hematologic variability with inflammatory and nutritional reserve. This interpretation is consistent with previous studies showing associations between elevated RAR and adverse outcomes among patients with HF [11, 12, 26]. In the present study, ln RAR showed the most consistent exploratory incremental prognostic value beyond conventional clinical factors. One possible explanation is that RAR captures systemic vulnerability that is not fully reflected by traditional cardiovascular risk factors alone.

BAR incorporates blood urea nitrogen and albumin and may reflect the combined effects of renal‐metabolic stress and nutritional status. Blood urea nitrogen may be influenced by renal function, renal perfusion, neurohormonal activation, and catabolic burden, whereas albumin reflects inflammatory and nutritional reserve. Previous studies have associated elevated BAR with adverse outcomes among patients with HF [13, 14, 27]. Blood urea nitrogen and albumin have also been identified as independent predictors of 1‐year mortality among patients with HFrEF receiving implantable cardioverter‐defibrillators [31]. In the present study, higher ln BAR was associated with increased mortality risk, although its incremental prognostic performance was less consistent than that of ln RAR.

PHF is inherently heterogeneous because major cardiometabolic and vascular risk factors frequently coexist [5, 35, 36]. These components may contribute to adverse outcomes through partially overlapping but distinct pathways. Obesity and diabetes mellitus are closely linked to metabolic and inflammatory dysregulation, whereas hypertension and ASCVD may reflect a greater hemodynamic and vascular burden [28]. In the present study, the overall directions of association for CALLY and RAR were broadly consistent across different PHF compositions, suggesting that inflammatory, nutritional, immune, and hematologic disturbances may represent common dimensions of systemic vulnerability across the PHF spectrum. For ln BAR, no statistically significant interactions were identified in the PHF‐composition analyses, suggesting that the direction of association was broadly consistent across subgroups. These findings support the importance of considering PHF as a heterogeneous clinical state rather than a uniform category. Because some phenotype‐specific analyses included relatively few cardiovascular events, particularly among participants with isolated diabetes mellitus or ASCVD, the findings should be interpreted cautiously and require further validation.

The exploratory predictive analyses provide additional context for the potential clinical relevance of these indices. Among the three indices, RAR showed the most consistent incremental prognostic value beyond conventional clinical factors, particularly for all‐cause mortality prediction. Although the absolute increases in AUC and incremental net benefit were modest, RAR may provide additional risk‐stratification information when used as an adjunct to existing risk‐assessment strategies. This potential value is supported by its derivation from routinely available laboratory parameters and its ease of calculation.

However, statistical improvement should not be equated with established clinical actionability. RAR should therefore be considered a potentially informative adjunctive marker rather than a stand‐alone clinical decision tool. The present findings provide a rationale for further validation of RAR as a readily accessible marker for mortality risk stratification. Further studies in independent cohorts are warranted to assess its predictive robustness and to identify clinically relevant risk thresholds and potential application settings.

This study has several strengths. First, it used a large, nationally representative NHANES cohort of US adults with PHF and long‐term mortality follow‐up through National Death Index linkage. Second, the primary analyses accounted for the complex survey design and were complemented by nonlinear analyses, threshold analyses, subgroup analyses, phenotype‐specific sensitivity analyses, and exploratory incremental predictive analyses. Third, the robustness of the findings was evaluated using Fine–Gray competing‐risk models, additional adjustment for ln NT‐proBNP among participants with available measurements, exclusion of early deaths, multiple imputation for missing covariates, additional adjustment for baseline medication use, restriction to the more recent NHANES 2005–2010 cycles, further adjustment for NHANES survey cycle, and exploratory temporal validation using the NHANES 2015–2018 cycles.

Several limitations should be acknowledged. First, the observational design precludes causal inference, and residual confounding cannot be completely excluded despite multivariable adjustment. Although the findings remained materially unchanged after further adjustment for baseline antihypertensive medication use, glucose‐lowering medication use, and lipid‐lowering medication use, detailed information on medication dose, adherence, treatment duration, and changes in medication use during follow‐up was not fully captured. Other unmeasured or incompletely measured factors may also have influenced the observed associations. Second, CALLY, RAR, and BAR were calculated from single baseline laboratory measurements; therefore, longitudinal changes in inflammatory, nutritional, hematologic, and renal‐metabolic status could not be assessed. Third, PHF was identified using a population‐based operational definition based on variables available in NHANES and consistent with previously published NHANES criteria [5]. Although hypertension, diabetes mellitus, and obesity were defined using measured clinical or laboratory data and medication use, some components, including ASCVD, history of myocardial infarction, and physician‐diagnosed HF, relied partly on self‐reported information and may therefore be subject to misclassification. Echocardiographic measurements were not available across the included NHANES cycles. Consequently, we were unable to characterize left ventricular structure, systolic or diastolic dysfunction, or other imaging‐based features of PHF. Although further adjustment for ln NT‐proBNP among participants with available measurements did not materially alter the observed associations, this sensitivity analysis does not replace echocardiographic assessment. The study population should therefore be interpreted as PHF identified using an epidemiological operational definition rather than as an echocardiographically confirmed PHF cohort. Fourth, NHANES‐linked mortality data do not provide longitudinal information on incident HF; consequently, this study evaluated mortality among adults with PHF rather than progression from PHF to clinically diagnosed HF. Fifth, although analyses restricted to more recent survey cycles and exploratory temporal validation yielded broadly consistent findings, temporal changes in population characteristics and clinical management cannot be fully excluded. Further validation in contemporary cohorts with longer follow‐up remains warranted. Sixth, although subgroup and phenotype‐specific analyses generally supported the consistency of the findings across different PHF compositions, some strata had smaller sample sizes and limited numbers of events. Moreover, these analyses were conducted within the same NHANES cohort and do not replace external validation. Because NHANES is designed to represent the US civilian, noninstitutionalized population, the generalizability of the findings to populations in other countries, institutionalized individuals, hospitalized patients, patients with established HF, and other specific clinical settings remains uncertain. Finally, the predictive performance, Boruta, and SHAP analyses were exploratory and should not be interpreted as confirmatory evidence or as substitutes for external validation and survival‐model‐based inference. Although ln RAR showed the most consistent incremental predictive performance and net benefit for all‐cause mortality prediction among the three indices, the absolute improvements were modest. Moreover, the incremental net benefit for cardiovascular mortality varied across prediction horizons and risk thresholds. Therefore, RAR should currently be interpreted as a potentially informative adjunctive marker rather than as a stand‐alone clinical decision tool. Further validation is required to clarify its potential role in clinical risk‐stratification strategies.

## 5 Conclusion

CALLY, RAR, and BAR were independently associated with long‐term all‐cause and cardiovascular mortality among adults with PHF. Higher ln CALLY was associated with lower mortality risk, whereas higher ln RAR and ln BAR were associated with higher mortality risk. Among the three indices, ln RAR showed the most consistent exploratory incremental prognostic value and a modest net benefit, particularly for all‐cause mortality prediction. These routinely available indices, especially RAR, may serve as adjunctive markers for mortality risk stratification in adults with PHF. Further validation in independent cohorts is warranted before their incorporation into clinical risk‐assessment strategies.

## Author Contributions

H.S. was responsible for the conception and design of the study, conducted the formal statistical analyses, developed the methodology, created the original draft of the manuscript, developed and maintained the software used in the analyses, and provided overall supervision of the project. X.L. curated and managed the data, performed the investigation, generated all visualizations, and critically reviewed and edited the manuscript.

## Funding

The authors have nothing to report.

## Ethics Statement

Study protocols for NHANES were approved by the National Center for Health Statistics Ethics Review Board (Protocol #2011‐17, https://www.cdc.gov/nchs/nhanes/irba98.htm). All the participants signed the informed consent before participating in the study. The NHANES protocols were approved by the NCHS Ethics Review Board, and all participants provided written informed consent. The present analysis was conducted in accordance with the Declaration of Helsinki.

## Consent

The authors have nothing to report.

## Conflicts of Interest

The authors declare no conflicts of interest.

## Supporting information

Supporting File

## Data Availability

These survey data are free and publicly available, and can be downloaded directly from the NHANES website (http://www.cdc.gov/nchs/nhanes/) by users and researchers worldwide. The NHANES data and public‐use linked mortality files used in this study are publicly available from the National Center for Health Statistics. The analytic code is available from the corresponding author upon reasonable request.
