# Supplementary material for: Associations of the C‐Reactive Protein−Albumin−Lymphocyte Index, Red Cell Distribution Width−Albumin Ratio, and Blood Urea Nitrogen−Albumin Ratio With All‐Cause and Cardiovascular Mortality Among Adults With Preclinical Heart Failure
Source: Clin Cardiol. 2026 Jul 6;49(7):e70401. doi: 10.1002/clc.70401 (PMC13334882; doi:10.1002/clc.70401)
Supplement: Supplementary file 1 — Supporting File [file CLC-49-e70401-s001.docx]

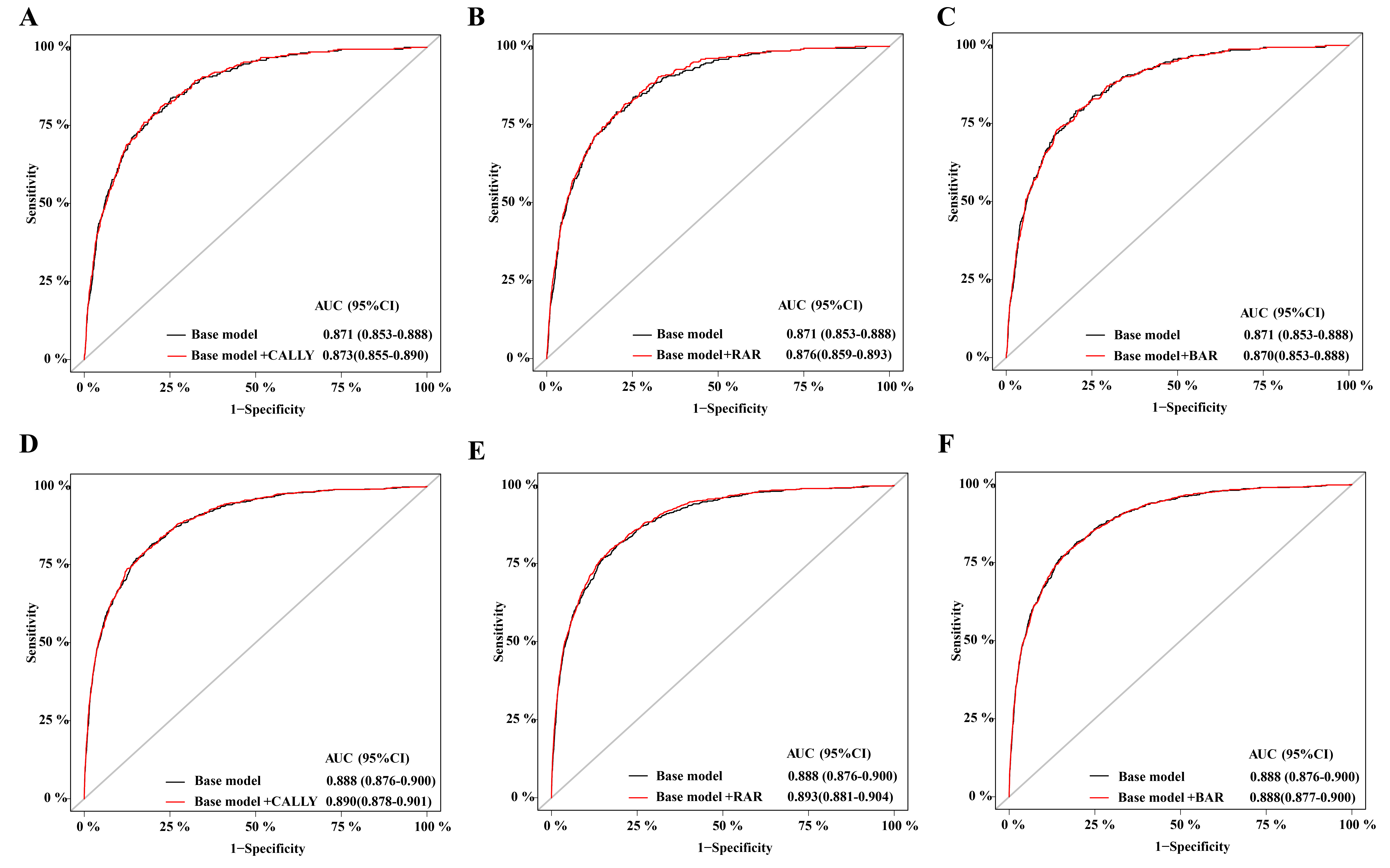


Figure S1 Exploratory time-dependent receiver operating characteristic curves for cardiovascular mortality prediction

Panels A-C compare the base model with models separately adding ln CALLY, ln RAR, or ln BAR at 60 months; panels D-F show the corresponding comparisons at 120 months. Areas under the curve (AUCs) with 95% confidence intervals (CIs) are displayed in each panel.


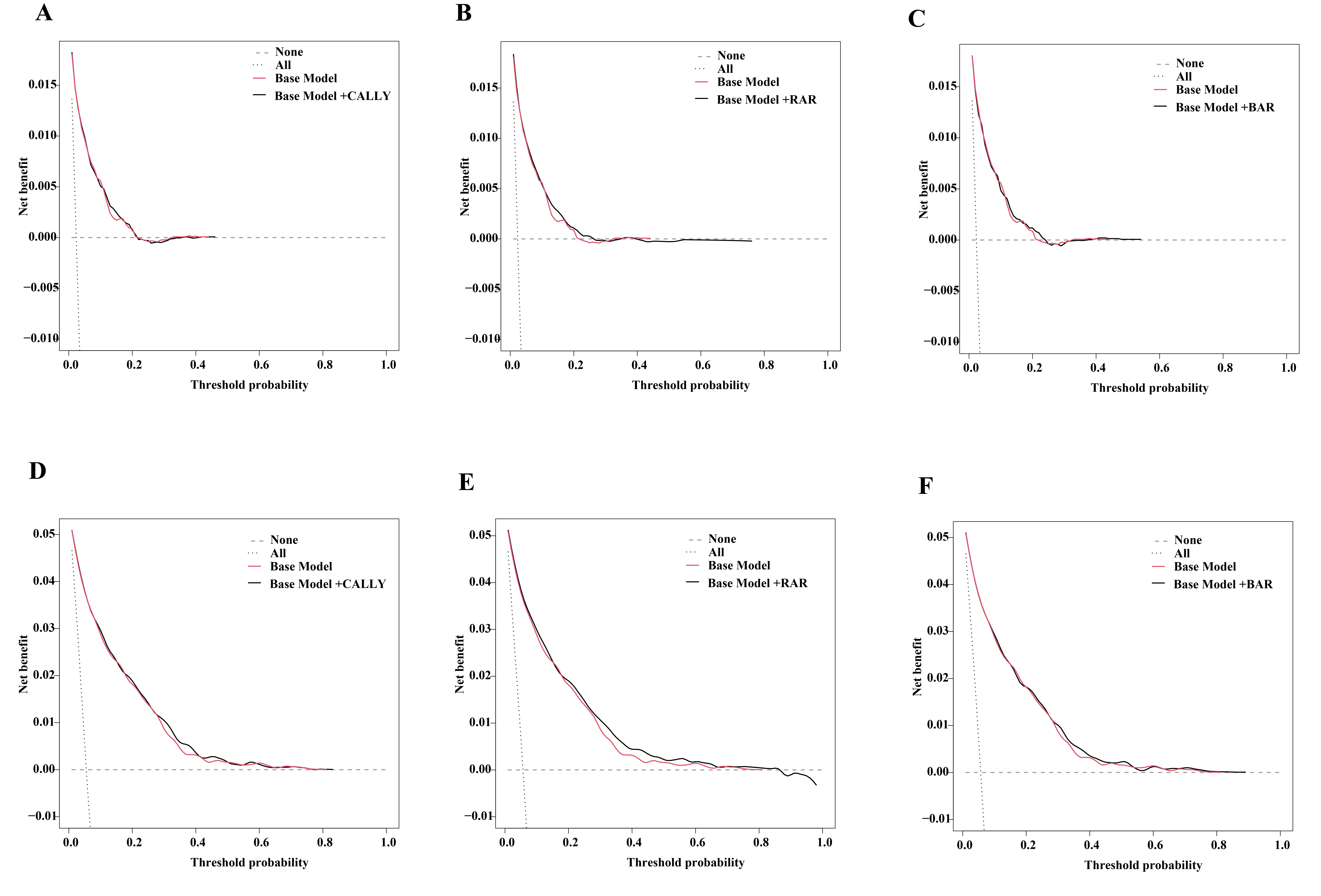


Figure S2 Exploratory decision curve analyses for cardiovascular mortality prediction

Panels A-C compare the base model with models separately adding ln CALLY, ln RAR, or ln BAR at 60 months; panels D-F show the corresponding comparisons at 120 months. Net benefit is plotted across threshold probabilities.


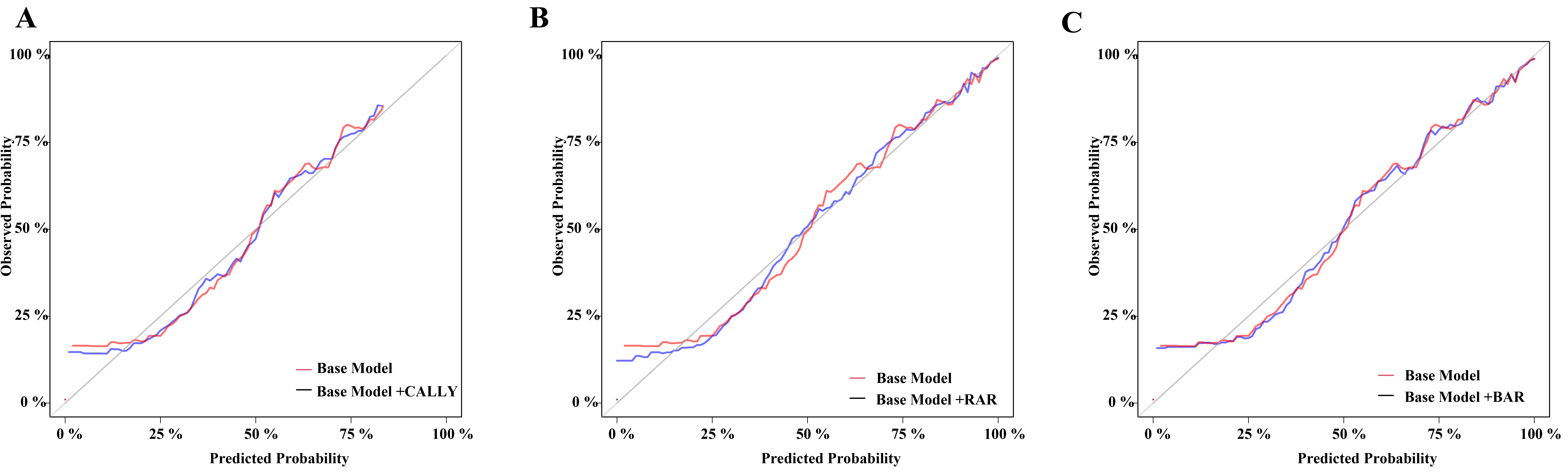
Figure S3 Exploratory calibration curves for 10-year all-cause mortality prediction

Panels A-C compare the base model with models separately adding ln CALLY, ln RAR, or ln BAR. The gray diagonal line represents perfect calibration.


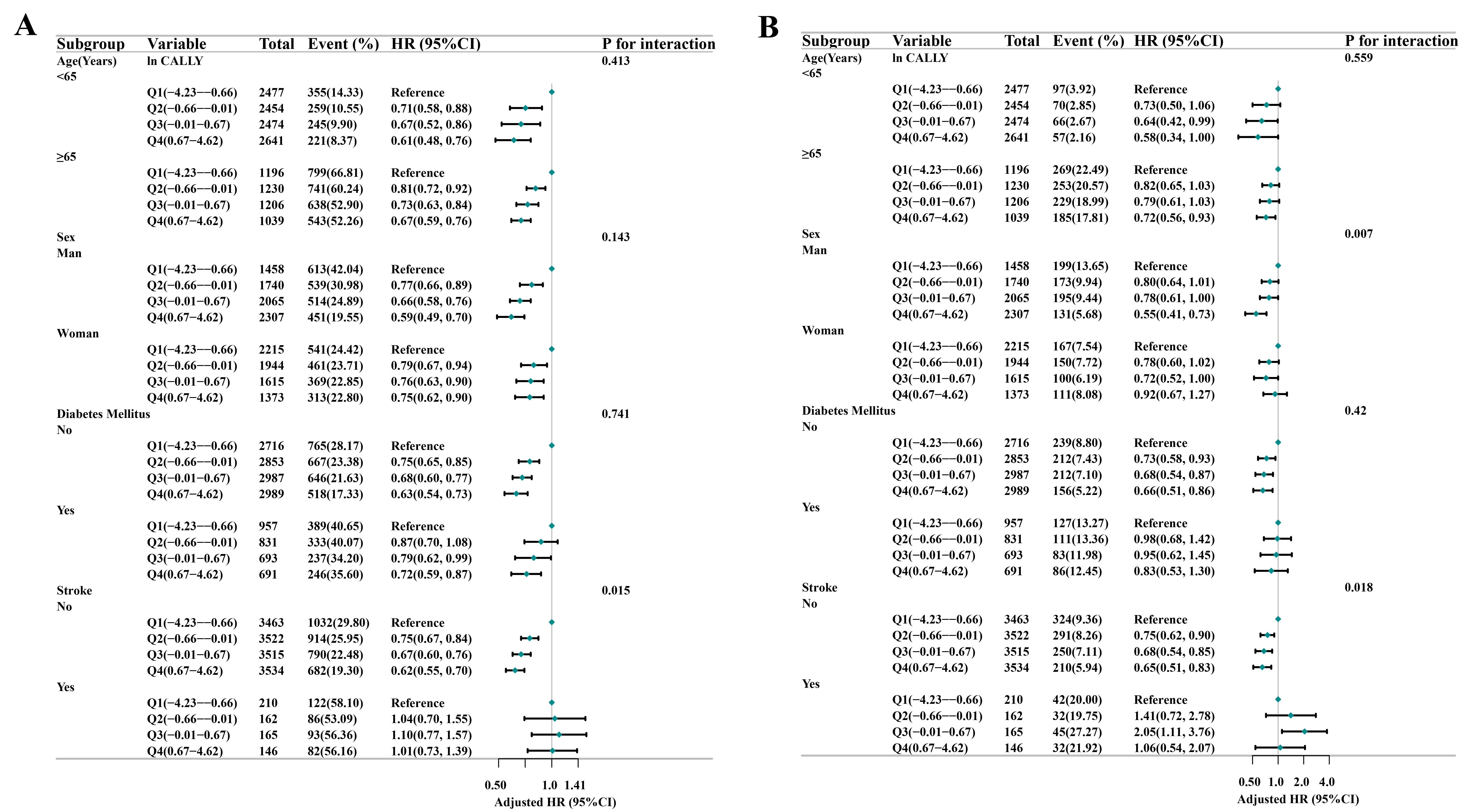


Figure S4. Prespecified subgroup analyses of the associations of ln CALLY with all-cause and cardiovascular mortality

A, all-cause mortality; B, cardiovascular mortality. Hazard ratios (HRs) and 95% confidence intervals (CIs) are shown across prespecified subgroups, with P values for interaction provided for each subgroup factor.


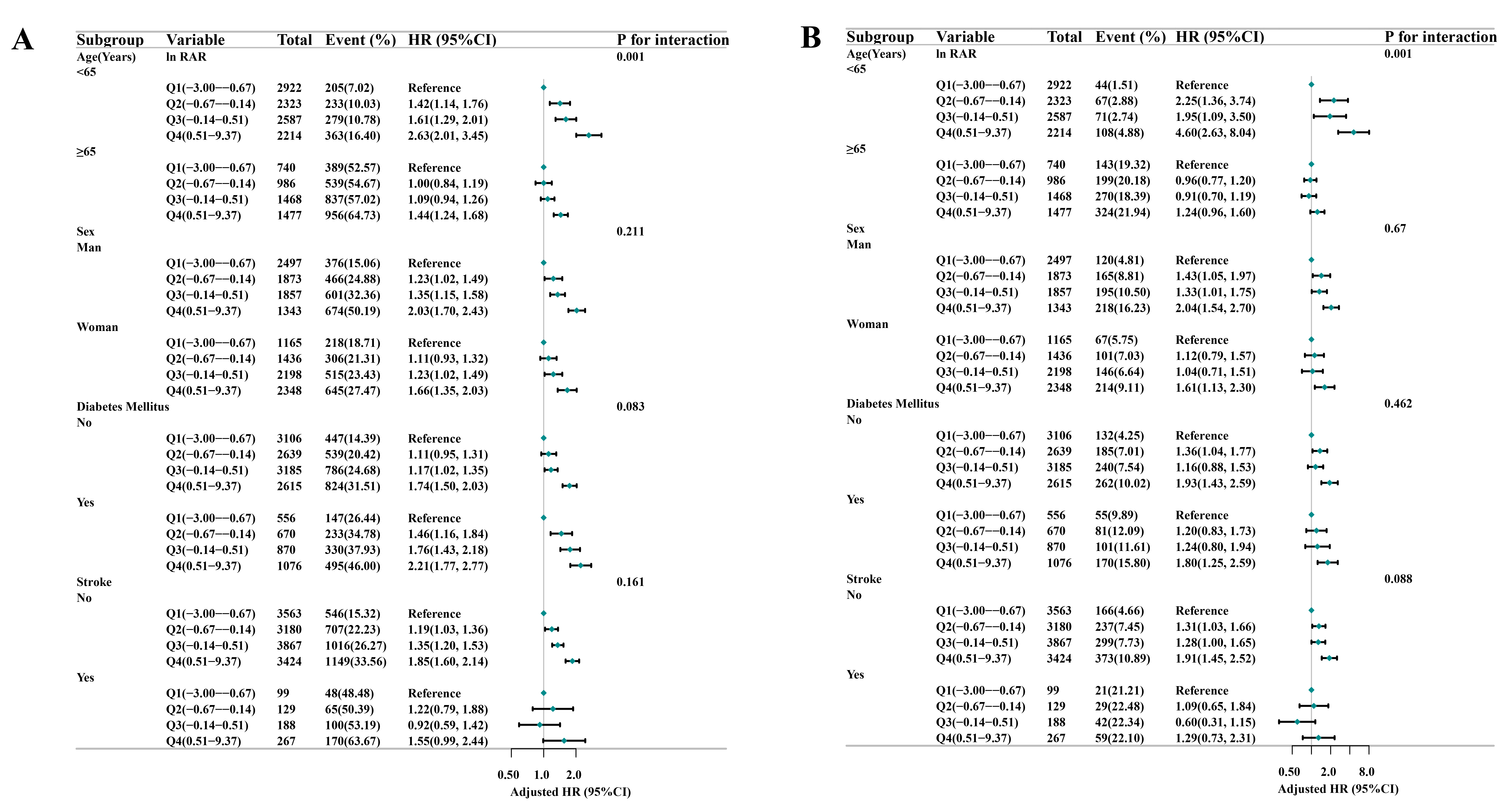


Figure S5 Prespecified subgroup analyses of the associations of ln RAR with all-cause and cardiovascular mortality

A, all-cause mortality; B, cardiovascular mortality. Hazard ratios (HRs) and 95% confidence intervals (CIs) are shown across prespecified subgroups, with P values for interaction provided for each subgroup factor.


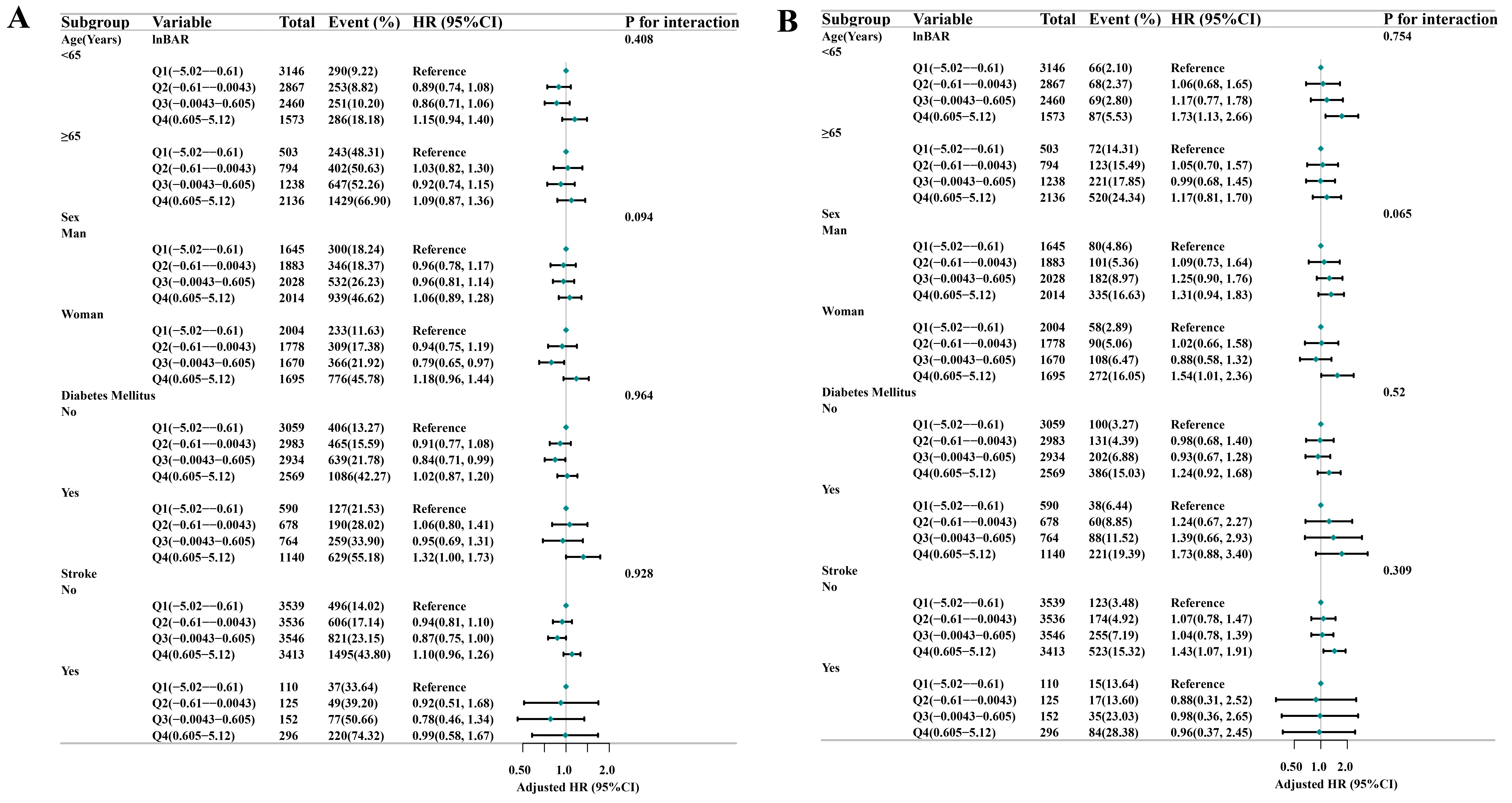


Figure S6 Prespecified subgroup analyses of the associations of ln BAR with all-cause and cardiovascular mortality

A, all-cause mortality; B, cardiovascular mortality. Hazard ratios (HRs) and 95% confidence intervals (CIs) are shown across prespecified subgroups, with P values for interaction provided for each subgroup factor.


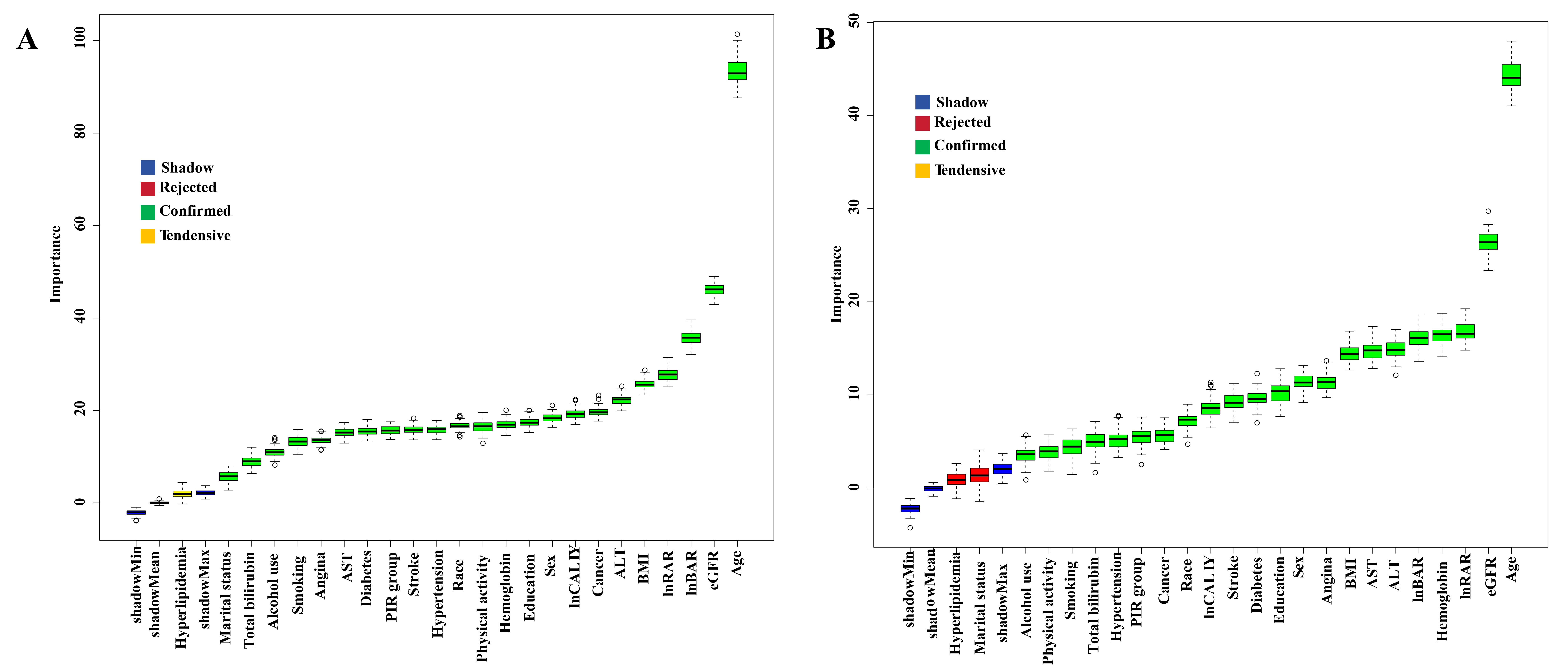


Figure S7. Exploratory Boruta feature selection for mortality outcomes

Panels A and B show feature-importance distributions for all-cause and cardiovascular mortality, respectively. Green boxes indicate confirmed features, red boxes indicate rejected features, blue boxes indicate shadow features, and yellow boxes indicate tentative features.


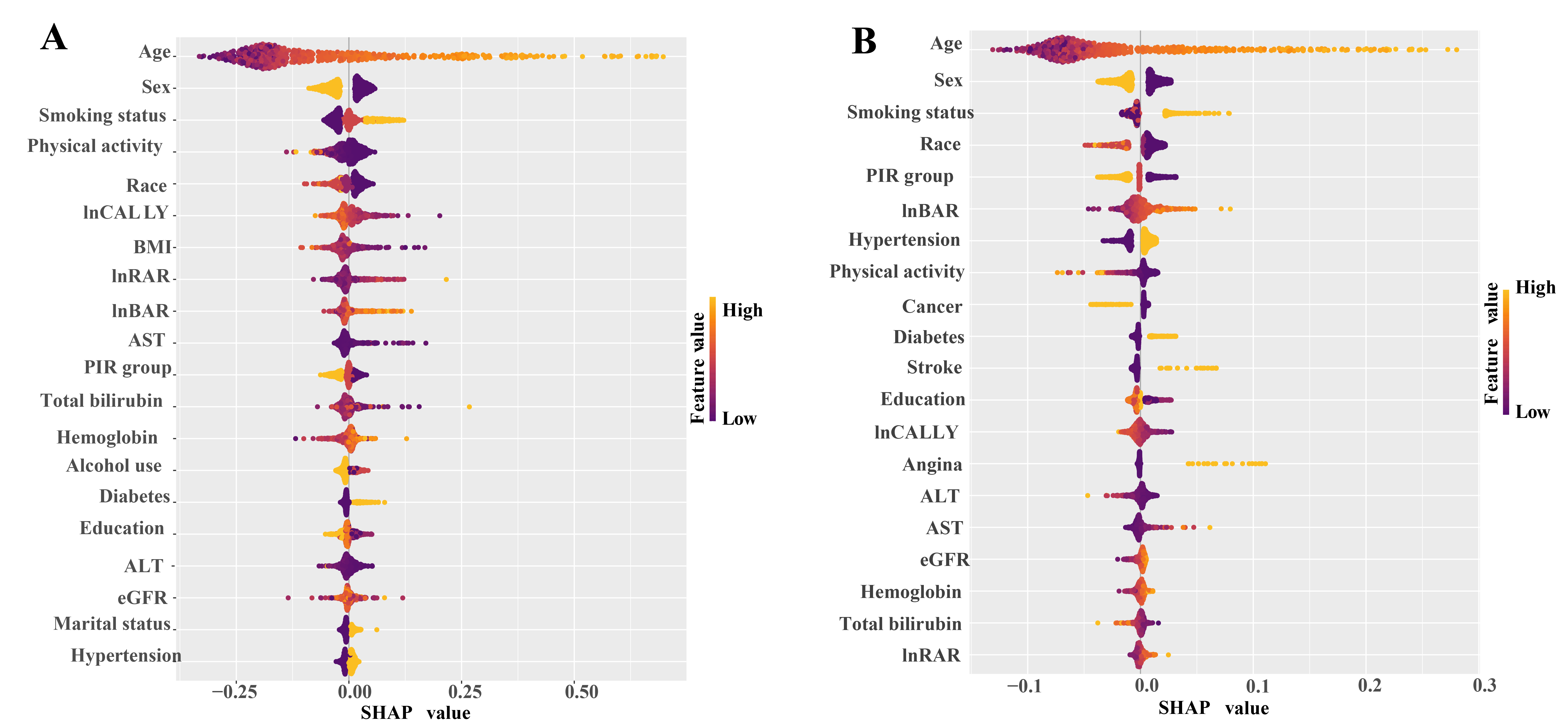


Figure S8. Exploratory SHAP summary plots for mortality outcomes

Panels A and B show SHAP summary plots for all-cause and cardiovascular mortality, respectively. Each point represents an individual participant; color indicates the feature value, and horizontal position indicates the SHAP value. These analyses were not used for primary statistical


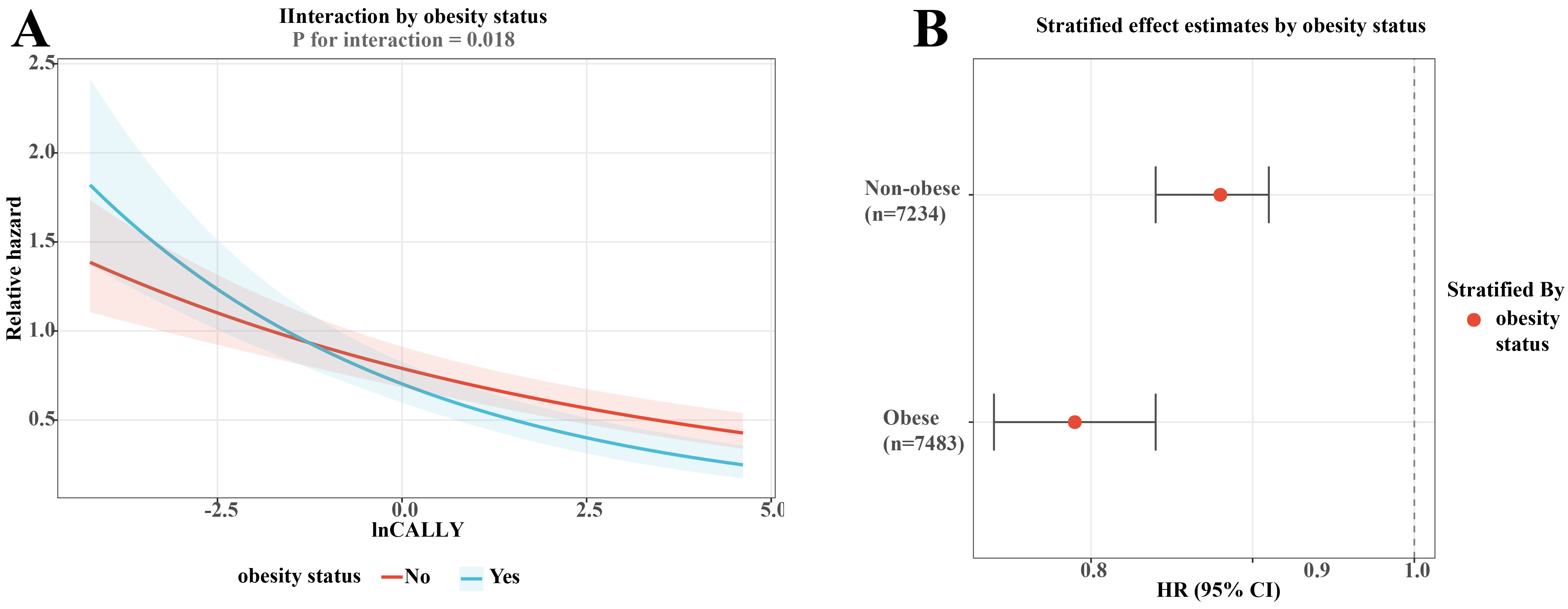


**Supplementary Figure S9. Interaction between ln CALLY and obesity status for all-cause mortality among adults with preclinical heart failure.**

(A) Adjusted fitted curves showing the association between standardized ln CALLY and the relative hazard of all-cause mortality, stratified by obesity status. Solid lines represent the estimated relative hazards, and shaded areas indicate the corresponding 95% confidence intervals. (B) Stratified effect estimates for the association of each 1-standard deviation increase in ln CALLY with all-cause mortality according to obesity status. Dots represent hazard ratios, and horizontal lines indicate 95% confidence intervals. The vertical dashed line denotes a hazard ratio of 1.00. The P value for interaction was calculated using the likelihood ratio test. Obesity was defined as a body mass index of ≥30 kg/m².

**Abbreviations:** CALLY, C-reactive protein–albumin–lymphocyte index; CI, confidence interval; HR, hazard ratio; PHF, preclinical heart failure; SD, standard deviation.


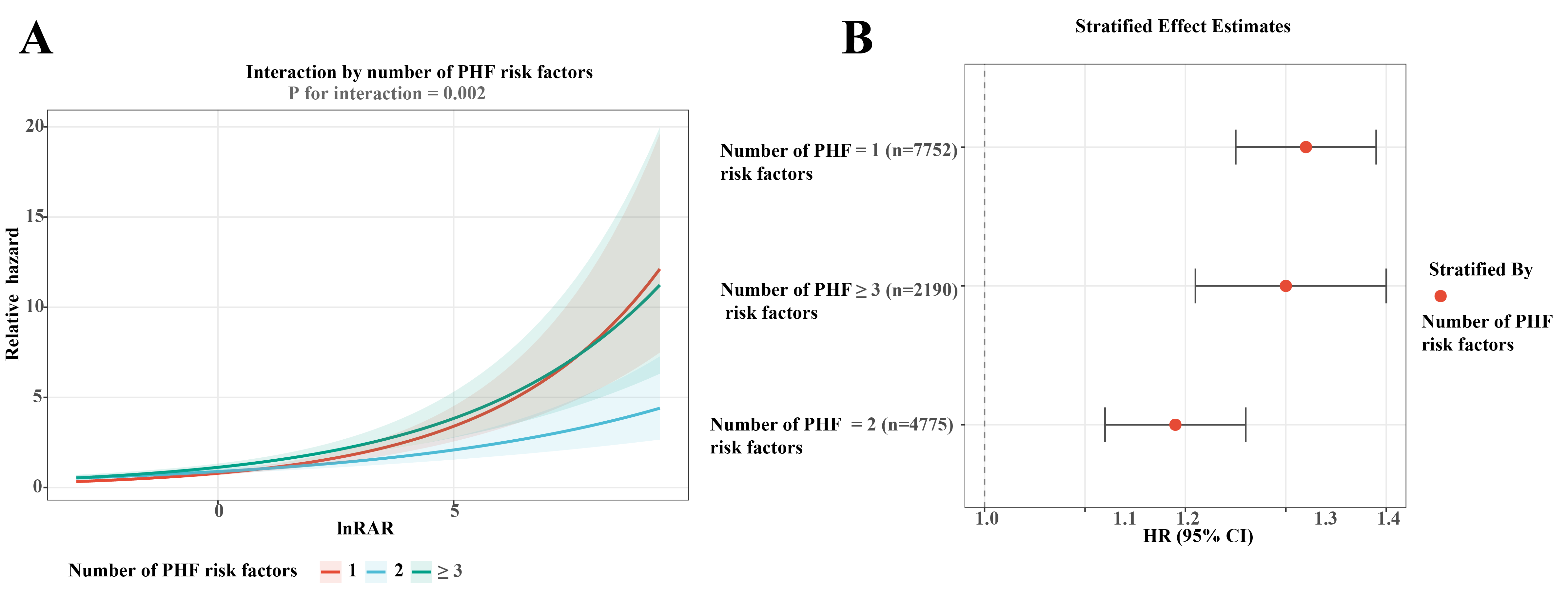


**Supplementary Figure S10. Interaction between ln RAR and the number of PHF components for all-cause mortality among adults with preclinical heart failure.**

(A) Adjusted fitted curves showing the association between standardized ln RAR and the relative hazard of all-cause mortality, stratified by the number of PHF components. Solid lines represent the estimated relative hazards, and shaded areas indicate the corresponding 95% confidence intervals. (B) Stratified effect estimates for the association of each 1-standard deviation increase in ln RAR with all-cause mortality according to the number of PHF components. Dots represent hazard ratios, and horizontal lines indicate 95% confidence intervals. The vertical dashed line denotes a hazard ratio of 1.00. The P value for interaction was calculated using the likelihood ratio test. The number of PHF components was categorized as 1, 2, or ≥3.

**Abbreviations:** CI, confidence interval; HR, hazard ratio; PHF, preclinical heart failure; RAR, red cell distribution width–albumin ratio; SD, standard deviation.


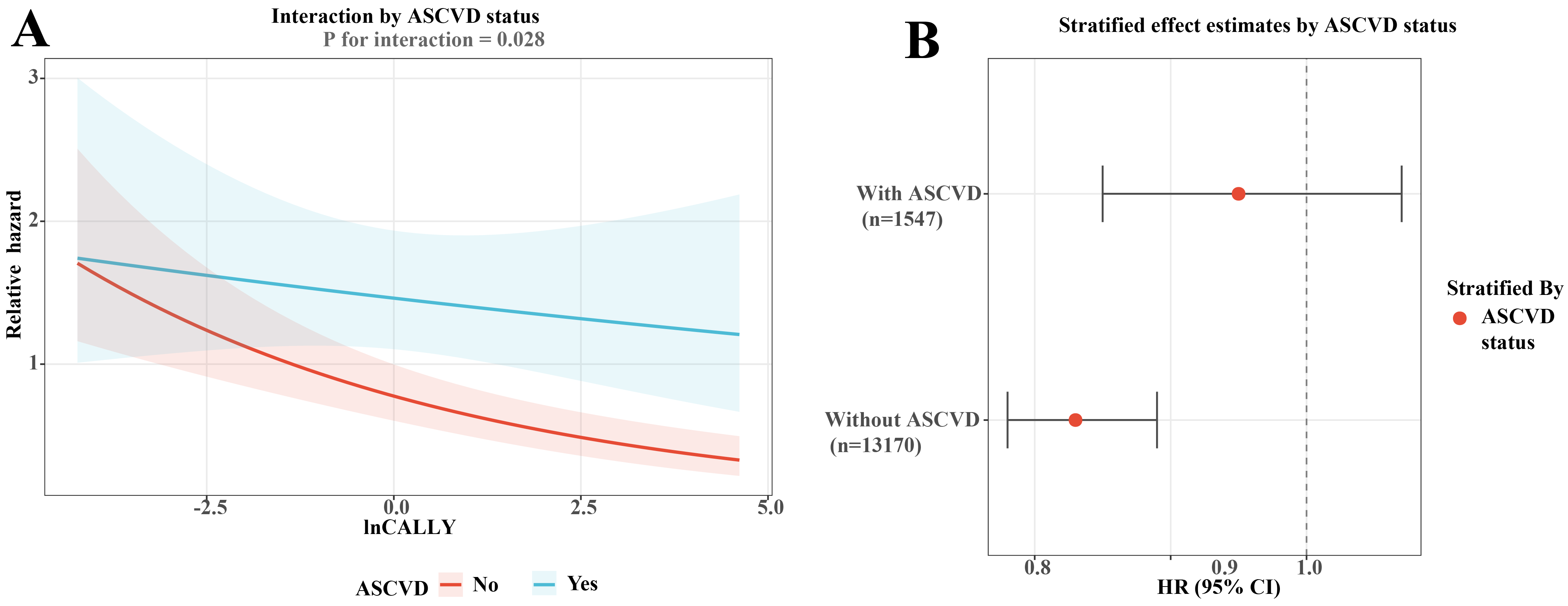


**Supplementary Figure S11. Interaction between ln CALLY and ASCVD status for cardiovascular mortality among adults with preclinical heart failure**

(A) Adjusted fitted curves showing the association between standardized ln CALLY and the relative hazard of cardiovascular mortality, stratified by ASCVD status. Solid lines represent the estimated relative hazards, and shaded areas indicate the corresponding 95% confidence intervals. (B) Stratified effect estimates for the association of each 1-standard deviation increase in ln CALLY with cardiovascular mortality according to ASCVD status. Dots represent hazard ratios, and horizontal lines indicate 95% confidence intervals. The vertical dashed line denotes a hazard ratio of 1.00. The P value for interaction was calculated using the likelihood ratio test.

**Abbreviations:** ASCVD, atherosclerotic cardiovascular disease; CALLY, C-reactive protein–albumin–lymphocyte index; CI, confidence interval; HR, hazard ratio; PHF, preclinical heart failure; SD, standard deviation.


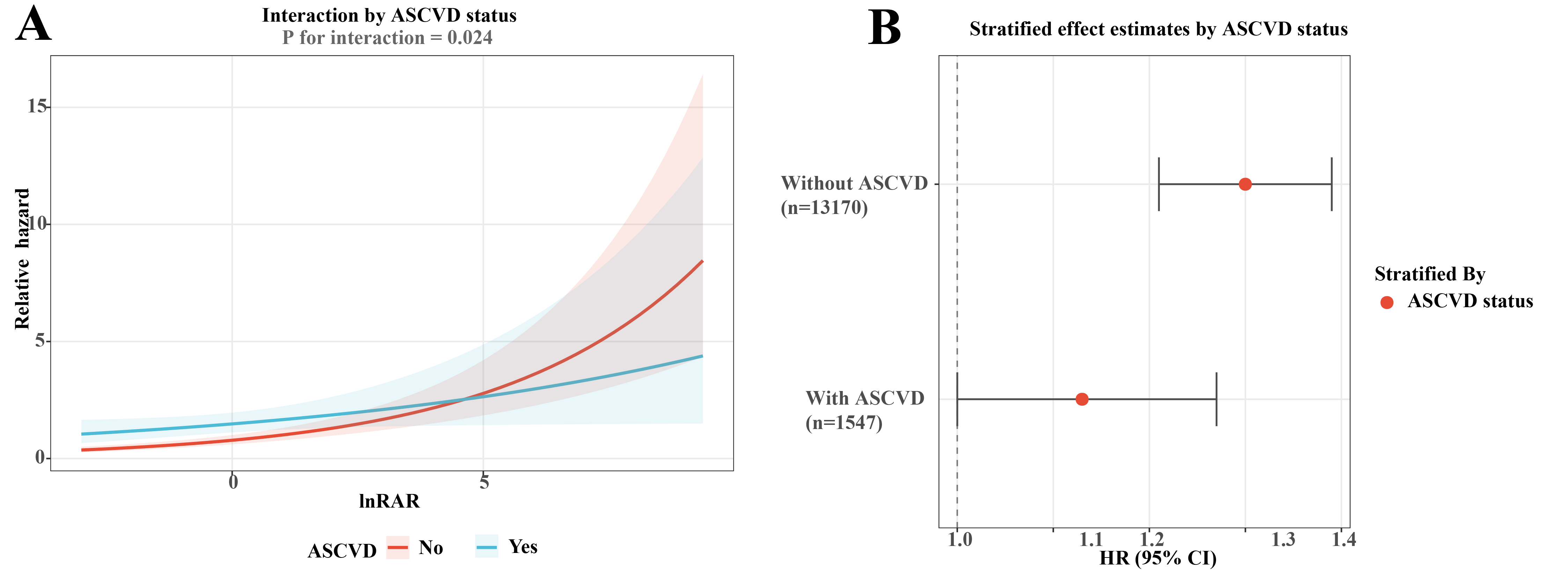
**Supplementary Figure S12. Interaction between ln RAR and ASCVD status for cardiovascular mortality among adults with preclinical heart failure**

(A) Adjusted fitted curves showing the association between standardized ln RAR and the relative hazard of cardiovascular mortality, stratified by ASCVD status. Solid lines represent the estimated relative hazards, and shaded areas indicate the corresponding 95% confidence intervals. (B) Stratified effect estimates for the association of each 1-standard deviation increase in ln RAR with cardiovascular mortality according to ASCVD status. Dots represent hazard ratios, and horizontal lines indicate 95% confidence intervals. The vertical dashed line denotes a hazard ratio of 1.00. The P value for interaction was calculated using the likelihood ratio test.

**Abbreviations:** ASCVD, atherosclerotic cardiovascular disease; CI, confidence interval; HR, hazard ratio; PHF, preclinical heart failure; RAR, red cell distribution width–albumin ratio; SD, standard deviation.

**Table S1** Comparison of baseline characteristics between participants included in the primary complete-case analysis and those excluded because of missing covariate data

| Variables | Total (n = 17,321) | Included participants (n = 14,717) | Excluded participants (n = 2,604) | P value |
| --- | --- | --- | --- | --- |
| Age, years, mean ± SD | 54.4 ± 17.3 | 54.3 ± 17.3 | 54.5 ± 17.8 | 0.596 |
| Sex, n (%) |  |  |  | < 0.001 |
| Male | 8774 (50.7) | 7570 (51.4) | 1204 (46.2) |  |
| Female | 8547 (49.3) | 7147 (48.6) | 1400 (53.8) |  |
| Race/ethnicity, n (%) |  |  |  | < 0.001 |
| Non-Hispanic White | 8560 (49.4) | 7492 (50.9) | 1068 (41) |  |
| Non-Hispanic Black | 3679 (21.2) | 3070 (20.9) | 609 (23.4) |  |
| Mexican American | 3386 (19.5) | 2810 (19.1) | 576 (22.1) |  |
| Other Hispanic | 1077 ( 6.2) | 860 (5.8) | 217 (8.3) |  |
| Other race^a^ | 619 ( 3.6) | 485 (3.3) | 134 (5.1) |  |
| Marital status, n (%) |  |  |  | < 0.001 |
| Living with partner | 10534 (61.8) | 9181 (62.4) | 1353 (57.9) |  |
| Separated/divorced/widowed/never married | 6521 (38.2) | 5536 (37.6) | 985 (42.1) |  |
| Poverty income ratio, n (%) |  |  |  | < 0.001 |
| ≤1.30 | 4657 (29.2) | 4228 (28.7) | 429 (34.7) |  |
| 1.31–3.50 | 6265 (39.3) | 5777 (39.3) | 488 (39.4) |  |
| >3.50 | 5033 (31.5) | 4712 (32) | 321 (25.9) |  |
| Education level, n (%) |  |  |  | < 0.001 |
| Less than 9th grade | 2633 (15.2) | 2101 (14.3) | 532 (20.6) |  |
| 9–11th grade | 2885 (16.7) | 2428 (16.5) | 457 (17.7) |  |
| High school graduate/GED or equivalent | 4269 (24.7) | 3642 (24.7) | 627 (24.3) |  |
| Some college or AA degree | 4527 (26.2) | 3903 (26.5) | 624 (24.2) |  |
| College graduate or above | 2986 (17.3) | 2643 (18) | 343 (13.3) |  |
| Smoking status, n (%) |  |  |  | < 0.001 |
| Never | 8781 (50.7) | 7393 (50.2) | 1388 (53.6) |  |
| Former | 5000 (28.9) | 4335 (29.5) | 665 (25.7) |  |
| Current | 3527 (20.4) | 2989 (20.3) | 538 (20.8) |  |
| Alcohol use, n (%) |  |  |  | 0.006 |
| Never | 2392 (14.7) | 2127 (14.5) | 265 (17.5) |  |
| Former | 3698 (22.8) | 3359 (22.8) | 339 (22.4) |  |
| Current | 10142 (62.5) | 9231 (62.7) | 911 (60.1) |  |
| Physical activity, n (%) |  |  |  | 0.077 |
| Inactive | 10613 (61.3) | 8977 (61) | 1636 (62.8) |  |
| Active | 6708 (38.7) | 5740 (39) | 968 (37.2) |  |
| Body mass index, kg/m², mean ± SD | 30.6 ± 6.7 | 30.6 ± 6.7 | 30.3 ± 6.6 | 0.043 |
| Angina, n (%) |  |  |  | 0.47 |
| No | 16714 (96.5) | 14195 (96.5) | 2519 (96.7) |  |
| Yes | 607 ( 3.5) | 522 (3.5) | 85 (3.3) |  |
| Stroke, n (%) |  |  |  | 0.874 |
| No | 16519 (95.4) | 14034 (95.4) | 2485 (95.4) |  |
| Yes | 802 ( 4.6) | 683 (4.6) | 119 (4.6) |  |
| Hyperlipidemia, n (%) |  |  |  | 0.142 |
| No | 3429 (19.8) | 2886 (19.6) | 543 (20.9) |  |
| Yes | 13892 (80.2) | 11831 (80.4) | 2061 (79.1) |  |
| Hypertension, n (%) |  |  |  | 0.002 |
| No | 6972 (40.3) | 5851 (39.8) | 1121 (43) |  |
| Yes | 10349 (59.7) | 8866 (60.2) | 1483 (57) |  |
| Diabetes mellitus, n (%) |  |  |  | 0.386 |
| No | 13568 (78.3) | 11545 (78.4) | 2023 (77.7) |  |
| Yes | 3753 (21.7) | 3172 (21.6) | 581 (22.3) |  |
| Cancer , n (%) |  |  |  | 0.01 |
| No | 15484 (89.5) | 13135 (89.3) | 2349 (90.9) |  |
| Yes | 1816 (10.5) | 1582 (10.7) | 234 (9.1) |  |
| eGFR, mL/min/1.73 m², mean ± SD | 88.8 ± 23.3 | 88.6 ± 23.1 | 89.4 ± 24.7 | 0.112 |
| ALT, median (IQR) | 22.0 (17.0, 30.0) | 22.0 (17.0, 30.0) | 22.0 (17.0, 29.0) | 0.012 |
| AST, mean ± SD | 26.3 ± 16.2 | 26.3 ± 16.7 | 26.1 ± 13.6 | 0.544 |
| Hemoglobin, g/dL, mean ± SD | 14.3 ± 1.5 | 14.3 ± 1.5 | 14.1 ± 1.6 | < 0.001 |
| Total bilirubin, mg/dL, mean ± SD | 0.7 ± 0.3 | 0.7 ± 0.3 | 0.7 ± 0.3 | < 0.001 |
| ln CALLY, Mean ± SD | 3.5 ± 1.2 | 3.6 ± 1.3 | 3.5 ± 1.2 | 0.017 |
| ln RAR, Mean ± SD | 1.1 ± 0.1 | 1.1 ± 0.1 | 1.1 ± 0.1 | 0.786 |
| ln BAR, Mean ± SD | 1.1 ± 0.4 | 1.1 ± 0.4 | 1.1 ± 0.4 | 0.312 |

a Included multiracial participants; NHANES did not provide detailed racial/ethnic categories for this group.

Note: Values are unweighted and presented as mean ± SD, median (IQR), or n (%), as appropriate. P values compare included and excluded participants.

**Table S2** Exploratory incremental clinical net benefit of adding ln CALLY, ln RAR, or ln BAR to the base model for predicting all-cause and cardiovascular mortality at 60 and 120 months

| Prediction time | Outcome | Risk threshold | Model | Net benefit | ΔNB vs base model | Additional TP per 100 patients |
| --- | --- | --- | --- | --- | --- | --- |
| 60 months | All-cause mortality | 10% | Base model | 0.02865 | Ref | Ref |
|  |  |  | Base model + ln CALLY | 0.02933 | 0.00068 | 0.068 |
|  |  |  | Base model + ln RAR | 0.02985 | 0.00120 | 0.120 |
|  |  |  | Base model + ln BAR | 0.02867 | 0.00002 | 0.002 |
|  |  | 20% | Base model | 0.01396 | Ref | Ref |
|  |  |  | Base model + ln CALLY | 0.01498 | 0.00102 | 0.102 |
|  |  |  | Base model + ln RAR | 0.01539 | 0.00143 | 0.143 |
|  |  |  | Base model + ln BAR | 0.01400 | 0.00003 | 0.003 |
|  | Cardiovascular mortality | 10% | Base model | 0.00448 | Ref | Ref |
|  |  |  | Base model + ln CALLY | 0.00523 | 0.00075 | 0.075 |
|  |  |  | Base model + ln RAR | 0.00468 | 0.00021 | 0.021 |
|  |  |  | Base model + ln BAR | 0.00470 | 0.00022 | 0.022 |
|  |  | 20% | Base model | 0.00040 | Ref | Ref |
|  |  |  | Base model + ln CALLY | 0.00050 | 0.00011 | 0.011 |
|  |  |  | Base model + ln RAR | 0.00082 | 0.00042 | 0.042 |
|  |  |  | Base model + ln BAR | 0.00028 | -0.00012 | -0.012 |
| 120 months | All-cause mortality | 10% | Base model | 0.11267 | Ref | Ref |
|  |  |  | Base model + ln CALLY | 0.11301 | 0.00034 | 0.034 |
|  |  |  | Base model + ln RAR | 0.11479 | 0.00212 | 0.212 |
|  |  |  | Base model + ln BAR | 0.11184 | -0.00083 | -0.083 |
|  |  | 20% | Base model | 0.08302 | Ref | Ref |
|  |  |  | Base model + ln CALLY | 0.08372 | 0.00070 | 0.070 |
|  |  |  | Base model + ln RAR | 0.08657 | 0.00356 | 0.356 |
|  |  |  | Base model + ln BAR | 0.08323 | 0.00022 | 0.022 |
|  | Cardiovascular mortality | 10% | Base model | 0.02862 | Ref | Ref |
|  |  |  | Base model + ln CALLY | 0.02906 | 0.00044 | 0.044 |
|  |  |  | Base model + ln RAR | 0.03004 | 0.00142 | 0.142 |
|  |  |  | Base model + ln BAR | 0.02890 | 0.00028 | 0.028 |
|  |  | 20% | Base model | 0.01858 | Ref | Ref |
|  |  |  | Base model + ln CALLY | 0.01858 | 0.00000 | 0.000 |
|  |  |  | Base model + ln RAR | 0.01709 | -0.00149 | -0.149 |
|  |  |  | Base model + ln BAR | 0.01872 | 0.00014 | 0.014 |

Note: Each log-transformed index was added separately to the prespecified base model. Net benefit was estimated using decision curve analysis at risk thresholds of 10% and 20%. ΔNB was calculated relative to the base model, and additional true positives per 100 patients were calculated as ΔNB × 100. The 60- and 120-month horizons correspond to 5- and 10-year prediction, respectively. These analyses were exploratory.

Abbreviations: BAR, blood urea nitrogen-albumin ratio; CALLY, C-reactive protein-albumin-lymphocyte index; NB, net benefit; RAR, red cell distribution width-albumin ratio; Ref, reference; TP, true positives.

**Table S3** Fine-Gray competing-risk analyses of the associations of ln CALLY, ln RAR, and ln BAR with cardiovascular mortality

| Characteristics | Participants, n | Events, n (%) | Person-years | Model 1 | | Model 2 | | Model 3 | | Model 4 | |
| --- | --- | --- | --- | --- | --- | --- | --- | --- | --- | --- | --- |
|  |  |  |  | SHR(95% CI) | P value | SHR(95% CI) | P value | SHR(95% CI) | P value | SHR(95% CI) | P value |
| Competing-risk analysis for cardiovascular mortality | | | | | | | | | | | |
| ln CALLY (per SD) | 14717 | 1226 (8.3) | 186,678.5 | 0.85 (0.8–0.89) | <0.001 | 0.91 (0.86–0.97) | 0.003 | 0.92 (0.87–0.98) | 0.01 | 0.94 (0.88–1) | 0.048 |
| Q1 | 3673 | 366 (10) | 44,833.7 | 1.00 |  | 1.00 |  | 1.00 |  | 1.00 |  |
| Q2 | 3684 | 323 (8.8) | 46,993.7 | 0.87 (0.75–1.01) | 0.06 | 0.89 (0.76–1.04) | 0.139 | 0.89 (0.77–1.04) | 0.159 | 0.91 (0.78–1.06) | 0.226 |
| Q3 | 3680 | 295 (8) | 47,098.4 | 0.8 (0.69–0.94) | 0.005 | 0.84 (0.72–0.99) | 0.033 | 0.86 (0.73–1) | 0.055 | 0.9 (0.76–1.05) | 0.181 |
| Q4 | 3680 | 242 (6.6) | 47,752.8 | 0.66 (0.56–0.77) | <0.001 | 0.77 (0.65–0.92) | 0.003 | 0.8 (0.67–0.94) | 0.008 | 0.83 (0.69–0.98) | 0.031 |
| ln RAR (per SD) | 14717 | 1226 (8.3) | 186,678.5 | 1.31 (1.25–1.36) | <0.001 | 1.13 (1.06–1.21) | <0.001 | 1.11 (1.04–1.18) | 0.002 | 1.08 (1.01–1.16) | 0.026 |
| Q1 | 3662 | 187 (5.1) | 51,663.8 | 1.00 |  | 1.00 |  | 1.00 |  | 1.00 |  |
| Q2 | 3309 | 266 (8) | 43,191.4 | 1.68 (1.4–2.03) | <0.001 | 1.17 (0.97–1.41) | 0.105 | 1.16 (0.96–1.4) | 0.128 | 1.14 (0.94–1.38) | 0.174 |
| Q3 | 4055 | 341 (8.4) | 50,195.5 | 1.82 (1.52–2.18) | <0.001 | 1.05 (0.87–1.26) | 0.631 | 1.01 (0.84–1.22) | 0.893 | 1 (0.83–1.2) | 0.98 |
| Q4 | 3691 | 432 (11.7) | 41,627.8 | 2.65 (2.23–3.14) | <0.001 | 1.35 (1.12–1.64) | 0.002 | 1.29 (1.06–1.56) | 0.01 | 1.2 (0.98–1.46) | 0.072 |
| ln BAR (per SD) | 14717 | 1226 (8.3) | 186,678.5 | 1.78 (1.69–1.88) | <0.001 | 1.15 (1.07–1.23) | <0.001 | 1.16 (1.08–1.24) | <0.001 | 1.13 (1.05–1.22) | 0.002 |
| Q1 | 3649 | 138 (3.8) | 48,010.0 | 1.00 |  | 1.00 |  | 1.00 |  | 1.00 |  |
| Q2 | 3661 | 191 (5.2) | 48,566.5 | 1.35 (1.09–1.68) | 0.007 | 1 (0.8–1.26) | 0.979 | 1.04 (0.83–1.3) | 0.747 | 1.03 (0.83–1.3) | 0.768 |
| Q3 | 3698 | 290 (7.8) | 48,549.3 | 2.02 (1.65–2.47) | <0.001 | 1.08 (0.88–1.33) | 0.473 | 1.14 (0.93–1.41) | 0.213 | 1.13 (0.92–1.4) | 0.248 |
| Q4 | 3709 | 607 (16.4) | 41,552.7 | 4.51 (3.75–5.43) | <0.001 | 1.37 (1.11–1.68) | 0.003 | 1.43 (1.16–1.75) | 0.001 | 1.34 (1.08–1.66) | 0.009 |

Note: Counts, event percentages, and person-years are unweighted. Subdistribution hazard ratios (SHRs) and 95% confidence intervals (CIs) were estimated using Fine-Gray models, with noncardiovascular death treated as a competing event. Continuous ln CALLY, ln RAR, and ln BAR were standardized and analyzed per 1-SD increase; quartile analyses used Q1 as the reference. Models 1-4 followed the sequential adjustment strategy described in the primary analysis.

**Table S4.** Sensitivity analysis of the associations of ln CALLY, ln RAR, and ln BAR with all-cause and cardiovascular mortality after additional adjustment for ln NT-proBNP

| Characteristics | Participants, n | Events, n (%) | Person-years | Model 1 | | Model 2 | | Model 3 | | Model 4+ ln NT-proBNP | |
| --- | --- | --- | --- | --- | --- | --- | --- | --- | --- | --- | --- |
|  |  |  |  | HR (95% CI) | P value | HR (95% CI) | P value | HR (95% CI) | P value | HR (95% CI) | P value |
| All-cause mortality | | | | | | | | | | | |
| ln CALLY (per SD) | 6233 | 2149 (34.5) | 93,077.5 | 0.80 (0.77–0.84) | <0.001 | 0.83 (0.80–0.87) | <0.001 | 0.85 (0.81–0.89) | <0.001 | 0.86 (0.82–0.90) | <0.001 |
| Q1 | 1578 | 653 (41.4) | 22,376.7 | 1.00 |  | 1.00 |  | 1.00 |  | 1.00 |  |
| Q2 | 1650 | 591 (35.8) | 24,510.9 | 0.82 (0.73–0.92) | 0.001 | 0.81 (0.73–0.91) | <0.001 | 0.82 (0.73–0.92) | 0.001 | 0.85 (0.76–0.95) | 0.005 |
| Q3 | 1549 | 516 (33.3) | 23,394.5 | 0.75 (0.67–0.84) | <0.001 | 0.71 (0.63–0.80) | <0.001 | 0.73 (0.65–0.82) | <0.001 | 0.76 (0.67–0.85) | <0.001 |
| Q4 | 1456 | 389 (26.7) | 22,795.4 | 0.58 (0.51–0.65) | <0.001 | 0.64 (0.56–0.72) | <0.001 | 0.66 (0.58–0.75) | <0.001 | 0.68 (0.59–0.77) | <0.001 |
| ln RAR (per SD) | 6233 | 2149 (34.5) | 93,077.5 | 1.39 (1.34–1.44) | <0.001 | 1.31 (1.26–1.36) | <0.001 | 1.29 (1.24–1.34) | <0.001 | 1.24 (1.18–1.29) | <0.001 |
| Q1 | 1809 | 387 (21.4) | 30,018.4 | 1.00 |  | 1.00 |  | 1.00 |  | 1.00 |  |
| Q2 | 1474 | 506 (34.3) | 22,306.0 | 1.80 (1.57–2.05) | <0.001 | 1.29 (1.12–1.47) | <0.001 | 1.24 (1.09–1.42) | 0.001 | 1.26 (1.10–1.44) | 0.001 |
| Q3 | 1642 | 645 (39.3) | 23,610.4 | 2.20 (1.94–2.50) | <0.001 | 1.44 (1.26–1.64) | <0.001 | 1.36 (1.20–1.55) | <0.001 | 1.38 (1.21–1.57) | <0.001 |
| Q4 | 1308 | 611 (46.7) | 17,142.7 | 2.91 (2.56–3.31) | <0.001 | 2.01 (1.76–2.30) | <0.001 | 1.93 (1.69–2.21) | <0.001 | 1.83 (1.59–2.12) | <0.001 |
| ln BAR (per SD) | 6233 | 2149 (34.5) | 93,077.5 | 1.85 (1.77–1.93) | <0.001 | 1.16 (1.10–1.22) | <0.001 | 1.16 (1.11–1.22) | <0.001 | 1.06 (1.00–1.12) | 0.062 |
| Q1 | 1388 | 277 (20) | 22,160.3 | 1.00 |  | 1.00 |  | 1.00 |  | 1.00 |  |
| Q2 | 1518 | 351 (23.1) | 24,100.8 | 1.16 (0.99–1.36) | 0.058 | 0.89 (0.76–1.05) | 0.160 | 0.91 (0.78–1.07) | 0.252 | 0.89 (0.76–1.04) | 0.138 |
| Q3 | 1687 | 579 (34.3) | 25,839.9 | 1.81 (1.57–2.08) | <0.001 | 0.94 (0.81–1.09) | 0.408 | 0.99 (0.85–1.14) | 0.863 | 0.94 (0.81–1.09) | 0.411 |
| Q4 | 1640 | 942 (57.4) | 20,976.5 | 3.75 (3.28–4.29) | <0.001 | 1.15 (1.00–1.32) | 0.052 | 1.17 (1.02–1.35) | 0.029 | 0.98 (0.84–1.15) | 0.845 |
| Cardiovascular mortality | | | | | | | | | | | |
| ln CALLY (per SD) | 6233 | 697 (11.2) | 93,077.5 | 0.81 (0.75–0.87) | <0.001 | 0.84 (0.78–0.91) | <0.001 | 0.85 (0.79–0.93) | <0.001 | 0.89 (0.82–0.96) | 0.004 |
| Q1 | 1578 | 203 (12.9) | 22,376.7 | 1.00 |  | 1.00 |  | 1.00 |  | 1.00 |  |
| Q2 | 1650 | 203 (12.3) | 24,510.9 | 0.91 (0.75–1.10) | 0.325 | 0.88 (0.72–1.07) | 0.194 | 0.89 (0.73–1.08) | 0.240 | 0.94 (0.77–1.14) | 0.511 |
| Q3 | 1549 | 171 (11) | 23,394.5 | 0.80 (0.65–0.98) | 0.030 | 0.74 (0.60–0.91) | 0.004 | 0.76 (0.62–0.93) | 0.009 | 0.83 (0.67–1.02) | 0.076 |
| Q4 | 1456 | 120 (8.2) | 22,795.4 | 0.57 (0.46–0.72) | <0.001 | 0.63 (0.50–0.79) | <0.001 | 0.66 (0.52–0.83) | <0.001 | 0.70 (0.56–0.90) | 0.004 |
| ln RAR (per SD) | 6233 | 697 (11.2) | 93,077.5 | 1.41 (1.33–1.50) | <0.001 | 1.32 (1.23–1.42) | <0.001 | 1.30 (1.21–1.40) | <0.001 | 1.24 (1.14–1.34) | <0.001 |
| Q1 | 1809 | 122 (6.7) | 30,018.4 | 1.00 |  | 1.00 |  | 1.00 |  | 1.00 |  |
| Q2 | 1474 | 177 (12) | 22,306.0 | 2.00 (1.59–2.52) | <0.001 | 1.36 (1.07–1.71) | 0.011 | 1.31 (1.03–1.65) | 0.025 | 1.28 (1.01–1.63) | 0.038 |
| Q3 | 1642 | 187 (11.4) | 23,610.4 | 2.04 (1.62–2.56) | <0.001 | 1.27 (1.01–1.61) | 0.044 | 1.20 (0.95–1.52) | 0.124 | 1.17 (0.92–1.49) | 0.190 |
| Q4 | 1308 | 211 (16.1) | 17,142.7 | 3.21 (2.57–4.02) | <0.001 | 2.09 (1.65–2.64) | <0.001 | 2.00 (1.58–2.53) | <0.001 | 1.80 (1.40–2.31) | <0.001 |
| ln BAR (per SD) | 6233 | 697 (11.2) | 93,077.5 | 2.06 (1.92–2.22) | <0.001 | 1.26 (1.16–1.37) | <0.001 | 1.26 (1.16–1.37) | <0.001 | 1.16 (1.05–1.29) | 0.004 |
| Q1 | 1388 | 71 (5.1) | 22,160.3 | 1.00 |  | 1.00 |  | 1.00 |  | 1.00 |  |
| Q2 | 1518 | 105 (6.9) | 24,100.8 | 1.36 (1.00–1.83) | 0.047 | 1.04 (0.77–1.41) | 0.785 | 1.06 (0.78–1.44) | 0.700 | 1.06 (0.78–1.44) | 0.703 |
| Q3 | 1687 | 182 (10.8) | 25,839.9 | 2.21 (1.68–2.91) | <0.001 | 1.11 (0.84–1.47) | 0.459 | 1.16 (0.88–1.54) | 0.293 | 1.13 (0.85–1.50) | 0.413 |
| Q4 | 1640 | 339 (20.7) | 20,976.5 | 5.28 (4.09–6.82) | <0.001 | 1.47 (1.13–1.92) | 0.005 | 1.49 (1.14–1.95) | 0.003 | 1.30 (0.97–1.73) | 0.079 |

Note: This analysis was restricted to participants from NHANES 1999-2004 with available NT-proBNP measurements. HRs (95% CIs) were estimated using Cox proportional hazards models, with Model 4 additionally adjusted for ln NT-proBNP. Continuous ln CALLY, ln RAR, and ln BAR were standardized and analyzed per 1-SD increase; quartile analyses used Q1 as the reference.

**Table S5** Sensitivity analysis of the associations of ln CALLY, ln RAR, and ln BAR with all-cause and cardiovascular mortality after excluding deaths within the first 2 years of follow-up

| Characteristics | Participants, n | Events, n (%) | Person-years | Model 1 | | Model 2 | | Model 3 | | Model 4 | |
| --- | --- | --- | --- | --- | --- | --- | --- | --- | --- | --- | --- |
|  |  |  |  | HR (95% CI) | P value | HR (95% CI) | P value | HR (95% CI) | P value | HR (95% CI) | P value |
| All-cause mortality | | | | | | | | | | | |
| ln CALLY (per SD) | 14394 | 3478 (24.2) | 186,294.8 | 0.84 (0.81–0.87) | <0.001 | 0.85 (0.82–0.88) | <0.001 | 0.86 (0.83–0.89) | <0.001 | 0.87 (0.84–0.91) | <0.001 |
| Q1 | 3534 | 1015 (28.7) | 44,677.6 | 1.00 |  | 1.00 |  | 1.00 |  | 1.00 |  |
| Q2 | 3615 | 931 (25.8) | 46,907.6 | 0.86 (0.79–0.95) | 0.001 | 0.82 (0.75–0.89) | <0.001 | 0.82 (0.75–0.90) | <0.001 | 0.85 (0.78–0.93) | 0.001 |
| Q3 | 3607 | 810 (22.5) | 47,011.3 | 0.75 (0.69–0.83) | <0.001 | 0.69 (0.63–0.75) | <0.001 | 0.70 (0.64–0.77) | <0.001 | 0.73 (0.66–0.80) | <0.001 |
| Q4 | 3638 | 722 (19.8) | 47,698.3 | 0.66 (0.60–0.73) | <0.001 | 0.69 (0.62–0.76) | <0.001 | 0.71 (0.65–0.79) | <0.001 | 0.71 (0.65–0.79) | <0.001 |
| P for trend | 14394 | 3478 (24.2) | 186,294.8 | 0.87 (0.84–0.90) | <0.001 | 0.87 (0.85–0.90) | <0.001 | 0.88 (0.86–0.91) | <0.001 | 0.89 (0.86–0.92) | <0.001 |
| ln RAR (per SD) | 14394 | 3478 (24.2) | 186,294.8 | 1.37 (1.33–1.41) | <0.001 | 1.31 (1.27–1.36) | <0.001 | 1.30 (1.26–1.34) | <0.001 | 1.26 (1.21–1.30) | <0.001 |
| Q1 | 3637 | 569 (15.6) | 51,631.9 | 1.00 |  | 1.00 |  | 1.00 |  | 1.00 |  |
| Q2 | 3254 | 717 (22) | 43,127.6 | 1.57 (1.40–1.75) | <0.001 | 1.15 (1.03–1.29) | 0.011 | 1.13 (1.01–1.26) | 0.029 | 1.14 (1.02–1.27) | 0.022 |
| Q3 | 3978 | 1039 (26.1) | 50,098.6 | 2.01 (1.81–2.22) | <0.001 | 1.33 (1.19–1.47) | <0.001 | 1.27 (1.15–1.41) | <0.001 | 1.27 (1.14–1.41) | <0.001 |
| Q4 | 3525 | 1153 (32.7) | 41,436.7 | 2.77 (2.51–3.07) | <0.001 | 1.87 (1.68–2.08) | <0.001 | 1.79 (1.61–1.99) | <0.001 | 1.69 (1.51–1.90) | <0.001 |
| P for trend | 14394 | 3478 (24.2) | 186,294.8 | 1.39 (1.34–1.43) | <0.001 | 1.23 (1.19–1.28) | <0.001 | 1.22 (1.18–1.26) | <0.001 | 1.19 (1.15–1.23) | <0.001 |
| ln BAR (per SD) | 14394 | 3478 (24.2) | 186,294.8 | 1.84 (1.78–1.91) | <0.001 | 1.14 (1.10–1.19) | <0.001 | 1.16 (1.12–1.21) | <0.001 | 1.09 (1.04–1.14) | <0.001 |
| Q1 | 3590 | 474 (13.2) | 47,943.3 | 1.00 |  | 1.00 |  | 1.00 |  | 1.00 |  |
| Q2 | 3610 | 604 (16.7) | 48,500.8 | 1.25 (1.11–1.41) | <0.001 | 0.90 (0.80–1.02) | 0.087 | 0.94 (0.83–1.06) | 0.327 | 0.94 (0.83–1.06) | 0.303 |
| Q3 | 3646 | 846 (23.2) | 48,489.8 | 1.76 (1.57–1.97) | <0.001 | 0.87 (0.77–0.97) | 0.016 | 0.93 (0.83–1.04) | 0.193 | 0.90 (0.80–1.02) | 0.096 |
| Q4 | 3548 | 1554 (43.8) | 41,360.8 | 3.99 (3.60–4.42) | <0.001 | 1.15 (1.03–1.28) | 0.014 | 1.21 (1.09–1.35) | 0.001 | 1.07 (0.95–1.21) | 0.261 |
| P for trend | 14394 | 3478 (24.2) | 186,294.8 | 1.65 (1.60–1.70) | <0.001 | 1.07 (1.03–1.11) | <0.001 | 1.09 (1.05–1.12) | <0.001 | 1.03 (0.99–1.07) | 0.097 |
| Cardiovascular mortality | | | | | | | | | | | |
| ln CALLY (per SD) | 14394 | 1126 (7.8) | 186,294.8 | 0.83 (0.78–0.88) | <0.001 | 0.84 (0.79–0.89) | <0.001 | 0.85 (0.80–0.90) | <0.001 | 0.87 (0.82–0.93) | <0.001 |
| Q1 | 3534 | 330 (9.3) | 44,677.6 | 1.00 |  | 1.00 |  | 1.00 |  | 1.00 |  |
| Q2 | 3615 | 300 (8.3) | 46,907.6 | 0.86 (0.73–1.00) | 0.053 | 0.80 (0.68–0.94) | 0.006 | 0.81 (0.69–0.94) | 0.007 | 0.84 (0.72–0.99) | 0.036 |
| Q3 | 3607 | 268 (7.4) | 47,011.3 | 0.77 (0.65–0.90) | 0.001 | 0.69 (0.59–0.81) | <0.001 | 0.70 (0.60–0.83) | <0.001 | 0.76 (0.64–0.89) | 0.001 |
| Q4 | 3638 | 228 (6.3) | 47,698.3 | 0.64 (0.54–0.76) | <0.001 | 0.67 (0.56–0.79) | <0.001 | 0.69 (0.58–0.82) | <0.001 | 0.72 (0.60–0.86) | <0.001 |
| P for trend | 14394 | 1126 (7.8) | 186,294.8 | 0.87 (0.82–0.91) | <0.001 | 0.87 (0.82–0.92) | <0.001 | 0.88 (0.83–0.93) | <0.001 | 0.89 (0.84–0.94) | <0.001 |
| ln RAR (per SD) | 14394 | 1126 (7.8) | 186,294.8 | 1.39 (1.32–1.46) | <0.001 | 1.32 (1.25–1.40) | <0.001 | 1.31 (1.24–1.39) | <0.001 | 1.25 (1.18–1.34) | <0.001 |
| Q1 | 3637 | 176 (4.8) | 51,631.9 | 1.00 |  | 1.00 |  | 1.00 |  | 1.00 |  |
| Q2 | 3254 | 246 (7.6) | 43,127.6 | 1.75 (1.44–2.12) | <0.001 | 1.23 (1.01–1.50) | 0.036 | 1.22 (1.00–1.48) | 0.049 | 1.20 (0.99–1.46) | 0.065 |
| Q3 | 3978 | 315 (7.9) | 50,098.6 | 1.98 (1.65–2.38) | <0.001 | 1.24 (1.03–1.49) | 0.027 | 1.20 (0.99–1.45) | 0.062 | 1.17 (0.97–1.42) | 0.101 |
| Q4 | 3525 | 389 (11) | 41,436.7 | 3.05 (2.55–3.65) | <0.001 | 1.92 (1.59–2.32) | <0.001 | 1.86 (1.54–2.25) | <0.001 | 1.67 (1.37–2.04) | <0.001 |
| P for trend | 14394 | 1126 (7.8) | 186,294.8 | 1.41 (1.33–1.49) | <0.001 | 1.23 (1.16–1.31) | <0.001 | 1.22 (1.15–1.29) | <0.001 | 1.17 (1.10–1.25) | <0.001 |
| ln BAR (per SD) | 14394 | 1126 (7.8) | 186,294.8 | 2.07 (1.95–2.19) | <0.001 | 1.25 (1.17–1.34) | <0.001 | 1.27 (1.19–1.36) | <0.001 | 1.20 (1.11–1.30) | <0.001 |
| Q1 | 3590 | 119 (3.3) | 47,943.3 | 1.00 |  | 1.00 |  | 1.00 |  | 1.00 |  |
| Q2 | 3610 | 177 (4.9) | 48,500.8 | 1.46 (1.16–1.84) | 0.001 | 1.02 (0.81–1.29) | 0.871 | 1.06 (0.84–1.34) | 0.626 | 1.07 (0.85–1.36) | 0.569 |
| Q3 | 3646 | 273 (7.5) | 48,489.8 | 2.25 (1.82–2.79) | <0.001 | 1.04 (0.84–1.30) | 0.697 | 1.11 (0.89–1.38) | 0.370 | 1.09 (0.87–1.37) | 0.436 |
| Q4 | 3548 | 557 (15.7) | 41,360.8 | 5.70 (4.68–6.95) | <0.001 | 1.46 (1.19–1.80) | <0.001 | 1.54 (1.25–1.89) | <0.001 | 1.37 (1.10–1.72) | 0.006 |
| P for trend | 14394 | 1126 (7.8) | 186,294.8 | 1.87 (1.76–1.99) | <0.001 | 1.16 (1.09–1.24) | <0.001 | 1.18 (1.11–1.25) | <0.001 | 1.12 (1.04–1.20) | 0.001 |

Note: Counts, event percentages, and person-years are unweighted. HRs (95% CIs) were estimated using Cox proportional hazards models. Continuous ln CALLY, ln RAR, and ln BAR were standardized and analyzed per 1-SD increase; quartile analyses used Q1 as the reference. Models 1-4 followed the sequential adjustment strategy described in the primary analysis.

**Table S6** Sensitivity analysis of the associations of ln CALLY, ln RAR, and ln BAR with all-cause and cardiovascular mortality after multiple imputation for missing covariate data

| Characteristics | Participants, n | Events, n (%) | Person-years | Model 1 | P value | Model 2 | P value | Model 3 | P value | Model 4 | P value |
| --- | --- | --- | --- | --- | --- | --- | --- | --- | --- | --- | --- |
|  |  |  |  | HR (95% CI) |  | HR(95% CI) |  | HR(95% CI) |  | HR (95% CI) |  |
| All-cause mortality |  |  |  |  |  |  |  |  |  |  |  |
| ln CALLY (per SD) | 17321 | 4538 (26.2) | 219640.8 | 0.82 (0.8–0.85) | <0.001 | 0.84 (0.81–0.86) | <0.001 | 0.85 (0.83–0.88) | <0.001 | 0.86 (0.83–0.89) | <0.001 |
| Q1 | 4330 | 1359 (31.4) | 52980.0 | 1.00 |  | 1.00 |  | 1.00 |  | 1.00 |  |
| Q2 | 4330 | 1200 (27.7) | 55309.6 | 0.84 (0.78–0.91) | <0.001 | 0.8 (0.74–0.86) | <0.001 | 0.81 (0.75–0.87) | <0.001 | 0.83 (0.77–0.9) | <0.001 |
| Q3 | 4329 | 1053 (24.3) | 55285.8 | 0.74 (0.68–0.8) | <0.001 | 0.68 (0.63–0.74) | <0.001 | 0.7 (0.65–0.76) | <0.001 | 0.73 (0.67–0.79) | <0.001 |
| Q4 | 4332 | 926 (21.4) | 56065.3 | 0.64 (0.59–0.7) | <0.001 | 0.66 (0.61–0.72) | <0.001 | 0.69 (0.64–0.76) | <0.001 | 0.69 (0.63–0.75) | <0.001 |
|  |  |  |  |  |  |  |  |  |  |  |  |
| ln RAR (per SD) | 17321 | 4538 (26.2) | 219640.8 | 1.4 (1.37–1.44) | <0.001 | 1.34 (1.31–1.38) | <0.001 | 1.33 (1.29–1.37) | <0.001 | 1.28 (1.25–1.32) | <0.001 |
| Q1 | 4246 | 703 (16.6) | 60160.8 | 1.00 |  | 1.00 |  | 1.00 |  | 1.00 |  |
| Q2 | 3981 | 943 (23.7) | 52187.9 | 1.6 (1.45–1.76) | <0.001 | 1.16 (1.05–1.28) | 0.004 | 1.14 (1.03–1.26) | 0.009 | 1.15 (1.04–1.27) | 0.005 |
| Q3 | 4739 | 1332 (28.1) | 58356.1 | 2.07 (1.88–2.26) | <0.001 | 1.33 (1.21–1.46) | <0.001 | 1.29 (1.17–1.41) | <0.001 | 1.29 (1.17–1.41) | <0.001 |
| Q4 | 4355 | 1560 (35.8) | 48935.9 | 2.95 (2.69–3.22) | <0.001 | 1.94 (1.76–2.13) | <0.001 | 1.85 (1.68–2.03) | <0.001 | 1.75 (1.58–1.93) | <0.001 |
|  |  |  |  |  |  |  |  |  |  |  |  |
| ln BAR (per SD) | 17321 | 4538 (26.2) | 219640.8 | 1.84 (1.79–1.9) | <0.001 | 1.15 (1.12–1.19) | <0.001 | 1.17 (1.14–1.21) | <0.001 | 1.09 (1.05–1.14) | <0.001 |
| Q1 | 4314 | 637 (14.8) | 56803.3 | 1.00 |  | 1.00 |  | 1.00 |  | 1.00 |  |
| Q2 | 4287 | 780 (18.2) | 56984.6 | 1.21 (1.09–1.35) | <0.001 | 0.88 (0.79–0.98) | 0.015 | 0.91 (0.82–1.01) | 0.083 | 0.91 (0.82–1.01) | 0.078 |
| Q3 | 4380 | 1080 (24.7) | 57581.9 | 1.66 (1.51–1.84) | <0.001 | 0.83 (0.75–0.92) | <0.001 | 0.89 (0.8–0.98) | 0.018 | 0.86 (0.78–0.95) | 0.004 |
| Q4 | 4340 | 2041 (47) | 48270.9 | 3.91 (3.58–4.28) | <0.001 | 1.14 (1.04–1.26) | 0.006 | 1.21 (1.1–1.33) | <0.001 | 1.05 (0.95–1.16) | 0.359 |
|  |  |  |  |  |  |  |  |  |  |  |  |
| Cardiovascular mortality | | | | | | | | | | | |
| ln CALLY (per SD) | 17321 | 1444 (8.3) | 219640.8 | 0.84 (0.8–0.89) | <0.001 | 0.86 (0.81–0.91) | <0.001 | 0.87 (0.83–0.92) | <0.001 | 0.89 (0.84–0.94) | <0.001 |
| Q1 | 4330 | 416 (9.6) | 52980.0 | 1.00 |  | 1.00 |  | 1.00 |  | 1.00 |  |
| Q2 | 4330 | 383 (8.8) | 55309.6 | 0.87 (0.76–1.01) | 0.059 | 0.82 (0.72–0.95) | 0.007 | 0.83 (0.73–0.96) | 0.011 | 0.87 (0.76–1) | 0.052 |
| Q3 | 4329 | 354 (8.2) | 55285.8 | 0.81 (0.7–0.94) | 0.004 | 0.74 (0.65–0.86) | <0.001 | 0.77 (0.66–0.88) | <0.001 | 0.82 (0.71–0.95) | 0.008 |
| Q4 | 4332 | 291 (6.7) | 56065.3 | 0.66 (0.57–0.76) | <0.001 | 0.68 (0.59–0.79) | <0.001 | 0.71 (0.61–0.83) | <0.001 | 0.73 (0.63–0.86) | <0.001 |
|  |  |  |  |  |  |  |  |  |  |  |  |
| ln RAR (per SD) | 17321 | 1444 (8.3) | 219640.8 | 1.41 (1.35–1.47) | <0.001 | 1.32 (1.26–1.39) | <0.001 | 1.31 (1.25–1.38) | <0.001 | 1.25 (1.18–1.32) | <0.001 |
| Q1 | 4246 | 215 (5.1) | 60160.8 | 1.00 |  | 1.00 |  | 1.00 |  | 1.00 |  |
| Q2 | 3981 | 315 (7.9) | 52187.9 | 1.75 (1.47–2.08) | <0.001 | 1.21 (1.02–1.44) | 0.032 | 1.2 (1.01–1.43) | 0.039 | 1.19 (1–1.42) | 0.051 |
| Q3 | 4739 | 405 (8.5) | 58356.1 | 2.07 (1.75–2.44) | <0.001 | 1.25 (1.06–1.49) | 0.009 | 1.22 (1.03–1.45) | 0.021 | 1.19 (1–1.41) | 0.053 |
| Q4 | 4355 | 509 (11.7) | 48935.9 | 3.17 (2.7–3.72) | <0.001 | 1.92 (1.62–2.27) | <0.001 | 1.85 (1.56–2.19) | <0.001 | 1.65 (1.38–1.98) | <0.001 |
|  |  |  |  |  |  |  |  |  |  |  |  |
| ln BAR (per SD) | 17321 | 1444 (8.3) | 219640.8 | 2.04 (1.94–2.14) | <0.001 | 1.25 (1.18–1.33) | <0.001 | 1.27 (1.2–1.35) | <0.001 | 1.18 (1.1–1.27) | <0.001 |
| Q1 | 4314 | 165 (3.8) | 56803.3 | 1.00 |  | 1.00 |  | 1.00 |  | 1.00 |  |
| Q2 | 4287 | 227 (5.3) | 56984.6 | 1.36 (1.11–1.66) | 0.003 | 0.97 (0.79–1.18) | 0.73 | 1 (0.81–1.22) | 0.978 | 1 (0.82–1.23) | 0.977 |
| Q3 | 4380 | 339 (7.7) | 57581.9 | 2.01 (1.67–2.42) | <0.001 | 0.95 (0.79–1.15) | 0.608 | 1.01 (0.83–1.22) | 0.936 | 0.98 (0.8–1.19) | 0.811 |
| Q4 | 4340 | 713 (16.4) | 48270.9 | 5.28 (4.46–6.25) | <0.001 | 1.41 (1.18–1.68) | <0.001 | 1.48 (1.23–1.77) | <0.001 | 1.26 (1.04–1.54) | 0.018 |

Note: This sensitivity analysis was performed after multiple imputation by chained equations for missing covariate data; five imputed datasets were generated, and estimates were pooled using Rubin's rules. HRs (95% CIs) were estimated using Cox proportional hazards models. Continuous ln CALLY, ln RAR, and ln BAR were standardized and analyzed per 1-SD increase; quartile analyses used Q1 as the reference. Models 1-4 followed the sequential adjustment strategy described in the primary analysis.

**Supplementary Table S7. Sensitivity analysis of the associations of ln CALLY, ln RAR, and ln BAR with all-cause and cardiovascular mortality after additional adjustment for baseline medication use among adults with preclinical heart failure**

| Characteristics | Participants, n | Events, n (%) | Person-years | Model 1 | | Model 2 | | Model 3 | | Model 4 | |
| --- | --- | --- | --- | --- | --- | --- | --- | --- | --- | --- | --- |
|  |  |  |  | HR (95% CI) | P value | HR (95% CI) | P value | HR (95% CI) | P value | HR (95% CI) | P value |
| All-cause mortality |  |  |  |  |  |  |  |  |  |  |  |
| ln CALLY ( per-SD) | 14,713 | 3,800 (25.8) | 186,629.1 | 0.82 (0.79–0.84) | <0.001 | 0.83 (0.81–0.86) | <0.001 | 0.84 (0.82–0.87) | <0.001 | 0.85 (0.82–0.88) | <0.001 |
| Q1 (reference) | 3,672 | 1,154 (31.4) | 44,819.8 | 1.00 (Ref) |  | 1.00 (Ref) |  | 1.00 (Ref) |  | 1.00 (Ref) |  |
| Q2 | 3,682 | 999 (27.1) | 46,973.3 | 0.82 (0.75–0.89) | <0.001 | 0.79 (0.73–0.86) | <0.001 | 0.79 (0.73–0.86) | <0.001 | 0.81 (0.75–0.89) | <0.001 |
| Q3 | 3,679 | 883 (24.0) | 47,083.3 | 0.72 (0.66–0.79) | <0.001 | 0.68 (0.62–0.74) | <0.001 | 0.69 (0.63–0.76) | <0.001 | 0.71 (0.65–0.78) | <0.001 |
| Q4 | 3,680 | 764 (20.8) | 47,752.8 | 0.62 (0.56–0.68) | <0.001 | 0.65 (0.59–0.71) | <0.001 | 0.68 (0.62–0.74) | <0.001 | 0.67 (0.61–0.74) | <0.001 |
| ln RAR ( per-SD) | 14,713 | 3,800 (25.8) | 186,629.1 | 1.40 (1.36–1.44) | <0.001 | 1.33 (1.29–1.37) | <0.001 | 1.31 (1.27–1.35) | <0.001 | 1.28 (1.24–1.32) | <0.001 |
| Q1 (reference) | 3,660 | 593 (16.2) | 51,643.5 | 1.00 (Ref) |  | 1.00 (Ref) |  | 1.00 (Ref) |  | 1.00 (Ref) |  |
| Q2 | 3,309 | 772 (23.3) | 43,191.4 | 1.60 (1.44–1.79) | <0.001 | 1.18 (1.06–1.31) | 0.003 | 1.16 (1.04–1.29) | 0.008 | 1.17 (1.05–1.30) | 0.006 |
| Q3 | 4,054 | 1,116 (27.5) | 50,180.3 | 2.04 (1.84–2.25) | <0.001 | 1.34 (1.21–1.48) | <0.001 | 1.28 (1.16–1.42) | <0.001 | 1.28 (1.15–1.42) | <0.001 |
| Q4 | 3,690 | 1,319 (35.7) | 41,613.8 | 2.97 (2.69–3.27) | <0.001 | 1.90 (1.72–2.11) | <0.001 | 1.83 (1.65–2.03) | <0.001 | 1.76 (1.58–1.96) | <0.001 |
| ln BAR ( per-SD) | 14,713 | 3,800 (25.8) | 186,629.1 | 1.83 (1.78–1.89) | <0.001 | 1.11 (1.07–1.16) | <0.001 | 1.14 (1.10–1.18) | <0.001 | 1.08 (1.04–1.13) | <0.001 |
| Q1 (reference) | 3,648 | 533 (14.6) | 47,996.1 | 1.00 (Ref) |  | 1.00 (Ref) |  | 1.00 (Ref) |  | 1.00 (Ref) |  |
| Q2 | 3,661 | 655 (17.9) | 48,566.5 | 1.21 (1.08–1.35) | 0.001 | 0.87 (0.78–0.98) | 0.018 | 0.91 (0.81–1.02) | 0.104 | 0.91 (0.81–1.02) | 0.107 |
| Q3 | 3,695 | 897 (24.3) | 48,513.8 | 1.66 (1.49–1.84) | <0.001 | 0.82 (0.73–0.91) | <0.001 | 0.87 (0.78–0.97) | 0.014 | 0.86 (0.77–0.96) | 0.008 |
| Q4 | 3,709 | 1,715 (46.2) | 41,552.7 | 3.85 (3.49–4.24) | <0.001 | 1.06 (0.95–1.17) | 0.29 | 1.12 (1.01–1.25) | 0.028 | 1.02 (0.91–1.15) | 0.686 |
| Cardiovascular mortality |  |  |  |  |  |  |  |  |  |  |  |
| ln CALLY ( per-SD) | 14,713 | 1,225 (8.3) | 186,629.1 | 0.82 (0.77–0.87) | <0.001 | 0.84 (0.79–0.89) | <0.001 | 0.85 (0.80–0.90) | <0.001 | 0.87 (0.82–0.93) | <0.001 |
| Q1 (reference) | 3,672 | 366 (10.0) | 44,819.8 | 1.00 (Ref) |  | 1.00 (Ref) |  | 1.00 (Ref) |  | 1.00 (Ref) |  |
| Q2 | 3,682 | 322 (8.7) | 46,973.3 | 0.83 (0.72–0.97) | 0.016 | 0.80 (0.69–0.93) | 0.004 | 0.81 (0.69–0.94) | 0.005 | 0.83 (0.71–0.96) | 0.015 |
| Q3 | 3,679 | 295 (8.0) | 47,083.3 | 0.76 (0.65–0.89) | 0.001 | 0.72 (0.61–0.84) | <0.001 | 0.73 (0.62–0.85) | <0.001 | 0.77 (0.66–0.90) | 0.001 |
| Q4 | 3,680 | 242 (6.6) | 47,752.8 | 0.62 (0.52–0.72) | <0.001 | 0.65 (0.56–0.77) | <0.001 | 0.68 (0.58–0.80) | <0.001 | 0.70 (0.59–0.83) | <0.001 |
| ln RAR ( per-SD) | 14,713 | 1,225 (8.3) | 186,629.1 | 1.39 (1.33–1.46) | <0.001 | 1.29 (1.22–1.36) | <0.001 | 1.28 (1.21–1.35) | <0.001 | 1.24 (1.17–1.32) | <0.001 |
| Q1 (reference) | 3,660 | 186 (5.1) | 51,643.5 | 1.00 (Ref) |  | 1.00 (Ref) |  | 1.00 (Ref) |  | 1.00 (Ref) |  |
| Q2 | 3,309 | 266 (8.0) | 43,191.4 | 1.77 (1.47–2.14) | <0.001 | 1.25 (1.04–1.51) | 0.02 | 1.24 (1.03–1.50) | 0.025 | 1.22 (1.01–1.48) | 0.04 |
| Q3 | 4,054 | 341 (8.4) | 50,180.3 | 2.00 (1.67–2.39) | <0.001 | 1.24 (1.04–1.49) | 0.019 | 1.20 (1.00–1.44) | 0.048 | 1.17 (0.97–1.40) | 0.104 |
| Q4 | 3,690 | 432 (11.7) | 41,613.8 | 3.13 (2.63–3.72) | <0.001 | 1.85 (1.54–2.22) | <0.001 | 1.79 (1.49–2.15) | <0.001 | 1.65 (1.36–2.00) | <0.001 |
| ln BAR ( per-SD) | 14,713 | 1,225 (8.3) | 186,629.1 | 2.03 (1.93–2.14) | <0.001 | 1.18 (1.11–1.26) | <0.001 | 1.20 (1.13–1.28) | <0.001 | 1.17 (1.08–1.26) | <0.001 |
| Q1 (reference) | 3,648 | 138 (3.8) | 47,996.1 | 1.00 (Ref) |  | 1.00 (Ref) |  | 1.00 (Ref) |  | 1.00 (Ref) |  |
| Q2 | 3,661 | 191 (5.2) | 48,566.5 | 1.36 (1.09–1.69) | 0.006 | 0.95 (0.76–1.18) | 0.637 | 0.99 (0.79–1.23) | 0.913 | 1.00 (0.80–1.25) | 0.998 |
| Q3 | 3,695 | 289 (7.8) | 48,513.8 | 2.06 (1.68–2.52) | <0.001 | 0.95 (0.77–1.17) | 0.612 | 1.01 (0.82–1.24) | 0.937 | 1.00 (0.81–1.24) | 0.972 |
| Q4 | 3,709 | 607 (16.4) | 41,552.7 | 5.27 (4.38–6.34) | <0.001 | 1.25 (1.03–1.52) | 0.024 | 1.33 (1.09–1.62) | 0.005 | 1.24 (1.00–1.54) | 0.045 |

**Notes:** Hazard ratios (HRs) and 95% confidence intervals (CIs) were estimated using Cox proportional hazards regression models. CALLY, RAR, and BAR were natural log-transformed before analysis. HRs for continuous variables are presented per 1-standard deviation (SD) increase in each ln-transformed index. Model 1 was unadjusted. Model 2 was adjusted for age, sex, race/ethnicity, marital status, poverty-to-income ratio, and educational level. Model 3 was additionally adjusted for smoking status, alcohol consumption, and physical activity. Model 4 was further adjusted for body mass index, stroke, angina, hypertension, hyperlipidemia, diabetes mellitus, alanine aminotransferase, aspartate aminotransferase, total bilirubin, estimated glomerular filtration rate, hemoglobin, cancer history, antihypertensive medication use, glucose-lowering medication use, and lipid-lowering medication use.

**Abbreviations:** BAR, blood urea nitrogen–albumin ratio; CALLY, C-reactive protein–albumin–lymphocyte index; CI, confidence interval; HR, hazard ratio; RAR, red cell distribution width–albumin ratio; SD, standard deviation.

**Supplementary Table S8. Associations of ln CALLY, ln RAR, and ln BAR with all-cause and cardiovascular mortality among adults with obesity as the only PHF component**

| Characteristics | Participants, n | Events, n (%) | Person-years | Model 1 | | Model 2 | | Model 3 | | Model 4 | |
| --- | --- | --- | --- | --- | --- | --- | --- | --- | --- | --- | --- |
|  |  |  |  | HR (95% CI) | P value | HR (95% CI) | P value | HR (95% CI) | P value | HR (95% CI) | P value |
| All-cause mortality | | | | | | | | | | | |
| ln CALLY (per-SD) | 2,259 | 133 (5.9) | 31,204.2 | 0.82 (0.68–1.00) | 0.051 | 0.75 (0.61–0.93) | 0.007 | 0.78 (0.63–0.96) | 0.017 | 0.77 (0.62–0.96) | 0.018 |
| Q1 (reference) | 658 | 48 (7.3) | 9,007.3 | 1.00 (Ref) |  | 1.00 (Ref) |  | 1.00 (Ref) |  | 1.00 (Ref) |  |
| Q2 | 671 | 38 (5.7) | 9,494.8 | 0.73 (0.48–1.12) | 0.153 | 0.91 (0.59–1.42) | 0.692 | 0.98 (0.62–1.53) | 0.921 | 0.93 (0.59–1.46) | 0.744 |
| Q3 | 555 | 33 (5.9) | 7,572.3 | 0.82 (0.53–1.28) | 0.387 | 0.68 (0.42–1.10) | 0.117 | 0.77 (0.47–1.25) | 0.291 | 0.74 (0.45–1.22) | 0.243 |
| Q4 | 375 | 14 (3.7) | 5,129.8 | 0.51 (0.28–0.93) | 0.029 | 0.46 (0.25–0.84) | 0.012 | 0.47 (0.26–0.87) | 0.017 | 0.46 (0.24–0.88) | 0.018 |
| ln RAR (per-SD) | 2,259 | 133 (5.9) | 31,204.2 | 1.32 (1.16–1.50) | <0.001 | 1.45 (1.23–1.71) | <0.001 | 1.43 (1.20–1.70) | <0.001 | 1.40 (1.14–1.70) | 0.001 |
| Q1 (reference) | 492 | 16 (3.3) | 7,087.4 | 1.00 (Ref) |  | 1.00 (Ref) |  | 1.00 (Ref) |  | 1.00 (Ref) |  |
| Q2 | 504 | 29 (5.8) | 7,102.3 | 1.87 (1.01–3.44) | 0.045 | 1.44 (0.77–2.71) | 0.253 | 1.35 (0.71–2.55) | 0.357 | 1.27 (0.66–2.43) | 0.47 |
| Q3 | 684 | 39 (5.7) | 9,355.8 | 1.96 (1.09–3.51) | 0.024 | 1.52 (0.83–2.81) | 0.178 | 1.50 (0.81–2.76) | 0.196 | 1.36 (0.73–2.54) | 0.34 |
| Q4 | 579 | 49 (8.5) | 7,658.6 | 3.07 (1.74–5.41) | <0.001 | 2.49 (1.34–4.65) | 0.004 | 2.18 (1.17–4.06) | 0.015 | 1.91 (0.99–3.69) | 0.054 |
| ln BAR (per-SD) | 2,259 | 133 (5.9) | 31,204.2 | 1.76 (1.41–2.19) | <0.001 | 0.87 (0.70–1.07) | 0.181 | 0.88 (0.71–1.10) | 0.261 | 0.95 (0.75–1.19) | 0.633 |
| Q1 (reference) | 787 | 33 (4.2) | 10,777.0 | 1.00 (Ref) |  | 1.00 (Ref) |  | 1.00 (Ref) |  | 1.00 (Ref) |  |
| Q2 | 692 | 23 (3.3) | 9,662.7 | 0.77 (0.45–1.30) | 0.325 | 0.63 (0.37–1.08) | 0.093 | 0.63 (0.36–1.08) | 0.094 | 0.67 (0.39–1.17) | 0.16 |
| Q3 | 519 | 40 (7.7) | 7,186.4 | 1.78 (1.12–2.82) | 0.015 | 0.80 (0.49–1.30) | 0.373 | 0.81 (0.49–1.32) | 0.391 | 0.93 (0.56–1.54) | 0.783 |
| Q4 | 261 | 37 (14.2) | 3,578.1 | 3.33 (2.08–5.32) | <0.001 | 0.77 (0.45–1.32) | 0.339 | 0.82 (0.48–1.41) | 0.471 | 0.94 (0.53–1.66) | 0.837 |
| Cardiovascular mortality | | | | | | | | | | | |
| ln CALLY (per-SD) | 2,259 | 30 (1.3) | 31,204.2 | 0.59 (0.39–0.89) | 0.013 | 0.42 (0.26–0.66) | <0.001 | 0.41 (0.25–0.66) | <0.001 | 0.44 (0.26–0.74) | 0.002 |
| Q1 (reference) | 658 | 14 (2.1) | 9,007.3 | 1.00 (Ref) |  | 1.00 (Ref) |  | 1.00 (Ref) |  | 1.00 (Ref) |  |
| Q2 | 671 | 8 (1.2) | 9,494.8 | 0.53 (0.22–1.26) | 0.151 | 0.62 (0.25–1.54) | 0.301 | 0.63 (0.24–1.61) | 0.33 | 0.59 (0.22–1.57) | 0.29 |
| Q3 | 555 | 5 (0.9) | 7,572.3 | 0.43 (0.15–1.18) | 0.102 | 0.21 (0.07–0.66) | 0.007 | 0.22 (0.07–0.71) | 0.011 | 0.22 (0.07–0.75) | 0.015 |
| Q4 | 375 | 3 (0.8) | 5,129.8 | 0.38 (0.11–1.31) | 0.125 | 0.21 (0.06–0.77) | 0.018 | 0.20 (0.05–0.74) | 0.016 | 0.28 (0.07–1.10) | 0.069 |
| ln RAR (per-SD) | 2,259 | 30 (1.3) | 31,204.2 | 1.34 (1.03–1.76) | 0.031 | 1.57 (1.10–2.24) | 0.014 | 1.56 (1.07–2.29) | 0.021 | 1.42 (0.92–2.20) | 0.116 |
| Q1 (reference) | 492 | 3 (0.6) | 7,087.4 | 1.00 (Ref) |  | 1.00 (Ref) |  | 1.00 (Ref) |  | 1.00 (Ref) |  |
| Q2 | 504 | 7 (1.4) | 7,102.3 | 2.42 (0.62–9.36) | 0.201 | 1.32 (0.31–5.63) | 0.706 | 1.22 (0.28–5.32) | 0.789 | 1.34 (0.29–6.22) | 0.71 |
| Q3 | 684 | 8 (1.2) | 9,355.8 | 2.14 (0.57–8.10) | 0.26 | 1.23 (0.30–5.03) | 0.774 | 1.28 (0.31–5.23) | 0.735 | 1.16 (0.26–5.17) | 0.843 |
| Q4 | 579 | 12 (2.1) | 7,658.6 | 4.02 (1.13–14.28) | 0.032 | 2.50 (0.62–10.17) | 0.199 | 2.10 (0.51–8.66) | 0.303 | 1.76 (0.38–8.11) | 0.471 |
| ln BAR (per-SD) | 2,259 | 30 (1.3) | 31,204.2 | 3.12 (1.99–4.90) | <0.001 | 1.15 (0.74–1.79) | 0.536 | 1.21 (0.77–1.91) | 0.409 | 1.45 (0.84–2.49) | 0.185 |
| Q1 (reference) | 787 | 5 (0.6) | 10,777.0 | 1.00 (Ref) |  | 1.00 (Ref) |  | 1.00 (Ref) |  | 1.00 (Ref) |  |
| Q2 | 692 | 4 (0.6) | 9,662.7 | 0.88 (0.24–3.27) | 0.846 | 0.64 (0.17–2.42) | 0.507 | 0.67 (0.17–2.59) | 0.563 | 0.66 (0.16–2.72) | 0.564 |
| Q3 | 519 | 6 (1.2) | 7,186.4 | 1.76 (0.54–5.76) | 0.352 | 0.68 (0.20–2.34) | 0.537 | 0.64 (0.18–2.25) | 0.482 | 0.70 (0.18–2.65) | 0.595 |
| Q4 | 261 | 15 (5.7) | 3,578.1 | 8.94 (3.25–24.60) | <0.001 | 1.73 (0.54–5.53) | 0.354 | 1.93 (0.60–6.20) | 0.269 | 2.89 (0.82–10.13) | 0.097 |

**Notes:** Analyses were restricted to participants with obesity as the only PHF component. Participants with hypertension, diabetes mellitus, and ASCVD were excluded from this isolated phenotype-specific sensitivity analysis. Hazard ratios (HRs) and 95% confidence intervals (CIs) were estimated using Cox proportional hazards regression models. CALLY, RAR, and BAR were natural log-transformed before analysis. HRs for continuous variables are presented per 1-standard deviation (SD) increase in each ln-transformed index. Model 1 was unadjusted. Model 2 was adjusted for age, sex, race/ethnicity, marital status, poverty-to-income ratio, and educational level. Model 3 was additionally adjusted for smoking status, alcohol consumption, and physical activity. Model 4 was further adjusted for the prespecified clinical and laboratory covariates that retained variability within the restricted sample. Phenotype-defining variables with no variation in the restricted sample were omitted from the model. Obesity was defined as a body mass index of ≥30 kg/m².

**Abbreviations:** BAR, blood urea nitrogen–albumin ratio; BMI, body mass index; CALLY, C-reactive protein–albumin–lymphocyte index; CI, confidence interval; HR, hazard ratio; RAR, red cell distribution width–albumin ratio; SD, standard deviation.

**Supplementary Table S9. Associations of ln CALLY, ln RAR, and ln BAR with all-cause and cardiovascular mortality among adults with hypertension as the only PHF component**

| Characteristics | Participants, n | Events, n (%) | Person-years | Model 1 | | Model 2 | | Model 3 | | Model 4 | |
| --- | --- | --- | --- | --- | --- | --- | --- | --- | --- | --- | --- |
|  |  |  |  | HR (95% CI) | P value | HR (95% CI) | P value | HR (95% CI) | P value | HR (95% CI) | P value |
| All-cause mortality | | | | | | | | | | | |
| ln CALLY (per-SD) | 5,046 | 1,348 (26.7) | 65,358.3 | 0.68 (0.65–0.71) | <0.001 | 0.82 (0.77–0.86) | <0.001 | 0.84 (0.80–0.89) | <0.001 | 0.85 (0.80–0.90) | <0.001 |
| Q1 (reference) | 778 | 324 (41.6) | 9,140.1 | 1.00 (Ref) |  | 1.00 (Ref) |  | 1.00 (Ref) |  | 1.00 (Ref) |  |
| Q2 | 1,059 | 366 (34.6) | 13,407.7 | 0.76 (0.66–0.89) | <0.001 | 0.75 (0.65–0.87) | <0.001 | 0.76 (0.65–0.89) | <0.001 | 0.81 (0.70–0.95) | 0.008 |
| Q3 | 1,341 | 342 (25.5) | 17,560.5 | 0.54 (0.47–0.63) | <0.001 | 0.61 (0.53–0.72) | <0.001 | 0.66 (0.56–0.77) | <0.001 | 0.70 (0.60–0.82) | <0.001 |
| Q4 | 1,868 | 316 (16.9) | 25,250.1 | 0.35 (0.30–0.40) | <0.001 | 0.58 (0.50–0.68) | <0.001 | 0.63 (0.54–0.74) | <0.001 | 0.63 (0.53–0.74) | <0.001 |
| ln RAR (per-SD) | 5,046 | 1,348 (26.7) | 65,358.3 | 1.63 (1.56–1.69) | <0.001 | 1.41 (1.33–1.49) | <0.001 | 1.38 (1.30–1.46) | <0.001 | 1.32 (1.24–1.40) | <0.001 |
| Q1 (reference) | 1,701 | 252 (14.8) | 24,615.2 | 1.00 (Ref) |  | 1.00 (Ref) |  | 1.00 (Ref) |  | 1.00 (Ref) |  |
| Q2 | 1,227 | 290 (23.6) | 16,098.2 | 1.83 (1.54–2.16) | <0.001 | 1.11 (0.94–1.32) | 0.222 | 1.08 (0.91–1.28) | 0.393 | 1.06 (0.89–1.26) | 0.49 |
| Q3 | 1,270 | 424 (33.4) | 15,460.3 | 2.86 (2.45–3.35) | <0.001 | 1.36 (1.16–1.60) | <0.001 | 1.27 (1.08–1.49) | 0.004 | 1.23 (1.05–1.45) | 0.013 |
| Q4 | 848 | 382 (45.0) | 9,184.8 | 4.47 (3.81–5.25) | <0.001 | 1.97 (1.67–2.34) | <0.001 | 1.83 (1.55–2.17) | <0.001 | 1.68 (1.41–2.01) | <0.001 |
| ln BAR (per-SD) | 5,046 | 1,348 (26.7) | 65,358.3 | 1.63 (1.54–1.73) | <0.001 | 1.03 (0.97–1.09) | 0.344 | 1.06 (1.00–1.12) | 0.062 | 0.99 (0.93–1.06) | 0.856 |
| Q1 (reference) | 1,320 | 241 (18.3) | 17,622.7 | 1.00 (Ref) |  | 1.00 (Ref) |  | 1.00 (Ref) |  | 1.00 (Ref) |  |
| Q2 | 1,223 | 234 (19.1) | 16,716.0 | 1.02 (0.85–1.22) | 0.855 | 0.74 (0.61–0.88) | 0.001 | 0.77 (0.64–0.93) | 0.006 | 0.77 (0.64–0.92) | 0.005 |
| Q3 | 1,285 | 324 (25.2) | 17,065.5 | 1.39 (1.18–1.64) | <0.001 | 0.73 (0.62–0.87) | <0.001 | 0.78 (0.66–0.93) | 0.005 | 0.76 (0.64–0.91) | 0.003 |
| Q4 | 1,218 | 549 (45.1) | 13,954.2 | 2.99 (2.57–3.47) | <0.001 | 0.91 (0.77–1.07) | 0.237 | 0.98 (0.83–1.15) | 0.764 | 0.88 (0.73–1.05) | 0.165 |
| Cardiovascular mortality | | | | | | | | | | | |
| ln CALLY (per-SD) | 5,046 | 382 (7.6) | 65,358.3 | 0.66 (0.60–0.73) | <0.001 | 0.81 (0.73–0.89) | <0.001 | 0.84 (0.75–0.93) | 0.001 | 0.84 (0.75–0.93) | 0.001 |
| Q1 (reference) | 778 | 94 (12.1) | 9,140.1 | 1.00 (Ref) |  | 1.00 (Ref) |  | 1.00 (Ref) |  | 1.00 (Ref) |  |
| Q2 | 1,059 | 110 (10.4) | 13,407.7 | 0.79 (0.60–1.04) | 0.097 | 0.76 (0.58–1.01) | 0.058 | 0.78 (0.59–1.04) | 0.09 | 0.81 (0.61–1.08) | 0.155 |
| Q3 | 1,341 | 97 (7.2) | 17,560.5 | 0.53 (0.40–0.71) | <0.001 | 0.60 (0.45–0.80) | <0.001 | 0.65 (0.49–0.87) | 0.004 | 0.68 (0.50–0.91) | 0.009 |
| Q4 | 1,868 | 81 (4.3) | 25,250.1 | 0.31 (0.23–0.41) | <0.001 | 0.53 (0.39–0.72) | <0.001 | 0.59 (0.44–0.80) | 0.001 | 0.58 (0.42–0.79) | 0.001 |
| ln RAR (per-SD) | 5,046 | 382 (7.6) | 65,358.3 | 1.62 (1.50–1.74) | <0.001 | 1.36 (1.22–1.51) | <0.001 | 1.33 (1.19–1.49) | <0.001 | 1.29 (1.15–1.45) | <0.001 |
| Q1 (reference) | 1,701 | 62 (3.6) | 24,615.2 | 1.00 (Ref) |  | 1.00 (Ref) |  | 1.00 (Ref) |  | 1.00 (Ref) |  |
| Q2 | 1,227 | 90 (7.3) | 16,098.2 | 2.33 (1.68–3.21) | <0.001 | 1.34 (0.97–1.86) | 0.08 | 1.31 (0.95–1.83) | 0.104 | 1.31 (0.94–1.82) | 0.113 |
| Q3 | 1,270 | 121 (9.5) | 15,460.3 | 3.37 (2.48–4.59) | <0.001 | 1.48 (1.08–2.03) | 0.015 | 1.38 (1.00–1.90) | 0.047 | 1.35 (0.98–1.87) | 0.066 |
| Q4 | 848 | 109 (12.9) | 9,184.8 | 5.26 (3.84–7.20) | <0.001 | 2.12 (1.52–2.95) | <0.001 | 1.97 (1.42–2.75) | <0.001 | 1.91 (1.35–2.70) | <0.001 |
| ln BAR (per-SD) | 5,046 | 382 (7.6) | 65,358.3 | 1.82 (1.65–2.02) | <0.001 | 1.13 (1.01–1.26) | 0.036 | 1.16 (1.03–1.29) | 0.01 | 1.11 (0.97–1.27) | 0.134 |
| Q1 (reference) | 1,320 | 52 (3.9) | 17,622.7 | 1.00 (Ref) |  | 1.00 (Ref) |  | 1.00 (Ref) |  | 1.00 (Ref) |  |
| Q2 | 1,223 | 62 (5.1) | 16,716.0 | 1.25 (0.86–1.80) | 0.243 | 0.89 (0.62–1.30) | 0.556 | 0.94 (0.65–1.37) | 0.753 | 0.96 (0.66–1.39) | 0.817 |
| Q3 | 1,285 | 92 (7.2) | 17,065.5 | 1.82 (1.30–2.56) | 0.001 | 0.95 (0.67–1.35) | 0.789 | 1.03 (0.73–1.46) | 0.865 | 1.03 (0.72–1.48) | 0.873 |
| Q4 | 1,218 | 176 (14.4) | 13,954.2 | 4.43 (3.25–6.04) | <0.001 | 1.29 (0.93–1.80) | 0.121 | 1.41 (1.01–1.96) | 0.043 | 1.33 (0.92–1.91) | 0.126 |

**Notes:** Analyses were restricted to participants with hypertension as the only PHF component. Participants with obesity, diabetes mellitus, and ASCVD were excluded from this isolated phenotype-specific sensitivity analysis. Hazard ratios (HRs) and 95% confidence intervals (CIs) were estimated using Cox proportional hazards regression models. CALLY, RAR, and BAR were natural log-transformed before analysis. HRs for continuous variables are presented per 1-standard deviation (SD) increase in each ln-transformed index. Model 1 was unadjusted. Model 2 was adjusted for age, sex, race/ethnicity, marital status, poverty-to-income ratio, and educational level. Model 3 was additionally adjusted for smoking status, alcohol consumption, and physical activity. Model 4 was further adjusted for the prespecified clinical and laboratory covariates that retained variability within the restricted sample. Phenotype-defining variables with no variation in the restricted sample were omitted from the model.

**Abbreviations:** BAR, blood urea nitrogen–albumin ratio; CALLY, C-reactive protein–albumin–lymphocyte index; CI, confidence interval; HR, hazard ratio; RAR, red cell distribution width–albumin ratio; SD, standard deviation.

**Supplementary Table S10. Associations of ln CALLY, ln RAR, and ln BAR with all-cause and cardiovascular mortality among adults with diabetes mellitus as the only PHF component**

| Characteristics | Participants, n | Events, n (%) | Person-years | Model 1 | | Model 2 | | Model 3 | | Model 4 | |
| --- | --- | --- | --- | --- | --- | --- | --- | --- | --- | --- | --- |
|  |  |  |  | HR (95% CI) | P value | HR (95% CI) | P value | HR (95% CI) | P value | HR (95% CI) | P value |
| All-cause mortality |  |  |  |  |  |  |  |  |  |  |  |
| ln CALLY (per SD) | 304 | 82 (27) | 3761.3 | 0.70 (0.58–0.84) | <0.001 | 0.84 (0.66–1.06) | 0.141 | 0.81 (0.64–1.03) | 0.087 | 0.78 (0.62–0.99) | 0.042 |
| Q1 | 69 | 29 (42) | 826.3 | 1.00 (Ref) |  | 1.00 (Ref) |  | 1.00 (Ref) |  | 1.00 (Ref) |  |
| Q2 | 57 | 19 (33.3) | 700.2 | 0.75 (0.42–1.34) | 0.336 | 0.63 (0.33–1.20) | 0.158 | 0.61 (0.32–1.18) | 0.144 | 0.67 (0.33–1.32) | 0.245 |
| Q3 | 74 | 15 (20.3) | 942.1 | 0.46 (0.24–0.85) | 0.014 | 0.59 (0.30–1.16) | 0.127 | 0.43 (0.21–0.90) | 0.025 | 0.41 (0.20–0.86) | 0.018 |
| Q4 | 104 | 19 (18.3) | 1292.7 | 0.43 (0.24–0.76) | 0.004 | 0.74 (0.39–1.42) | 0.369 | 0.66 (0.34–1.27) | 0.210 | 0.56 (0.28–1.12) | 0.102 |
| ln RAR (per SD) | 304 | 82 (27) | 3761.3 | 1.89 (1.50–2.37) | <0.001 | 1.41 (1.10–1.81) | 0.006 | 1.39 (1.09–1.78) | 0.008 | 1.54 (1.14–2.08) | 0.005 |
| Q1 | 99 | 15 (15.2) | 1355.3 | 1.00 (Ref) |  | 1.00 (Ref) |  | 1.00 (Ref) |  | 1.00 (Ref) |  |
| Q2 | 79 | 20 (25.3) | 980.4 | 1.86 (0.95–3.63) | 0.070 | 1.03 (0.49–2.17) | 0.929 | 0.97 (0.46–2.07) | 0.941 | 1.11 (0.52–2.39) | 0.782 |
| Q3 | 67 | 22 (32.8) | 813.3 | 2.53 (1.31–4.88) | 0.006 | 1.45 (0.71–2.98) | 0.309 | 1.58 (0.77–3.23) | 0.215 | 1.77 (0.84–3.71) | 0.133 |
| Q4 | 59 | 25 (42.4) | 612.2 | 3.85 (2.03–7.33) | <0.001 | 1.60 (0.79–3.21) | 0.189 | 1.58 (0.78–3.21) | 0.202 | 2.07 (0.93–4.63) | 0.075 |
| ln BAR (per SD) | 304 | 82 (27) | 3761.3 | 2.06 (1.59–2.67) | <0.001 | 1.30 (0.94–1.79) | 0.113 | 1.35 (0.98–1.85) | 0.065 | 1.51 (1.05–2.16) | 0.027 |
| Q1 | 62 | 8 (12.9) | 797.2 | 1.00 (Ref) |  | 1.00 (Ref) |  | 1.00 (Ref) |  | 1.00 (Ref) |  |
| Q2 | 81 | 15 (18.5) | 1060.8 | 1.39 (0.59–3.29) | 0.449 | 1.59 (0.65–3.89) | 0.307 | 1.80 (0.72–4.48) | 0.206 | 1.63 (0.64–4.13) | 0.303 |
| Q3 | 89 | 25 (28.1) | 1068.5 | 2.36 (1.07–5.24) | 0.034 | 1.10 (0.47–2.57) | 0.834 | 1.36 (0.56–3.28) | 0.495 | 1.17 (0.48–2.89) | 0.727 |
| Q4 | 72 | 34 (47.2) | 834.8 | 4.07 (1.88–8.80) | <0.001 | 1.23 (0.53–2.87) | 0.629 | 1.47 (0.62–3.48) | 0.385 | 1.55 (0.62–3.89) | 0.351 |
| Cardiovascular mortality |  |  |  |  |  |  |  |  |  |  |  |
| ln CALLY (per SD) | 304 | 23 (7.6) | 3761.3 | 0.68 (0.48–0.97) | 0.034 | 0.87 (0.55–1.36) | 0.530 | 0.85 (0.53–1.36) | 0.499 | 0.91 (0.54–1.52) | 0.711 |
| Q1 | 69 | 9 (13) | 826.3 | 1.00 (Ref) |  | 1.00 (Ref) |  | 1.00 (Ref) |  | 1.00 (Ref) |  |
| Q2 | 57 | 5 (8.8) | 700.2 | 0.66 (0.22–1.96) | 0.450 | 0.66 (0.19–2.28) | 0.515 | 0.62 (0.17–2.27) | 0.468 | 0.74 (0.19–2.95) | 0.674 |
| Q3 | 74 | 6 (8.1) | 942.1 | 0.59 (0.21–1.66) | 0.316 | 0.94 (0.28–3.08) | 0.913 | 1.07 (0.27–4.20) | 0.919 | 1.31 (0.32–5.36) | 0.707 |
| Q4 | 104 | 3 (2.9) | 1292.7 | 0.22 (0.06–0.81) | 0.022 | 0.46 (0.11–1.98) | 0.297 | 0.45 (0.10–2.00) | 0.294 | 0.30 (0.05–1.80) | 0.186 |
| ln RAR (per SD) | 304 | 23 (7.6) | 3761.3 | 1.97 (1.29–3.02) | 0.002 | 1.42 (0.87–2.31) | 0.160 | 1.39 (0.86–2.25) | 0.182 | 0.98 (0.49–1.98) | 0.957 |
| Q1 | 99 | 5 (5.1) | 1355.3 | 1.00 (Ref) |  | 1.00 (Ref) |  | 1.00 (Ref) |  | 1.00 (Ref) |  |
| Q2 | 79 | 5 (6.3) | 980.4 | 1.38 (0.40–4.77) | 0.610 | 0.69 (0.17–2.80) | 0.603 | 0.64 (0.15–2.66) | 0.538 | 0.44 (0.09–2.15) | 0.308 |
| Q3 | 67 | 4 (6) | 813.3 | 1.33 (0.36–4.97) | 0.670 | 0.58 (0.13–2.63) | 0.481 | 0.54 (0.11–2.54) | 0.433 | 0.21 (0.03–1.48) | 0.116 |
| Q4 | 59 | 9 (15.3) | 612.2 | 3.99 (1.33–11.94) | 0.013 | 1.36 (0.38–4.94) | 0.638 | 1.30 (0.36–4.73) | 0.687 | 0.35 (0.05–2.23) | 0.266 |
| ln BAR (per SD) | 304 | 23 (7.6) | 3761.3 | 2.97 (1.84–4.82) | <0.001 | 1.84 (0.94–3.60) | 0.077 | 1.78 (0.89–3.53) | 0.101 | 1.82 (0.80–4.12) | 0.153 |
| Q1 | 62 | 2 (3.2) | 797.2 | 1.00 (Ref) |  | 1.00 (Ref) |  | 1.00 (Ref) |  | 1.00 (Ref) |  |
| Q2 | 81 | 3 (3.7) | 1060.8 | 1.11 (0.19–6.64) | 0.909 | 0.88 (0.14–5.67) | 0.890 | 0.75 (0.11–5.01) | 0.764 | 0.62 (0.08–4.73) | 0.643 |
| Q3 | 89 | 5 (5.6) | 1068.5 | 1.87 (0.36–9.64) | 0.454 | 0.77 (0.14–4.36) | 0.769 | 0.63 (0.10–3.79) | 0.610 | 0.59 (0.09–4.07) | 0.594 |
| Q4 | 72 | 13 (18.1) | 834.8 | 6.15 (1.39–27.28) | 0.017 | 1.20 (0.23–6.26) | 0.828 | 0.96 (0.17–5.43) | 0.963 | 0.77 (0.11–5.43) | 0.797 |

**Notes:** Analyses were restricted to participants with diabetes mellitus as the only PHF component. Participants with obesity, hypertension, and ASCVD were excluded from this isolated phenotype-specific sensitivity analysis. Hazard ratios (HRs) and 95% confidence intervals (CIs) were estimated using Cox proportional hazards regression models. CALLY, RAR, and BAR were natural log-transformed before analysis. HRs for continuous variables are presented per 1-standard deviation (SD) increase in each ln-transformed index. Model 1 was unadjusted. Model 2 was adjusted for age, sex, race/ethnicity, marital status, poverty-to-income ratio, and educational level. Model 3 was additionally adjusted for smoking status, alcohol consumption, and physical activity. Model 4 was further adjusted for the prespecified clinical and laboratory covariates that retained variability within the restricted sample. Phenotype-defining variables with no variation in the restricted sample were omitted from the model. Estimates for cardiovascular mortality should be interpreted cautiously because of the limited number of events.

**Abbreviations:** BAR, blood urea nitrogen–albumin ratio; CALLY, C-reactive protein–albumin–lymphocyte index; CI, confidence interval; HR, hazard ratio; RAR, red cell distribution width–albumin ratio; SD, standard deviation.

**Supplementary Table S11. Associations of ln CALLY, ln RAR, and ln BAR with all-cause and cardiovascular mortality among adults with ASCVD as the only PHF component**

| Characteristics | Participants, n | Events, n (%) | Person-years | Model 1 | | Model 2 | | Model 3 | | Model 4 | |
| --- | --- | --- | --- | --- | --- | --- | --- | --- | --- | --- | --- |
|  |  |  |  | HR (95% CI) | P value | HR (95% CI) | P value | HR (95% CI) | P value | HR (95% CI) | P value |
| All-cause mortality |  |  |  |  |  |  |  |  |  |  |  |
| ln CALLY (per SD) | 195 | 80 (41) | 2241.2 | 0.78 (0.63–0.96) | 0.020 | 0.79 (0.63–0.99) | 0.039 | 0.77 (0.60–0.99) | 0.044 | 0.76 (0.58–1.00) | 0.047 |
| Q1 | 26 | 16 (61.5) | 292.8 | 1.00 (Ref) |  | 1.00 (Ref) |  | 1.00 (Ref) |  | 1.00 (Ref) |  |
| Q2 | 43 | 16 (37.2) | 515.5 | 0.57 (0.28–1.13) | 0.109 | 0.83 (0.39–1.76) | 0.630 | 0.70 (0.31–1.60) | 0.399 | 0.69 (0.27–1.73) | 0.424 |
| Q3 | 65 | 31 (47.7) | 668.9 | 0.87 (0.48–1.59) | 0.653 | 0.85 (0.43–1.69) | 0.642 | 0.82 (0.38–1.77) | 0.615 | 0.88 (0.38–2.04) | 0.762 |
| Q4 | 61 | 17 (27.9) | 763.9 | 0.41 (0.21–0.81) | 0.010 | 0.43 (0.21–0.88) | 0.022 | 0.39 (0.17–0.88) | 0.024 | 0.35 (0.14–0.87) | 0.024 |
| ln RAR (per SD) | 195 | 80 (41) | 2241.2 | 1.48 (1.25–1.76) | <0.001 | 1.41 (1.13–1.75) | 0.002 | 1.44 (1.15–1.80) | 0.002 | 1.52 (1.16–2.00) | 0.003 |
| Q1 | 55 | 15 (27.3) | 753.2 | 1.00 (Ref) |  | 1.00 (Ref) |  | 1.00 (Ref) |  | 1.00 (Ref) |  |
| Q2 | 35 | 13 (37.1) | 405.7 | 1.71 (0.81–3.61) | 0.161 | 1.11 (0.51–2.42) | 0.799 | 1.02 (0.46–2.29) | 0.952 | 1.01 (0.43–2.41) | 0.973 |
| Q3 | 63 | 27 (42.9) | 713.3 | 2.08 (1.10–3.93) | 0.025 | 1.16 (0.58–2.34) | 0.672 | 1.00 (0.49–2.06) | 0.995 | 0.82 (0.37–1.79) | 0.616 |
| Q4 | 42 | 25 (59.5) | 369.0 | 3.92 (2.04–7.56) | <0.001 | 3.45 (1.65–7.23) | 0.001 | 3.85 (1.79–8.29) | 0.001 | 3.87 (1.65–9.04) | 0.002 |
| ln BAR (per SD) | 195 | 80 (41) | 2241.2 | 1.81 (1.46–2.26) | <0.001 | 1.21 (0.91–1.60) | 0.191 | 1.24 (0.92–1.66) | 0.153 | 1.41 (0.98–2.01) | 0.063 |
| Q1 | 38 | 4 (10.5) | 482.9 | 1.00 (Ref) |  | 1.00 (Ref) |  | 1.00 (Ref) |  | 1.00 (Ref) |  |
| Q2 | 35 | 12 (34.3) | 391.9 | 3.73 (1.20–11.57) | 0.023 | 1.98 (0.58–6.78) | 0.277 | 2.09 (0.61–7.23) | 0.243 | 1.91 (0.52–7.06) | 0.332 |
| Q3 | 54 | 25 (46.3) | 660.3 | 4.57 (1.59–13.14) | 0.005 | 1.08 (0.33–3.51) | 0.893 | 1.20 (0.37–3.95) | 0.763 | 1.25 (0.35–4.41) | 0.731 |
| Q4 | 68 | 39 (57.4) | 706.0 | 6.76 (2.41–18.92) | <0.001 | 1.64 (0.52–5.12) | 0.395 | 1.82 (0.57–5.77) | 0.311 | 2.02 (0.56–7.29) | 0.281 |
| Cardiovascular mortality |  |  |  |  |  |  |  |  |  |  |  |
| ln CALLY (per SD) | 195 | 21 (10.8) | 2241.2 | 1.01 (0.66–1.56) | 0.948 | 1.02 (0.64–1.62) | 0.927 | 1.06 (0.64–1.74) | 0.826 | 1.07 (0.60–1.92) | 0.812 |
| Q1 | 26 | 3 (11.5) | 292.8 | 1.00 (Ref) |  | 1.00 (Ref) |  | 1.00 (Ref) |  | 1.00 (Ref) |  |
| Q2 | 43 | 2 (4.7) | 515.5 | 0.38 (0.06–2.26) | 0.286 | 0.52 (0.08–3.47) | 0.501 | 0.49 (0.06–3.88) | 0.495 | 0.45 (0.05–4.00) | 0.471 |
| Q3 | 65 | 8 (12.3) | 668.9 | 1.16 (0.31–4.36) | 0.831 | 0.90 (0.19–4.27) | 0.891 | 0.93 (0.17–4.94) | 0.927 | 0.45 (0.07–2.96) | 0.406 |
| Q4 | 61 | 8 (13.1) | 763.9 | 1.00 (0.26–3.76) | 0.997 | 1.06 (0.25–4.48) | 0.932 | 1.23 (0.24–6.29) | 0.800 | 1.23 (0.19–7.96) | 0.827 |
| ln RAR (per SD) | 195 | 21 (10.8) | 2241.2 | 1.31 (0.90–1.92) | 0.161 | 1.07 (0.65–1.76) | 0.801 | 1.09 (0.65–1.83) | 0.748 | 1.27 (0.60–2.72) | 0.534 |
| Q1 | 55 | 5 (9.1) | 753.2 | 1.00 (Ref) |  | 1.00 (Ref) |  | 1.00 (Ref) |  | 1.00 (Ref) |  |
| Q2 | 35 | 4 (11.4) | 405.7 | 1.77 (0.46–6.81) | 0.403 | 1.18 (0.25–5.60) | 0.831 | 1.23 (0.25–6.04) | 0.798 | 0.85 (0.14–5.23) | 0.858 |
| Q3 | 63 | 5 (7.9) | 713.3 | 1.28 (0.36–4.54) | 0.705 | 0.45 (0.10–2.04) | 0.302 | 0.43 (0.09–1.94) | 0.270 | 0.20 (0.03–1.20) | 0.078 |
| Q4 | 42 | 7 (16.7) | 369.0 | 3.81 (1.13–12.89) | 0.031 | 3.32 (0.78–14.19) | 0.106 | 4.49 (0.99–20.36) | 0.051 | 6.70 (0.94–47.63) | 0.057 |
| ln BAR (per SD) | 195 | 21 (10.8) | 2241.2 | 1.95 (1.28–2.99) | 0.002 | 1.51 (0.85–2.68) | 0.162 | 1.52 (0.85–2.73) | 0.160 | 2.33 (1.09–4.94) | 0.028 |
| Q1 | 38 | 1 (2.6) | 482.9 | 1.00 (Ref) |  | 1.00 (Ref) |  | 1.00 (Ref) |  | 1.00 (Ref) |  |
| Q2 | 35 | 3 (8.6) | 391.9 | 3.73 (0.39–35.85) | 0.255 | 3.02 (0.26–35.73) | 0.381 | 2.43 (0.20–30.25) | 0.489 | 2.61 (0.13–50.50) | 0.526 |
| Q3 | 54 | 7 (13) | 660.3 | 5.18 (0.64–42.13) | 0.124 | 1.37 (0.13–14.95) | 0.794 | 1.25 (0.11–13.74) | 0.857 | 1.54 (0.10–22.66) | 0.754 |
| Q4 | 68 | 10 (14.7) | 706.0 | 7.00 (0.90–54.77) | 0.064 | 2.49 (0.24–25.49) | 0.443 | 2.29 (0.23–22.58) | 0.479 | 5.39 (0.38–75.58) | 0.211 |

**Notes:** Analyses were restricted to participants with ASCVD as the only PHF component. Participants with obesity, hypertension, and diabetes mellitus were excluded from this isolated phenotype-specific sensitivity analysis. Hazard ratios (HRs) and 95% confidence intervals (CIs) were estimated using Cox proportional hazards regression models. CALLY, RAR, and BAR were natural log-transformed before analysis. HRs for continuous variables are presented per 1-standard deviation (SD) increase in each ln-transformed index. Model 1 was unadjusted. Model 2 was adjusted for age, sex, race/ethnicity, marital status, poverty-to-income ratio, and educational level. Model 3 was additionally adjusted for smoking status, alcohol consumption, and physical activity. Model 4 was further adjusted for the prespecified clinical and laboratory covariates that retained variability within the restricted sample. Phenotype-defining variables with no variation in the restricted sample were omitted from the model. Estimates for cardiovascular mortality should be interpreted cautiously because of the limited number of events.

**Abbreviations:** BAR, blood urea nitrogen–albumin ratio; CALLY, C-reactive protein–albumin–lymphocyte index; CI, confidence interval; HR, hazard ratio; RAR, red cell distribution width–albumin ratio; SD, standard deviation.

**Supplementary Table S12. Associations of ln CALLY, ln RAR, and ln BAR with all-cause and cardiovascular mortality among adults with one PHF component**

| Characteristics | Participants, n | Events, n (%) | Person-years | Model 1 | | Model 2 | | Model 3 | | Model 4 | |
| --- | --- | --- | --- | --- | --- | --- | --- | --- | --- | --- | --- |
|  |  |  |  | HR (95% CI) | P value | HR (95% CI) | P value | HR (95% CI) | P value | HR (95% CI) | P value |
| All-cause mortality |  |  |  |  |  |  |  |  |  |  |  |
| ln CALLY (per SD) | 7752 | 1619 (20.9) | 101952.9 | 0.77 (0.74–0.81) | <0.001 | 0.81 (0.77–0.86) | <0.001 | 0.84 (0.79–0.88) | <0.001 | 0.83 (0.79–0.87) | <0.001 |
| Q1 | 1525 | 412 (27) | 19197.3 | 1.00 (Ref) |  | 1.00 (Ref) |  | 1.00 (Ref) |  | 1.00 (Ref) |  |
| Q2 | 1819 | 434 (23.9) | 23977.4 | 0.84 (0.73–0.96) | 0.010 | 0.77 (0.68–0.89) | <0.001 | 0.79 (0.69–0.90) | 0.001 | 0.81 (0.71–0.93) | 0.003 |
| Q3 | 2015 | 412 (20.4) | 26539.2 | 0.72 (0.63–0.83) | <0.001 | 0.64 (0.56–0.73) | <0.001 | 0.67 (0.59–0.77) | <0.001 | 0.69 (0.60–0.80) | <0.001 |
| Q4 | 2393 | 361 (15.1) | 32239.0 | 0.52 (0.45–0.60) | <0.001 | 0.58 (0.51–0.67) | <0.001 | 0.63 (0.54–0.72) | <0.001 | 0.60 (0.52–0.70) | <0.001 |
| ln RAR (per SD) | 7752 | 1619 (20.9) | 101952.9 | 1.44 (1.39–1.50) | <0.001 | 1.40 (1.33–1.47) | <0.001 | 1.37 (1.31–1.44) | <0.001 | 1.34 (1.27–1.41) | <0.001 |
| Q1 | 2334 | 292 (12.5) | 33650.0 | 1.00 (Ref) |  | 1.00 (Ref) |  | 1.00 (Ref) |  | 1.00 (Ref) |  |
| Q2 | 1833 | 348 (19) | 24422.6 | 1.69 (1.45–1.98) | <0.001 | 1.13 (0.96–1.32) | 0.132 | 1.10 (0.94–1.29) | 0.247 | 1.11 (0.95–1.30) | 0.190 |
| Q3 | 2067 | 504 (24.4) | 26153.1 | 2.34 (2.02–2.70) | <0.001 | 1.34 (1.16–1.56) | <0.001 | 1.27 (1.09–1.47) | 0.002 | 1.27 (1.09–1.48) | 0.002 |
| Q4 | 1518 | 475 (31.3) | 17727.2 | 3.31 (2.86–3.83) | <0.001 | 2.00 (1.71–2.33) | <0.001 | 1.86 (1.60–2.18) | <0.001 | 1.80 (1.53–2.13) | <0.001 |
| ln BAR (per SD) | 7752 | 1619 (20.9) | 101952.9 | 1.81 (1.72–1.91) | <0.001 | 1.02 (0.97–1.08) | 0.442 | 1.05 (1.00–1.11) | 0.074 | 1.01 (0.94–1.07) | 0.856 |
| Q1 | 2203 | 286 (13) | 29629.6 | 1.00 (Ref) |  | 1.00 (Ref) |  | 1.00 (Ref) |  | 1.00 (Ref) |  |
| Q2 | 2026 | 281 (13.9) | 27781.5 | 1.04 (0.88–1.23) | 0.642 | 0.76 (0.65–0.90) | 0.001 | 0.80 (0.68–0.95) | 0.010 | 0.81 (0.68–0.95) | 0.011 |
| Q3 | 1925 | 404 (21) | 25709.0 | 1.62 (1.40–1.89) | <0.001 | 0.76 (0.65–0.89) | 0.001 | 0.81 (0.69–0.95) | 0.008 | 0.81 (0.69–0.95) | 0.009 |
| Q4 | 1598 | 648 (40.6) | 18832.8 | 3.66 (3.18–4.21) | <0.001 | 0.91 (0.79–1.06) | 0.233 | 0.98 (0.85–1.14) | 0.829 | 0.92 (0.78–1.08) | 0.304 |
| Cardiovascular mortality |  |  |  |  |  |  |  |  |  |  |  |
| ln CALLY (per SD) | 7752 | 451 (5.8) | 101952.9 | 0.75 (0.69–0.83) | <0.001 | 0.80 (0.73–0.88) | <0.001 | 0.82 (0.75–0.91) | <0.001 | 0.82 (0.75–0.91) | <0.001 |
| Q1 | 1525 | 119 (7.8) | 19197.3 | 1.00 (Ref) |  | 1.00 (Ref) |  | 1.00 (Ref) |  | 1.00 (Ref) |  |
| Q2 | 1819 | 125 (6.9) | 23977.4 | 0.83 (0.65–1.07) | 0.157 | 0.76 (0.59–0.98) | 0.031 | 0.77 (0.60–1.00) | 0.047 | 0.79 (0.61–1.03) | 0.082 |
| Q3 | 2015 | 114 (5.7) | 26539.2 | 0.69 (0.53–0.89) | 0.005 | 0.61 (0.47–0.79) | <0.001 | 0.64 (0.49–0.84) | 0.001 | 0.66 (0.50–0.86) | 0.002 |
| Q4 | 2393 | 93 (3.9) | 32239.0 | 0.46 (0.35–0.61) | <0.001 | 0.53 (0.41–0.70) | <0.001 | 0.57 (0.44–0.76) | <0.001 | 0.57 (0.43–0.76) | <0.001 |
| ln RAR (per SD) | 7752 | 451 (5.8) | 101952.9 | 1.43 (1.33–1.54) | <0.001 | 1.34 (1.21–1.48) | <0.001 | 1.32 (1.19–1.46) | <0.001 | 1.28 (1.15–1.42) | <0.001 |
| Q1 | 2334 | 74 (3.2) | 33650.0 | 1.00 (Ref) |  | 1.00 (Ref) |  | 1.00 (Ref) |  | 1.00 (Ref) |  |
| Q2 | 1833 | 105 (5.7) | 24422.6 | 2.03 (1.51–2.73) | <0.001 | 1.25 (0.93–1.69) | 0.145 | 1.24 (0.92–1.68) | 0.158 | 1.25 (0.92–1.69) | 0.158 |
| Q3 | 2067 | 137 (6.6) | 26153.1 | 2.54 (1.91–3.37) | <0.001 | 1.33 (0.99–1.78) | 0.057 | 1.26 (0.94–1.69) | 0.122 | 1.23 (0.91–1.66) | 0.171 |
| Q4 | 1518 | 135 (8.9) | 17727.2 | 3.76 (2.83–5.00) | <0.001 | 2.04 (1.51–2.75) | <0.001 | 1.92 (1.42–2.59) | <0.001 | 1.86 (1.35–2.55) | <0.001 |
| ln BAR (per SD) | 7752 | 451 (5.8) | 101952.9 | 2.07 (1.89–2.28) | <0.001 | 1.13 (1.02–1.25) | 0.025 | 1.16 (1.04–1.28) | 0.007 | 1.12 (0.99–1.27) | 0.063 |
| Q1 | 2203 | 60 (2.7) | 29629.6 | 1.00 (Ref) |  | 1.00 (Ref) |  | 1.00 (Ref) |  | 1.00 (Ref) |  |
| Q2 | 2026 | 72 (3.6) | 27781.5 | 1.27 (0.90–1.79) | 0.175 | 0.91 (0.65–1.29) | 0.605 | 0.96 (0.68–1.35) | 0.797 | 0.97 (0.69–1.38) | 0.871 |
| Q3 | 1925 | 109 (5.7) | 25709.0 | 2.08 (1.52–2.85) | <0.001 | 0.94 (0.68–1.29) | 0.688 | 1.00 (0.73–1.39) | 0.988 | 1.01 (0.73–1.41) | 0.937 |
| Q4 | 1598 | 210 (13.1) | 18832.8 | 5.65 (4.24–7.53) | <0.001 | 1.30 (0.96–1.76) | 0.090 | 1.40 (1.03–1.91) | 0.030 | 1.37 (0.98–1.91) | 0.067 |

**Notes:** Analyses were restricted to participants with one PHF component. Hazard ratios (HRs) and 95% confidence intervals (CIs) were estimated using Cox proportional hazards regression models. CALLY, RAR, and BAR were natural log-transformed before analysis. HRs for continuous variables are presented per 1-standard deviation (SD) increase in each ln-transformed index. Model 1 was unadjusted. Model 2 was adjusted for age, sex, race/ethnicity, marital status, poverty-to-income ratio, and educational level. Model 3 was additionally adjusted for smoking status, alcohol consumption, and physical activity. Model 4 was further adjusted for body mass index, stroke, angina, hypertension, hyperlipidemia, diabetes mellitus, alanine aminotransferase, aspartate aminotransferase, total bilirubin, estimated glomerular filtration rate, hemoglobin, and cancer history.

**Abbreviations:** BAR, blood urea nitrogen–albumin ratio; CALLY, C-reactive protein–albumin–lymphocyte index; CI, confidence interval; HR, hazard ratio; RAR, red cell distribution width–albumin ratio; SD, standard deviation.

**Supplementary Table S13. Associations of ln CALLY, ln RAR, and ln BAR with all-cause and cardiovascular mortality among adults with two PHF components**

| Characteristics | Participants, n | Events, n (%) | Person-years | Model 1 | | Model 2 | | Model 3 | | Model 4 | |
| --- | --- | --- | --- | --- | --- | --- | --- | --- | --- | --- | --- |
|  |  |  |  | HR (95% CI) | P value | HR (95% CI) | P value | HR (95% CI) | P value | HR (95% CI) | P value |
| All-cause mortality |  |  |  |  |  |  |  |  |  |  |  |
| ln CALLY (per SD) | 4775 | 1252 (26.2) | 60438.5 | 0.93 (0.88–0.99) | 0.027 | 0.90 (0.84–0.95) | <0.001 | 0.90 (0.84–0.95) | <0.001 | 0.87 (0.82–0.93) | <0.001 |
| Q1 | 1384 | 408 (29.5) | 17198.8 | 1.00 (Ref) |  | 1.00 (Ref) |  | 1.00 (Ref) |  | 1.00 (Ref) |  |
| Q2 | 1258 | 320 (25.4) | 16183.8 | 0.83 (0.71–0.96) | 0.011 | 0.83 (0.72–0.97) | 0.016 | 0.83 (0.72–0.96) | 0.013 | 0.82 (0.71–0.95) | 0.008 |
| Q3 | 1191 | 274 (23) | 15382.5 | 0.75 (0.64–0.87) | <0.001 | 0.71 (0.61–0.83) | <0.001 | 0.69 (0.59–0.81) | <0.001 | 0.67 (0.57–0.78) | <0.001 |
| Q4 | 942 | 250 (26.5) | 11673.5 | 0.91 (0.78–1.07) | 0.241 | 0.82 (0.70–0.96) | 0.016 | 0.84 (0.71–0.98) | 0.031 | 0.74 (0.63–0.88) | 0.001 |
| ln RAR (per SD) | 4775 | 1252 (26.2) | 60438.5 | 1.30 (1.24–1.37) | <0.001 | 1.23 (1.17–1.30) | <0.001 | 1.22 (1.16–1.29) | <0.001 | 1.22 (1.15–1.28) | <0.001 |
| Q1 | 1037 | 201 (19.4) | 14330.3 | 1.00 (Ref) |  | 1.00 (Ref) |  | 1.00 (Ref) |  | 1.00 (Ref) |  |
| Q2 | 1044 | 261 (25) | 13761.2 | 1.38 (1.15–1.66) | 0.001 | 1.15 (0.96–1.39) | 0.138 | 1.13 (0.94–1.36) | 0.188 | 1.19 (0.98–1.43) | 0.075 |
| Q3 | 1370 | 353 (25.8) | 17113.2 | 1.54 (1.29–1.83) | <0.001 | 1.17 (0.98–1.39) | 0.090 | 1.13 (0.95–1.36) | 0.162 | 1.21 (1.01–1.45) | 0.040 |
| Q4 | 1324 | 437 (33) | 15233.7 | 2.18 (1.84–2.57) | <0.001 | 1.65 (1.39–1.97) | <0.001 | 1.61 (1.35–1.92) | <0.001 | 1.72 (1.42–2.07) | <0.001 |
| ln BAR (per SD) | 4775 | 1252 (26.2) | 60438.5 | 1.83 (1.72–1.94) | <0.001 | 1.17 (1.09–1.24) | <0.001 | 1.19 (1.11–1.27) | <0.001 | 1.16 (1.08–1.25) | <0.001 |
| Q1 | 1094 | 160 (14.6) | 14222.9 | 1.00 (Ref) |  | 1.00 (Ref) |  | 1.00 (Ref) |  | 1.00 (Ref) |  |
| Q2 | 1186 | 237 (20) | 15526.8 | 1.35 (1.10–1.65) | 0.004 | 0.92 (0.75–1.13) | 0.441 | 0.98 (0.80–1.21) | 0.872 | 1.02 (0.83–1.25) | 0.837 |
| Q3 | 1275 | 311 (24.4) | 16879.3 | 1.62 (1.34–1.96) | <0.001 | 0.84 (0.69–1.02) | 0.076 | 0.91 (0.75–1.11) | 0.340 | 0.94 (0.77–1.14) | 0.526 |
| Q4 | 1220 | 544 (44.6) | 13809.4 | 3.58 (3.00–4.28) | <0.001 | 1.14 (0.95–1.38) | 0.156 | 1.22 (1.01–1.47) | 0.037 | 1.18 (0.96–1.45) | 0.107 |
| Cardiovascular mortality |  |  |  |  |  |  |  |  |  |  |  |
| ln CALLY (per SD) | 4775 | 410 (8.6) | 60438.5 | 0.95 (0.85–1.05) | 0.313 | 0.91 (0.82–1.01) | 0.084 | 0.91 (0.82–1.01) | 0.086 | 0.88 (0.79–0.98) | 0.019 |
| Q1 | 1384 | 124 (9) | 17198.8 | 1.00 (Ref) |  | 1.00 (Ref) |  | 1.00 (Ref) |  | 1.00 (Ref) |  |
| Q2 | 1258 | 112 (8.9) | 16183.8 | 0.95 (0.74–1.23) | 0.696 | 0.98 (0.75–1.26) | 0.852 | 0.97 (0.75–1.26) | 0.835 | 0.94 (0.73–1.23) | 0.668 |
| Q3 | 1191 | 94 (7.9) | 15382.5 | 0.84 (0.64–1.10) | 0.209 | 0.81 (0.62–1.06) | 0.130 | 0.80 (0.61–1.05) | 0.100 | 0.77 (0.58–1.02) | 0.066 |
| Q4 | 942 | 80 (8.5) | 11673.5 | 0.96 (0.72–1.27) | 0.775 | 0.88 (0.66–1.17) | 0.374 | 0.89 (0.67–1.19) | 0.429 | 0.77 (0.57–1.04) | 0.087 |
| ln RAR (per SD) | 4775 | 410 (8.6) | 60438.5 | 1.34 (1.23–1.45) | <0.001 | 1.25 (1.14–1.36) | <0.001 | 1.24 (1.13–1.36) | <0.001 | 1.25 (1.14–1.37) | <0.001 |
| Q1 | 1037 | 64 (6.2) | 14330.3 | 1.00 (Ref) |  | 1.00 (Ref) |  | 1.00 (Ref) |  | 1.00 (Ref) |  |
| Q2 | 1044 | 93 (8.9) | 13761.2 | 1.55 (1.13–2.14) | 0.007 | 1.26 (0.91–1.73) | 0.162 | 1.25 (0.90–1.72) | 0.178 | 1.32 (0.95–1.82) | 0.095 |
| Q3 | 1370 | 106 (7.7) | 17113.2 | 1.46 (1.07–1.99) | 0.017 | 1.04 (0.75–1.43) | 0.822 | 1.01 (0.74–1.39) | 0.942 | 1.11 (0.80–1.53) | 0.542 |
| Q4 | 1324 | 147 (11.1) | 15233.7 | 2.33 (1.73–3.12) | <0.001 | 1.64 (1.20–2.23) | 0.002 | 1.60 (1.18–2.18) | 0.003 | 1.76 (1.26–2.44) | 0.001 |
| ln BAR (per SD) | 4775 | 410 (8.6) | 60438.5 | 1.98 (1.79–2.18) | <0.001 | 1.21 (1.08–1.35) | 0.001 | 1.23 (1.10–1.37) | <0.001 | 1.23 (1.07–1.41) | 0.003 |
| Q1 | 1094 | 47 (4.3) | 14222.9 | 1.00 (Ref) |  | 1.00 (Ref) |  | 1.00 (Ref) |  | 1.00 (Ref) |  |
| Q2 | 1186 | 64 (5.4) | 15526.8 | 1.24 (0.85–1.80) | 0.272 | 0.80 (0.55–1.17) | 0.253 | 0.85 (0.58–1.25) | 0.405 | 0.88 (0.60–1.29) | 0.514 |
| Q3 | 1275 | 112 (8.8) | 16879.3 | 1.97 (1.40–2.77) | <0.001 | 0.93 (0.66–1.32) | 0.695 | 1.01 (0.71–1.43) | 0.962 | 1.04 (0.73–1.49) | 0.822 |
| Q4 | 1220 | 187 (15.3) | 13809.4 | 4.19 (3.04–5.77) | <0.001 | 1.17 (0.83–1.64) | 0.364 | 1.25 (0.89–1.75) | 0.198 | 1.25 (0.87–1.80) | 0.236 |

**Notes:** Analyses were restricted to participants with two PHF components. Hazard ratios (HRs) and 95% confidence intervals (CIs) were estimated using Cox proportional hazards regression models. CALLY, RAR, and BAR were natural log-transformed before analysis. HRs for continuous variables are presented per 1-standard deviation (SD) increase in each ln-transformed index. Model 1 was unadjusted. Model 2 was adjusted for age, sex, race/ethnicity, marital status, poverty-to-income ratio, and educational level. Model 3 was additionally adjusted for smoking status, alcohol consumption, and physical activity. Model 4 was further adjusted for body mass index, stroke, angina, hypertension, hyperlipidemia, diabetes mellitus, alanine aminotransferase, aspartate aminotransferase, total bilirubin, estimated glomerular filtration rate, hemoglobin, and cancer history.

**Abbreviations:** BAR, blood urea nitrogen–albumin ratio; CALLY, C-reactive protein–albumin–lymphocyte index; CI, confidence interval; HR, hazard ratio; RAR, red cell distribution width–albumin ratio; SD, standard deviation.

**Supplementary Table S14. Associations of ln CALLY, ln RAR, and ln BAR with all-cause and cardiovascular mortality among adults with three or more PHF components**

| Characteristics | Participants, n | Events, n (%) | Person-years | Model 1 | | Model 2 | | Model 3 | | Model 4 | |
| --- | --- | --- | --- | --- | --- | --- | --- | --- | --- | --- | --- |
|  |  |  |  | HR (95% CI) | P value | HR (95% CI) | P value | HR (95% CI) | P value | HR (95% CI) | P value |
| All-cause mortality |  |  |  |  |  |  |  |  |  |  |  |
| ln CALLY (per SD) | 2190 | 930 (42.5) | 24287.1 | 0.97 (0.90–1.04) | 0.393 | 0.85 (0.79–0.91) | <0.001 | 0.85 (0.79–0.91) | <0.001 | 0.86 (0.80–0.92) | <0.001 |
| Q1 | 764 | 334 (43.7) | 8437.6 | 1.00 (Ref) |  | 1.00 (Ref) |  | 1.00 (Ref) |  | 1.00 (Ref) |  |
| Q2 | 607 | 246 (40.5) | 6832.5 | 0.92 (0.78–1.09) | 0.335 | 0.77 (0.65–0.91) | 0.002 | 0.76 (0.64–0.90) | 0.001 | 0.79 (0.67–0.94) | 0.008 |
| Q3 | 474 | 197 (41.6) | 5176.8 | 0.98 (0.82–1.17) | 0.824 | 0.78 (0.65–0.94) | 0.007 | 0.80 (0.67–0.95) | 0.013 | 0.82 (0.68–0.98) | 0.033 |
| Q4 | 345 | 153 (44.3) | 3840.3 | 1.03 (0.85–1.24) | 0.782 | 0.69 (0.56–0.84) | <0.001 | 0.70 (0.57–0.85) | <0.001 | 0.69 (0.57–0.85) | <0.001 |
| ln RAR (per SD) | 2190 | 930 (42.5) | 24287.1 | 1.25 (1.18–1.32) | <0.001 | 1.38 (1.29–1.47) | <0.001 | 1.37 (1.29–1.46) | <0.001 | 1.33 (1.24–1.43) | <0.001 |
| Q1 | 291 | 101 (34.7) | 3683.5 | 1.00 (Ref) |  | 1.00 (Ref) |  | 1.00 (Ref) |  | 1.00 (Ref) |  |
| Q2 | 432 | 163 (37.7) | 5007.6 | 1.23 (0.96–1.58) | 0.100 | 1.22 (0.95–1.57) | 0.113 | 1.20 (0.94–1.55) | 0.148 | 1.15 (0.89–1.48) | 0.274 |
| Q3 | 618 | 259 (41.9) | 6929.2 | 1.44 (1.14–1.81) | 0.002 | 1.42 (1.12–1.79) | 0.003 | 1.36 (1.08–1.72) | 0.010 | 1.29 (1.02–1.64) | 0.036 |
| Q4 | 849 | 407 (47.9) | 8666.8 | 1.86 (1.49–2.31) | <0.001 | 1.97 (1.57–2.47) | <0.001 | 1.90 (1.51–2.39) | <0.001 | 1.72 (1.35–2.19) | <0.001 |
| ln BAR (per SD) | 2190 | 930 (42.5) | 24287.1 | 1.58 (1.49–1.68) | <0.001 | 1.25 (1.17–1.34) | <0.001 | 1.25 (1.17–1.34) | <0.001 | 1.16 (1.06–1.27) | 0.002 |
| Q1 | 352 | 87 (24.7) | 4157.5 | 1.00 (Ref) |  | 1.00 (Ref) |  | 1.00 (Ref) |  | 1.00 (Ref) |  |
| Q2 | 449 | 137 (30.5) | 5258.2 | 1.25 (0.95–1.63) | 0.107 | 1.05 (0.80–1.37) | 0.734 | 1.04 (0.79–1.36) | 0.784 | 1.00 (0.76–1.31) | 0.994 |
| Q3 | 498 | 183 (36.7) | 5961.0 | 1.45 (1.12–1.87) | 0.005 | 0.94 (0.72–1.22) | 0.625 | 0.96 (0.74–1.25) | 0.787 | 0.88 (0.68–1.15) | 0.361 |
| Q4 | 891 | 523 (58.7) | 8910.4 | 2.96 (2.36–3.71) | <0.001 | 1.35 (1.06–1.71) | 0.013 | 1.34 (1.06–1.70) | 0.015 | 1.10 (0.85–1.43) | 0.481 |
| Cardiovascular mortality |  |  |  |  |  |  |  |  |  |  |  |
| ln CALLY (per SD) | 2190 | 365 (16.7) | 24287.1 | 1.07 (0.96–1.20) | 0.209 | 0.92 (0.82–1.03) | 0.160 | 0.93 (0.83–1.04) | 0.195 | 0.93 (0.83–1.05) | 0.244 |
| Q1 | 764 | 123 (16.1) | 8437.6 | 1.00 (Ref) |  | 1.00 (Ref) |  | 1.00 (Ref) |  | 1.00 (Ref) |  |
| Q2 | 607 | 86 (14.2) | 6832.5 | 0.88 (0.67–1.16) | 0.356 | 0.72 (0.55–0.95) | 0.021 | 0.72 (0.54–0.95) | 0.021 | 0.74 (0.56–0.99) | 0.041 |
| Q3 | 474 | 87 (18.4) | 5176.8 | 1.18 (0.90–1.55) | 0.237 | 0.93 (0.70–1.22) | 0.589 | 0.94 (0.71–1.25) | 0.683 | 0.97 (0.73–1.30) | 0.846 |
| Q4 | 345 | 69 (20) | 3840.3 | 1.26 (0.94–1.69) | 0.123 | 0.81 (0.60–1.10) | 0.186 | 0.83 (0.61–1.13) | 0.230 | 0.82 (0.60–1.12) | 0.219 |
| ln RAR (per SD) | 2190 | 365 (16.7) | 24287.1 | 1.14 (1.04–1.26) | 0.007 | 1.26 (1.12–1.40) | <0.001 | 1.25 (1.12–1.40) | <0.001 | 1.18 (1.05–1.34) | 0.008 |
| Q1 | 291 | 49 (16.8) | 3683.5 | 1.00 (Ref) |  | 1.00 (Ref) |  | 1.00 (Ref) |  | 1.00 (Ref) |  |
| Q2 | 432 | 68 (15.7) | 5007.6 | 1.06 (0.74–1.53) | 0.747 | 1.00 (0.69–1.45) | 0.998 | 0.98 (0.68–1.43) | 0.927 | 0.99 (0.68–1.45) | 0.973 |
| Q3 | 618 | 98 (15.9) | 6929.2 | 1.12 (0.79–1.58) | 0.521 | 1.11 (0.78–1.57) | 0.561 | 1.08 (0.76–1.53) | 0.685 | 1.04 (0.72–1.49) | 0.847 |
| Q4 | 849 | 150 (17.7) | 8666.8 | 1.41 (1.02–1.95) | 0.037 | 1.45 (1.03–2.05) | 0.031 | 1.41 (1.00–1.99) | 0.049 | 1.30 (0.90–1.88) | 0.169 |
| ln BAR (per SD) | 2190 | 365 (16.7) | 24287.1 | 1.65 (1.50–1.81) | <0.001 | 1.26 (1.13–1.40) | <0.001 | 1.26 (1.13–1.41) | <0.001 | 1.23 (1.07–1.43) | 0.004 |
| Q1 | 352 | 31 (8.8) | 4157.5 | 1.00 (Ref) |  | 1.00 (Ref) |  | 1.00 (Ref) |  | 1.00 (Ref) |  |
| Q2 | 449 | 55 (12.2) | 5258.2 | 1.41 (0.91–2.19) | 0.128 | 1.15 (0.74–1.79) | 0.539 | 1.15 (0.74–1.79) | 0.534 | 1.14 (0.73–1.79) | 0.562 |
| Q3 | 498 | 69 (13.9) | 5961.0 | 1.53 (1.00–2.33) | 0.050 | 0.93 (0.60–1.43) | 0.736 | 0.96 (0.62–1.47) | 0.836 | 0.90 (0.58–1.40) | 0.653 |
| Q4 | 891 | 210 (23.6) | 8910.4 | 3.35 (2.30–4.88) | <0.001 | 1.33 (0.90–1.97) | 0.158 | 1.35 (0.91–2.00) | 0.139 | 1.19 (0.77–1.82) | 0.433 |

**Notes:** Analyses were restricted to participants with three or more PHF components. Hazard ratios (HRs) and 95% confidence intervals (CIs) were estimated using Cox proportional hazards regression models. CALLY, RAR, and BAR were natural log-transformed before analysis. HRs for continuous variables are presented per 1-standard deviation (SD) increase in each ln-transformed index. Model 1 was unadjusted. Model 2 was adjusted for age, sex, race/ethnicity, marital status, poverty-to-income ratio, and educational level. Model 3 was additionally adjusted for smoking status, alcohol consumption, and physical activity. Model 4 was further adjusted for body mass index, stroke, angina, hypertension, hyperlipidemia, diabetes mellitus, alanine aminotransferase, aspartate aminotransferase, total bilirubin, estimated glomerular filtration rate, hemoglobin, and cancer history.

**Abbreviations:** BAR, blood urea nitrogen–albumin ratio; CALLY, C-reactive protein–albumin–lymphocyte index; CI, confidence interval; HR, hazard ratio; RAR, red cell distribution width–albumin ratio; SD, standard deviation.

**Supplementary Table S15. Sensitivity analysis restricted to NHANES 2005–2010: associations of ln CALLY, ln RAR, and ln BAR with all-cause and cardiovascular mortality among adults with preclinical heart failure**

| Characteristics | Participants, n | Events, n (%) | Person-years | Model 1 | | Model 2 | | Model 3 | | Model 4 | |
| --- | --- | --- | --- | --- | --- | --- | --- | --- | --- | --- | --- |
|  |  |  |  | HR (95% CI) | P value | HR (95% CI) | P value | HR (95% CI) | P value | HR (95% CI) | P value |
| All-cause mortality |  |  |  |  |  |  |  |  |  |  |  |
| ln CALLY (per SD) | 8077 | 1535 (19) | 87158.8 | 0.83 (0.79–0.87) | <0.001 | 0.82 (0.78–0.87) | <0.001 | 0.83 (0.79–0.88) | <0.001 | 0.84 (0.80–0.89) | <0.001 |
| Q1 | 1987 | 466 (23.5) | 20736.3 | 1.00 (Ref) |  | 1.00 (Ref) |  | 1.00 (Ref) |  | 1.00 (Ref) |  |
| Q2 | 1916 | 374 (19.5) | 20673.7 | 0.80 (0.70–0.92) | 0.002 | 0.73 (0.63–0.83) | <0.001 | 0.72 (0.63–0.83) | <0.001 | 0.76 (0.66–0.87) | <0.001 |
| Q3 | 2054 | 349 (17) | 22483.8 | 0.69 (0.60–0.79) | <0.001 | 0.61 (0.53–0.70) | <0.001 | 0.62 (0.54–0.71) | <0.001 | 0.65 (0.57–0.75) | <0.001 |
| Q4 | 2120 | 346 (16.3) | 23265.1 | 0.66 (0.57–0.76) | <0.001 | 0.64 (0.56–0.74) | <0.001 | 0.67 (0.59–0.78) | <0.001 | 0.66 (0.57–0.76) | <0.001 |
| ln RAR (per SD) | 8077 | 1535 (19) | 87158.8 | 1.42 (1.37–1.48) | <0.001 | 1.40 (1.34–1.46) | <0.001 | 1.39 (1.33–1.46) | <0.001 | 1.37 (1.30–1.44) | <0.001 |
| Q1 | 1723 | 183 (10.6) | 19466.8 | 1.00 (Ref) |  | 1.00 (Ref) |  | 1.00 (Ref) |  | 1.00 (Ref) |  |
| Q2 | 1753 | 247 (14.1) | 19516.8 | 1.35 (1.11–1.63) | 0.002 | 1.03 (0.85–1.25) | 0.765 | 1.03 (0.85–1.25) | 0.747 | 1.03 (0.85–1.25) | 0.775 |
| Q3 | 2303 | 434 (18.8) | 24894.9 | 1.87 (1.57–2.22) | <0.001 | 1.21 (1.02–1.45) | 0.031 | 1.17 (0.98–1.39) | 0.083 | 1.17 (0.98–1.39) | 0.092 |
| Q4 | 2298 | 671 (29.2) | 23280.3 | 3.13 (2.66–3.68) | <0.001 | 1.94 (1.63–2.30) | <0.001 | 1.83 (1.54–2.17) | <0.001 | 1.74 (1.45–2.08) | <0.001 |
| ln BAR (per SD) | 8077 | 1535 (19) | 87158.8 | 1.80 (1.72–1.89) | <0.001 | 1.12 (1.06–1.19) | <0.001 | 1.16 (1.10–1.23) | <0.001 | 1.11 (1.04–1.18) | 0.002 |
| Q1 | 2167 | 245 (11.3) | 24265.2 | 1.00 (Ref) |  | 1.00 (Ref) |  | 1.00 (Ref) |  | 1.00 (Ref) |  |
| Q2 | 2033 | 284 (14) | 22615.9 | 1.25 (1.05–1.48) | 0.011 | 0.83 (0.70–0.99) | 0.040 | 0.90 (0.76–1.07) | 0.248 | 0.93 (0.78–1.11) | 0.430 |
| Q3 | 1906 | 289 (15.2) | 21023.6 | 1.37 (1.15–1.62) | <0.001 | 0.67 (0.56–0.79) | <0.001 | 0.74 (0.62–0.89) | 0.001 | 0.75 (0.63–0.90) | 0.002 |
| Q4 | 1971 | 717 (36.4) | 19254.2 | 3.82 (3.31–4.42) | <0.001 | 1.05 (0.89–1.22) | 0.579 | 1.18 (1.00–1.38) | 0.044 | 1.08 (0.90–1.29) | 0.404 |
| Cardiovascular mortality |  |  |  |  |  |  |  |  |  |  |  |
| ln CALLY (per SD) | 8077 | 483 (6) | 87158.8 | 0.83 (0.76–0.91) | <0.001 | 0.82 (0.75–0.90) | <0.001 | 0.83 (0.75–0.91) | <0.001 | 0.86 (0.78–0.94) | 0.002 |
| Q1 | 1987 | 149 (7.5) | 20736.3 | 1.00 (Ref) |  | 1.00 (Ref) |  | 1.00 (Ref) |  | 1.00 (Ref) |  |
| Q2 | 1916 | 107 (5.6) | 20673.7 | 0.72 (0.56–0.92) | 0.009 | 0.63 (0.49–0.81) | <0.001 | 0.62 (0.48–0.80) | <0.001 | 0.65 (0.51–0.84) | 0.001 |
| Q3 | 2054 | 117 (5.7) | 22483.8 | 0.72 (0.57–0.92) | 0.008 | 0.61 (0.48–0.78) | <0.001 | 0.62 (0.48–0.79) | <0.001 | 0.69 (0.54–0.88) | 0.003 |
| Q4 | 2120 | 110 (5.2) | 23265.1 | 0.65 (0.51–0.84) | 0.001 | 0.63 (0.49–0.80) | <0.001 | 0.64 (0.50–0.83) | 0.001 | 0.67 (0.52–0.87) | 0.002 |
| ln RAR (per SD) | 8077 | 483 (6) | 87158.8 | 1.39 (1.30–1.49) | <0.001 | 1.34 (1.23–1.46) | <0.001 | 1.34 (1.23–1.46) | <0.001 | 1.25 (1.13–1.38) | <0.001 |
| Q1 | 1723 | 55 (3.2) | 19466.8 | 1.00 (Ref) |  | 1.00 (Ref) |  | 1.00 (Ref) |  | 1.00 (Ref) |  |
| Q2 | 1753 | 84 (4.8) | 19516.8 | 1.53 (1.09–2.15) | 0.015 | 1.13 (0.80–1.59) | 0.479 | 1.14 (0.81–1.61) | 0.448 | 1.09 (0.77–1.54) | 0.619 |
| Q3 | 2303 | 137 (5.9) | 24894.9 | 1.97 (1.44–2.69) | <0.001 | 1.20 (0.87–1.65) | 0.259 | 1.17 (0.85–1.61) | 0.329 | 1.10 (0.80–1.52) | 0.557 |
| Q4 | 2298 | 207 (9) | 23280.3 | 3.21 (2.38–4.32) | <0.001 | 1.82 (1.34–2.48) | <0.001 | 1.76 (1.29–2.40) | <0.001 | 1.48 (1.07–2.05) | 0.018 |
| ln BAR (per SD) | 8077 | 483 (6) | 87158.8 | 2.02 (1.86–2.19) | <0.001 | 1.22 (1.11–1.34) | <0.001 | 1.25 (1.13–1.38) | <0.001 | 1.19 (1.05–1.34) | 0.005 |
| Q1 | 2167 | 61 (2.8) | 24265.2 | 1.00 (Ref) |  | 1.00 (Ref) |  | 1.00 (Ref) |  | 1.00 (Ref) |  |
| Q2 | 2033 | 76 (3.7) | 22615.9 | 1.34 (0.96–1.88) | 0.087 | 0.85 (0.60–1.19) | 0.343 | 0.91 (0.64–1.28) | 0.571 | 0.92 (0.65–1.31) | 0.655 |
| Q3 | 1906 | 99 (5.2) | 21023.6 | 1.88 (1.37–2.58) | <0.001 | 0.83 (0.60–1.15) | 0.257 | 0.91 (0.65–1.26) | 0.554 | 0.90 (0.65–1.26) | 0.549 |
| Q4 | 1971 | 247 (12.5) | 19254.2 | 5.29 (4.00–7.01) | <0.001 | 1.24 (0.92–1.67) | 0.165 | 1.36 (1.00–1.84) | 0.050 | 1.22 (0.87–1.69) | 0.250 |

**Notes:** Analyses were restricted to participants enrolled in the NHANES 2005–2010 cycles. Hazard ratios (HRs) and 95% confidence intervals (CIs) were estimated using Cox proportional hazards regression models. CALLY, RAR, and BAR were natural log-transformed before analysis. HRs for continuous variables are presented per 1-standard deviation (SD) increase in each ln-transformed index. Model 1 was unadjusted. Model 2 was adjusted for age, sex, race/ethnicity, marital status, poverty-to-income ratio, and educational level. Model 3 was additionally adjusted for smoking status, alcohol consumption, and physical activity. Model 4 was further adjusted for body mass index, stroke, angina, hypertension, hyperlipidemia, diabetes mellitus, alanine aminotransferase, aspartate aminotransferase, total bilirubin, estimated glomerular filtration rate, hemoglobin, and cancer history.

**Abbreviations:** BAR, blood urea nitrogen–albumin ratio; CALLY, C-reactive protein–albumin–lymphocyte index; CI, confidence interval; HR, hazard ratio; RAR, red cell distribution width–albumin ratio; SD, standard deviation.

**Supplementary Table S16. Sensitivity analysis of the associations of ln CALLY, ln RAR, and ln BAR with all-cause and cardiovascular mortality after additional adjustment for NHANES survey cycle**

| Characteristics | Participants, n | Events, n (%) | Person-years | Model 1 | | Model 2 | | Model 3 | | Model 4 | |
| --- | --- | --- | --- | --- | --- | --- | --- | --- | --- | --- | --- |
|  |  |  |  | HR (95% CI) | P value | HR (95% CI) | P value | HR (95% CI) | P value | HR (95% CI) | P value |
| All-cause mortality |  |  |  |  |  |  |  |  |  |  |  |
| ln CALLY (per SD) | 14717 | 3801 (25.8) | 186678.5 | 0.82 (0.79–0.84) | <0.001 | 0.83 (0.80–0.86) | <0.001 | 0.85 (0.82–0.88) | <0.001 | 0.85 (0.82–0.88) | <0.001 |
| Q1 | 3673 | 1154 (31.4) | 44833.7 | 1.00 (Ref) |  | 1.00 (Ref) |  | 1.00 (Ref) |  | 1.00 (Ref) |  |
| Q2 | 3684 | 1000 (27.1) | 46993.7 | 0.82 (0.75–0.89) | <0.001 | 0.78 (0.72–0.85) | <0.001 | 0.81 (0.75–0.89) | <0.001 | 0.82 (0.75–0.89) | <0.001 |
| Q3 | 3680 | 883 (24) | 47098.4 | 0.72 (0.66–0.79) | <0.001 | 0.67 (0.61–0.73) | <0.001 | 0.70 (0.64–0.77) | <0.001 | 0.71 (0.65–0.78) | <0.001 |
| Q4 | 3680 | 764 (20.8) | 47752.8 | 0.62 (0.56–0.68) | <0.001 | 0.65 (0.59–0.71) | <0.001 | 0.69 (0.63–0.76) | <0.001 | 0.67 (0.61–0.74) | <0.001 |
| ln RAR (per SD) | 14717 | 3801 (25.8) | 186678.5 | 1.40 (1.36–1.44) | <0.001 | 1.34 (1.30–1.38) | <0.001 | 1.30 (1.26–1.34) | <0.001 | 1.29 (1.25–1.33) | <0.001 |
| Q1 | 3662 | 594 (16.2) | 51663.8 | 1.00 (Ref) |  | 1.00 (Ref) |  | 1.00 (Ref) |  | 1.00 (Ref) |  |
| Q2 | 3309 | 772 (23.3) | 43191.4 | 1.60 (1.44–1.78) | <0.001 | 1.18 (1.06–1.31) | 0.003 | 1.15 (1.04–1.29) | 0.009 | 1.18 (1.06–1.31) | 0.003 |
| Q3 | 4055 | 1116 (27.5) | 50195.5 | 2.03 (1.84–2.25) | <0.001 | 1.33 (1.20–1.48) | <0.001 | 1.27 (1.14–1.41) | <0.001 | 1.31 (1.18–1.45) | <0.001 |
| Q4 | 3691 | 1319 (35.7) | 41627.8 | 2.97 (2.69–3.27) | <0.001 | 1.96 (1.77–2.17) | <0.001 | 1.81 (1.62–2.01) | <0.001 | 1.84 (1.65–2.06) | <0.001 |
| ln BAR (per SD) | 14717 | 3801 (25.8) | 186678.5 | 1.83 (1.78–1.89) | <0.001 | 1.15 (1.11–1.19) | <0.001 | 1.13 (1.09–1.17) | <0.001 | 1.08 (1.04–1.13) | <0.001 |
| Q1 | 3649 | 533 (14.6) | 48010.0 | 1.00 (Ref) |  | 1.00 (Ref) |  | 1.00 (Ref) |  | 1.00 (Ref) |  |
| Q2 | 3661 | 655 (17.9) | 48566.5 | 1.21 (1.08–1.35) | 0.001 | 0.87 (0.78–0.98) | 0.020 | 0.92 (0.82–1.03) | 0.167 | 0.91 (0.81–1.02) | 0.102 |
| Q3 | 3698 | 898 (24.3) | 48549.3 | 1.66 (1.49–1.85) | <0.001 | 0.83 (0.74–0.92) | 0.001 | 0.88 (0.79–0.98) | 0.026 | 0.86 (0.76–0.96) | 0.007 |
| Q4 | 3709 | 1715 (46.2) | 41552.7 | 3.85 (3.49–4.24) | <0.001 | 1.11 (1.00–1.24) | 0.040 | 1.13 (1.02–1.26) | 0.023 | 1.02 (0.91–1.14) | 0.756 |
| Cardiovascular mortality |  |  |  |  |  |  |  |  |  |  |  |
| ln CALLY (per SD) | 14717 | 1226 (8.3) | 186678.5 | 0.82 (0.77–0.87) | <0.001 | 0.84 (0.79–0.89) | <0.001 | 0.86 (0.81–0.91) | <0.001 | 0.87 (0.82–0.93) | <0.001 |
| Q1 | 3673 | 366 (10) | 44833.7 | 1.00 (Ref) |  | 1.00 (Ref) |  | 1.00 (Ref) |  | 1.00 (Ref) |  |
| Q2 | 3684 | 323 (8.8) | 46993.7 | 0.84 (0.72–0.97) | 0.018 | 0.79 (0.68–0.92) | 0.002 | 0.82 (0.70–0.95) | 0.010 | 0.83 (0.71–0.96) | 0.013 |
| Q3 | 3680 | 295 (8) | 47098.4 | 0.76 (0.65–0.89) | 0.001 | 0.70 (0.60–0.81) | <0.001 | 0.73 (0.62–0.85) | <0.001 | 0.76 (0.65–0.89) | 0.001 |
| Q4 | 3680 | 242 (6.6) | 47752.8 | 0.62 (0.52–0.72) | <0.001 | 0.65 (0.55–0.77) | <0.001 | 0.69 (0.59–0.82) | <0.001 | 0.70 (0.59–0.83) | <0.001 |
| ln RAR (per SD) | 14717 | 1226 (8.3) | 186678.5 | 1.39 (1.33–1.46) | <0.001 | 1.32 (1.25–1.39) | <0.001 | 1.27 (1.20–1.35) | <0.001 | 1.26 (1.18–1.33) | <0.001 |
| Q1 | 3662 | 187 (5.1) | 51663.8 | 1.00 (Ref) |  | 1.00 (Ref) |  | 1.00 (Ref) |  | 1.00 (Ref) |  |
| Q2 | 3309 | 266 (8) | 43191.4 | 1.76 (1.46–2.12) | <0.001 | 1.24 (1.03–1.50) | 0.026 | 1.22 (1.01–1.48) | 0.036 | 1.23 (1.02–1.49) | 0.030 |
| Q3 | 4055 | 341 (8.4) | 50195.5 | 1.99 (1.66–2.38) | <0.001 | 1.23 (1.03–1.48) | 0.024 | 1.18 (0.98–1.42) | 0.080 | 1.20 (1.00–1.45) | 0.055 |
| Q4 | 3691 | 432 (11.7) | 41627.8 | 3.11 (2.62–3.70) | <0.001 | 1.92 (1.60–2.30) | <0.001 | 1.77 (1.46–2.15) | <0.001 | 1.74 (1.43–2.11) | <0.001 |
| ln BAR (per SD) | 14717 | 1226 (8.3) | 186678.5 | 2.03 (1.93–2.14) | <0.001 | 1.24 (1.17–1.32) | <0.001 | 1.23 (1.15–1.31) | <0.001 | 1.18 (1.10–1.28) | <0.001 |
| Q1 | 3649 | 138 (3.8) | 48010.0 | 1.00 (Ref) |  | 1.00 (Ref) |  | 1.00 (Ref) |  | 1.00 (Ref) |  |
| Q2 | 3661 | 191 (5.2) | 48566.5 | 1.36 (1.09–1.69) | 0.006 | 0.96 (0.77–1.20) | 0.714 | 1.02 (0.82–1.27) | 0.858 | 1.01 (0.81–1.26) | 0.924 |
| Q3 | 3698 | 290 (7.8) | 48549.3 | 2.06 (1.69–2.53) | <0.001 | 0.97 (0.79–1.20) | 0.803 | 1.05 (0.85–1.29) | 0.647 | 1.01 (0.82–1.25) | 0.893 |
| Q4 | 3709 | 607 (16.4) | 41552.7 | 5.27 (4.38–6.34) | <0.001 | 1.38 (1.13–1.67) | 0.001 | 1.42 (1.17–1.74) | 0.001 | 1.28 (1.03–1.58) | 0.026 |

**Notes:** Hazard ratios (HRs) and 95% confidence intervals (CIs) were estimated using Cox proportional hazards regression models. CALLY, RAR, and BAR were natural log-transformed before analysis. HRs for continuous variables are presented per 1-standard deviation (SD) increase in each ln-transformed index. Model 1 was unadjusted. Model 2 was adjusted for age, sex, race/ethnicity, marital status, poverty-to-income ratio, and educational level. Model 3 was additionally adjusted for smoking status, alcohol consumption, and physical activity. Model 4 was further adjusted for body mass index, stroke, angina, hypertension, hyperlipidemia, diabetes mellitus, alanine aminotransferase, aspartate aminotransferase, total bilirubin, estimated glomerular filtration rate, hemoglobin, and cancer history, and NHANES survey cycle, which was entered as a categorical variable.

**Abbreviations:** BAR, blood urea nitrogen–albumin ratio; CALLY, C-reactive protein–albumin–lymphocyte index; CI, confidence interval; HR, hazard ratio; RAR, red cell distribution width–albumin ratio; SD, standard deviation.

**Supplementary Table S17. Exploratory temporal validation of the associations of hs-CRP-based ln CALLY, ln RAR, and ln BAR with all-cause and cardiovascular mortality among adults with preclinical heart failure in NHANES 2015–2018**

| Characteristics | Participants, n | Events, n (%) | Person-years | Model 1 | | Model 2 | | Model 3 | | Model 4 | |
| --- | --- | --- | --- | --- | --- | --- | --- | --- | --- | --- | --- |
|  |  |  |  | HR (95% CI) | P value | HR (95% CI) | P value | HR (95% CI) | P value | HR (95% CI) | P value |
| All-cause mortality |  |  |  |  |  |  |  |  |  |  |  |
| ln CALLY (per SD) | 5580 | 175 (3.1) | 16254.7 | 0.78 (0.67–0.90) | 0.001 | 0.77 (0.67–0.89) | <0.001 | 0.78 (0.68–0.90) | 0.001 | 0.80 (0.69–0.92) | 0.002 |
| Q1 | 1395 | 59 (4.2) | 4005.1 | 1.00 (Ref) |  | 1.00 (Ref) |  | 1.00 (Ref) |  | 1.00 (Ref) |  |
| Q2 | 1394 | 33 (2.4) | 4055.7 | 0.55 (0.36–0.84) | 0.006 | 0.54 (0.35–0.83) | 0.005 | 0.55 (0.36–0.85) | 0.006 | 0.64 (0.41–0.98) | 0.042 |
| Q3 | 1396 | 41 (2.9) | 4173.3 | 0.67 (0.45–0.99) | 0.045 | 0.61 (0.41–0.91) | 0.016 | 0.63 (0.42–0.94) | 0.025 | 0.69 (0.45–1.03) | 0.072 |
| Q4 | 1395 | 42 (3) | 4020.7 | 0.71 (0.48–1.05) | 0.088 | 0.67 (0.45–1.01) | 0.056 | 0.71 (0.47–1.06) | 0.092 | 0.73 (0.48–1.11) | 0.142 |
| ln RAR (per SD) | 5580 | 175 (3.1) | 16254.7 | 1.68 (1.50–1.89) | <0.001 | 1.79 (1.56–2.06) | <0.001 | 1.79 (1.56–2.06) | <0.001 | 1.70 (1.47–1.97) | <0.001 |
| Q1 | 1376 | 20 (1.5) | 4547.8 | 1.00 (Ref) |  | 1.00 (Ref) |  | 1.00 (Ref) |  | 1.00 (Ref) |  |
| Q2 | 1392 | 31 (2.2) | 4174.2 | 1.69 (0.97–2.97) | 0.066 | 1.28 (0.73–2.26) | 0.389 | 1.27 (0.72–2.24) | 0.409 | 1.25 (0.71–2.22) | 0.437 |
| Q3 | 1415 | 43 (3) | 3942.2 | 2.50 (1.47–4.26) | 0.001 | 1.73 (1.01–2.96) | 0.046 | 1.73 (1.01–2.96) | 0.046 | 1.77 (1.02–3.07) | 0.041 |
| Q4 | 1397 | 81 (5.8) | 3590.5 | 5.20 (3.18–8.49) | <0.001 | 3.58 (2.15–5.96) | <0.001 | 3.56 (2.13–5.93) | <0.001 | 3.48 (2.05–5.93) | <0.001 |
| ln BAR (per SD) | 5580 | 175 (3.1) | 16254.7 | 1.90 (1.68–2.15) | <0.001 | 1.29 (1.12–1.50) | 0.001 | 1.32 (1.14–1.53) | <0.001 | 1.32 (1.14–1.52) | <0.001 |
| Q1 | 1391 | 31 (2.2) | 4268.3 | 1.00 (Ref) |  | 1.00 (Ref) |  | 1.00 (Ref) |  | 1.00 (Ref) |  |
| Q2 | 1397 | 28 (2) | 4191.1 | 0.92 (0.55–1.54) | 0.752 | 0.64 (0.38–1.08) | 0.093 | 0.65 (0.39–1.09) | 0.102 | 0.72 (0.42–1.22) | 0.219 |
| Q3 | 1384 | 25 (1.8) | 4004.8 | 0.86 (0.51–1.46) | 0.583 | 0.49 (0.29–0.84) | 0.009 | 0.52 (0.30–0.89) | 0.017 | 0.61 (0.35–1.05) | 0.075 |
| Q4 | 1408 | 91 (6.5) | 3790.6 | 3.32 (2.21–5.00) | <0.001 | 0.97 (0.63–1.51) | 0.906 | 1.05 (0.67–1.63) | 0.837 | 1.15 (0.73–1.82) | 0.547 |
| Cardiovascular mortality |  |  |  |  |  |  |  |  |  |  |  |
| ln CALLY (per SD) | 5580 | 41 (0.7) | 16254.7 | 0.66 (0.49–0.88) | 0.005 | 0.68 (0.52–0.91) | 0.008 | 0.68 (0.51–0.91) | 0.008 | 0.73 (0.54–0.97) | 0.030 |
| Q1 | 1395 | 16 (1.1) | 4005.1 | 1.00 (Ref) |  | 1.00 (Ref) |  | 1.00 (Ref) |  | 1.00 (Ref) |  |
| Q2 | 1394 | 7 (0.5) | 4055.7 | 0.43 (0.18–1.05) | 0.064 | 0.45 (0.19–1.11) | 0.085 | 0.45 (0.18–1.12) | 0.085 | 0.57 (0.23–1.42) | 0.229 |
| Q3 | 1396 | 9 (0.6) | 4173.3 | 0.54 (0.24–1.22) | 0.138 | 0.50 (0.22–1.14) | 0.101 | 0.51 (0.22–1.16) | 0.110 | 0.61 (0.26–1.42) | 0.256 |
| Q4 | 1395 | 9 (0.6) | 4020.7 | 0.56 (0.25–1.27) | 0.165 | 0.55 (0.24–1.26) | 0.156 | 0.56 (0.24–1.29) | 0.174 | 0.68 (0.29–1.64) | 0.392 |
| ln RAR (per SD) | 5580 | 41 (0.7) | 16254.7 | 1.51 (1.18–1.94) | 0.001 | 1.43 (1.04–1.97) | 0.029 | 1.45 (1.05–2.00) | 0.024 | 1.27 (0.90–1.80) | 0.176 |
| Q1 | 1376 | 4 (0.3) | 4547.8 | 1.00 (Ref) |  | 1.00 (Ref) |  | 1.00 (Ref) |  | 1.00 (Ref) |  |
| Q2 | 1392 | 8 (0.6) | 4174.2 | 2.18 (0.66–7.25) | 0.203 | 1.51 (0.45–5.04) | 0.505 | 1.47 (0.44–4.93) | 0.534 | 1.32 (0.39–4.49) | 0.658 |
| Q3 | 1415 | 13 (0.9) | 3942.2 | 3.76 (1.22–11.55) | 0.021 | 2.31 (0.74–7.20) | 0.151 | 2.28 (0.73–7.15) | 0.156 | 1.84 (0.57–5.92) | 0.309 |
| Q4 | 1397 | 16 (1.1) | 3590.5 | 5.10 (1.70–15.32) | 0.004 | 2.70 (0.87–8.42) | 0.086 | 2.82 (0.90–8.85) | 0.075 | 2.04 (0.63–6.67) | 0.236 |
| ln BAR (per SD) | 5580 | 41 (0.7) | 16254.7 | 2.08 (1.63–2.66) | <0.001 | 1.36 (1.02–1.83) | 0.039 | 1.38 (1.03–1.86) | 0.030 | 1.36 (1.02–1.82) | 0.036 |
| Q1 | 1391 | 6 (0.4) | 4268.3 | 1.00 (Ref) |  | 1.00 (Ref) |  | 1.00 (Ref) |  | 1.00 (Ref) |  |
| Q2 | 1397 | 7 (0.5) | 4191.1 | 1.19 (0.40–3.53) | 0.758 | 0.73 (0.24–2.19) | 0.569 | 0.70 (0.23–2.13) | 0.534 | 0.74 (0.24–2.31) | 0.607 |
| Q3 | 1384 | 8 (0.6) | 4004.8 | 1.42 (0.49–4.10) | 0.515 | 0.78 (0.26–2.29) | 0.648 | 0.78 (0.26–2.31) | 0.657 | 0.98 (0.32–3.01) | 0.967 |
| Q4 | 1408 | 20 (1.4) | 3790.6 | 3.75 (1.50–9.34) | 0.005 | 0.88 (0.34–2.30) | 0.798 | 0.92 (0.35–2.43) | 0.874 | 1.00 (0.37–2.76) | 0.993 |

**Notes:** Analyses were restricted to participants enrolled in NHANES 2015–2018. For this exploratory temporal validation analysis, CALLY was calculated using high-sensitivity C-reactive protein (hs-CRP) measurements. Hazard ratios (HRs) and 95% confidence intervals (CIs) were estimated using Cox proportional hazards regression models. CALLY, RAR, and BAR were natural log-transformed before analysis. HRs for continuous variables are presented per 1-standard deviation (SD) increase in each ln-transformed index. Model 1 was unadjusted. Model 2 was adjusted for age, sex, race/ethnicity, marital status, poverty-to-income ratio, and educational level. Model 3 was additionally adjusted for smoking status, alcohol consumption, and physical activity. Model 4 was further adjusted for body mass index, stroke, angina, hypertension, hyperlipidemia, diabetes mellitus, alanine aminotransferase, aspartate aminotransferase, total bilirubin, estimated glomerular filtration rate, hemoglobin, and cancer history. These results should be interpreted cautiously because the available mortality follow-up was shorter than that of the primary cohort, particularly for cardiovascular mortality.

**Abbreviations:** BAR, blood urea nitrogen–albumin ratio; CALLY, C-reactive protein–albumin–lymphocyte index; CI, confidence interval; HR, hazard ratio; RAR, red cell distribution width–albumin ratio; SD, standard deviation.

**Supplementary Table S18. Subgroup analyses of the associations of ln CALLY, ln RAR, and ln BAR with all-cause and cardiovascular mortality according to PHF component status and burden**

| Subgroup | Participants, n | Events, n (%) | Person-years | ln CALLY | | | ln RAR | | | ln BAR | | |
| --- | --- | --- | --- | --- | --- | --- | --- | --- | --- | --- | --- | --- |
|  |  |  |  | HR (95% CI) | P value | P for interaction | HR (95% CI) | P value | P for interaction | HR (95% CI) | P value | P for interaction |
| All-cause mortality |  |  |  |  |  |  |  |  |  |  |  |  |
| Obesity |  |  |  |  |  | 0.018 |  |  | 0.742 |  |  | 0.183 |
| No | 7234 | 2440 (33.7) | 88932.8 | 0.88 (0.84–0.91) | <0.001 |  | 1.27 (1.22–1.32) | <0.001 |  | 1.06 (1.01–1.12) | 0.020 |  |
| Yes | 7483 | 1361 (18.2) | 97745.8 | 0.79 (0.74–0.84) | <0.001 |  | 1.34 (1.27–1.42) | <0.001 |  | 1.17 (1.09–1.26) | <0.001 |  |
| Hypertension |  |  |  |  |  | 0.071 |  |  | 0.154 |  |  | 0.425 |
| No | 3152 | 406 (12.9) | 42026.1 | 0.77 (0.69–0.85) | <0.001 |  | 1.40 (1.27–1.54) | <0.001 |  | 1.21 (1.06–1.39) | 0.005 |  |
| Yes | 11565 | 3395 (29.4) | 144652.4 | 0.87 (0.84–0.90) | <0.001 |  | 1.27 (1.22–1.31) | <0.001 |  | 1.09 (1.04–1.14) | <0.001 |  |
| Diabetes mellitus |  |  |  |  |  | 0.956 |  |  | 0.332 |  |  | 0.133 |
| No | 11700 | 2646 (22.6) | 152156.5 | 0.86 (0.82–0.90) | <0.001 |  | 1.28 (1.23–1.33) | <0.001 |  | 1.03 (0.98–1.09) | 0.203 |  |
| Yes | 3017 | 1155 (38.3) | 34522.0 | 0.86 (0.81–0.91) | <0.001 |  | 1.26 (1.20–1.33) | <0.001 |  | 1.22 (1.12–1.32) | <0.001 |  |
| Number of PHF risk factors |  |  |  |  |  | 0.119 |  |  | 0.002 |  |  | 0.136 |
| 1 | 7752 | 1619 (20.9) | 101952.9 | 0.85 (0.80–0.89) | <0.001 |  | 1.32 (1.26–1.40) | <0.001 |  | 1.00 (0.94–1.06) | 0.991 |  |
| 2 | 4775 | 1252 (26.2) | 60438.5 | 0.91 (0.86–0.97) | 0.004 |  | 1.19 (1.12–1.26) | <0.001 |  | 1.17 (1.09–1.26) | <0.001 |  |
| ≥3 | 2190 | 930 (42.5) | 24287.1 | 0.86 (0.80–0.92) | <0.001 |  | 1.31 (1.22–1.40) | <0.001 |  | 1.14 (1.04–1.25) | 0.004 |  |
| ASCVD |  |  |  |  |  | 0.337 |  |  | 0.479 |  |  | 0.466 |
| No | 13170 | 2971 (22.6) | 170519.9 | 0.85 (0.82–0.89) | <0.001 |  | 1.28 (1.24–1.33) | <0.001 |  | 1.09 (1.04–1.14) | <0.001 |  |
| Yes | 1547 | 830 (53.7) | 16158.6 | 0.87 (0.81–0.94) | <0.001 |  | 1.26 (1.17–1.35) | <0.001 |  | 1.13 (1.03–1.24) | 0.007 |  |
| Cardiovascular mortality |  |  |  |  |  |  |  |  |  |  |  |  |
| Obesity |  |  |  |  |  | 0.083 |  |  | 0.689 |  |  | 0.544 |
| No | 7234 | 785 (10.9) | 88932.8 | 0.90 (0.84–0.97) | 0.004 |  | 1.23 (1.14–1.32) | <0.001 |  | 1.19 (1.09–1.31) | <0.001 |  |
| Yes | 7483 | 441 (5.9) | 97745.8 | 0.79 (0.70–0.89) | <0.001 |  | 1.32 (1.19–1.47) | <0.001 |  | 1.25 (1.10–1.42) | 0.001 |  |
| Hypertension |  |  |  |  |  | 0.349 |  |  | 0.367 |  |  | 0.077 |
| No | 3152 | 111 (3.5) | 42026.1 | 0.73 (0.60–0.89) | 0.002 |  | 1.37 (1.14–1.64) | 0.001 |  | 1.68 (1.27–2.21) | <0.001 |  |
| Yes | 11565 | 1115 (9.6) | 144652.4 | 0.87 (0.82–0.93) | <0.001 |  | 1.25 (1.17–1.33) | <0.001 |  | 1.19 (1.09–1.28) | <0.001 |  |
| Diabetes mellitus |  |  |  |  |  | 0.296 |  |  | 0.287 |  |  | 0.757 |
| No | 11700 | 838 (7.2) | 152156.5 | 0.85 (0.79–0.92) | <0.001 |  | 1.26 (1.17–1.36) | <0.001 |  | 1.13 (1.03–1.24) | 0.010 |  |
| Yes | 3017 | 388 (12.9) | 34522.0 | 0.90 (0.82–1.00) | 0.051 |  | 1.23 (1.12–1.36) | <0.001 |  | 1.35 (1.17–1.56) | <0.001 |  |
| Number of PHF risk factors |  |  |  |  |  | 0.181 |  |  | 0.236 |  |  | 0.969 |
| 1 | 7752 | 451 (5.8) | 101952.9 | 0.84 (0.76–0.92) | <0.001 |  | 1.27 (1.14–1.41) | <0.001 |  | 1.12 (0.99–1.27) | 0.072 |  |
| 2 | 4775 | 410 (8.6) | 60438.5 | 0.93 (0.83–1.03) | 0.165 |  | 1.22 (1.11–1.34) | <0.001 |  | 1.24 (1.09–1.42) | 0.002 |  |
| ≥3 | 2190 | 365 (16.7) | 24287.1 | 0.93 (0.83–1.04) | 0.217 |  | 1.16 (1.03–1.31) | 0.015 |  | 1.19 (1.04–1.37) | 0.015 |  |
| ASCVD |  |  |  |  |  | 0.028 |  |  | 0.024 |  |  | 0.896 |
| No | 13170 | 889 (6.8) | 170519.9 | 0.83 (0.78–0.89) | <0.001 |  | 1.30 (1.21–1.39) | <0.001 |  | 1.22 (1.11–1.33) | <0.001 |  |
| Yes | 1547 | 337 (21.8) | 16158.6 | 0.95 (0.85–1.06) | 0.367 |  | 1.13 (1.00–1.28) | 0.057 |  | 1.22 (1.06–1.42) | 0.007 |  |

**Notes:** Hazard ratios and 95% confidence intervals are presented per 1-standard deviation increase in each natural log-transformed index. Subgroup analyses were performed according to obesity, hypertension, diabetes mellitus, ASCVD, and the number of PHF components. The number of PHF components was categorized as 1, 2, or ≥3. P values for interaction were calculated by including multiplicative interaction terms between each index and the corresponding subgroup variable in the fully adjusted Cox proportional hazards models. Unless the stratification variable itself was used to define the subgroup, models were adjusted for age, sex, race/ethnicity, marital status, poverty-to-income ratio, educational level, smoking status, alcohol consumption, physical activity, body mass index, stroke, angina, hypertension, hyperlipidemia, diabetes mellitus, alanine aminotransferase, aspartate aminotransferase, total bilirubin, estimated glomerular filtration rate, hemoglobin, and history of cancer.

**Abbreviations:** ALT, alanine aminotransferase; ASCVD, atherosclerotic cardiovascular disease; AST, aspartate aminotransferase; BAR, blood urea nitrogen–albumin ratio; BMI, body mass index; CALLY, C-reactive protein–albumin–lymphocyte index; CI, confidence interval; CVD, cardiovascular disease; eGFR, estimated glomerular filtration rate; HR, hazard ratio; PHF, preclinical heart failure; PIR, poverty-to-income ratio; RAR, red cell distribution width–albumin ratio; SD, standard deviation.
